# Bioactivity Profiles of Progressively Ring‐Fluorinated Cyclohexyl Motifs in the WKYMVm Peptide as Formylpeptide FPR2 Agonists and in Keto‐Piperazines as Antitrypanosome Agents

**DOI:** 10.1002/cbic.202500384

**Published:** 2025-10-03

**Authors:** Mengfan He, Christina M. Thomson, Dawn Thompson, Vytautus Kuodis, Terry K. Smith, Sergio Dall’Angelo, David O’Hagan

**Affiliations:** ^1^ School of Chemistry and Centre for Biomolecular Sciences University of St. Andrews North Haugh St. Andrews Fife KY16 9S UK; ^2^ Institute of Medical Sciences School of Medicine, Medical Sciences and Nutrition University of Aberdeen Foresterhill Aberdeen AB25 2ZD UK; ^3^ Institute of Medical Sciences and John Mallard Scottish PET Centre School of Medicine, Medical Sciences and Nutrition University of Aberdeen Foresterhill Aberdeen AB25 2ZD UK

**Keywords:** anti‐trypanosomes, bioactives, organo‐fluorines, W‐peptides

## Abstract

A series of all‐*cis*‐ring‐fluorinated cyclohexylalanines with progressively increasing levels of vicinal fluorines, as well as 4‐fluorophenylalanine and pentafluoroarylphenylalanine is introduced into the WKYMVm peptide in place of its tyrosine residue, for assays against the G‐protein coupled formylpeptide receptor, FPR2. Selected all‐*cis*‐ring cyclohexylalanines of this class are also incorporated into a keto‐piperazine molecular scaffold to generate sp^3^‐rich derivatives for assays against the parasite *Trypanosoma brucei.* For these cyclohexylalanine analogs bioactivity trends correlate progressively with the levels of fluorination in each of the case studies. Notably, the all‐*cis* pentafluorocyclohexylalanine analog of the W‐peptide is least active perhaps correlating with the well‐known polarity of this ‘Janus face’ cyclohexane. Although the trend is also apparent in the anti‐trypanosomal assays of the keto‐piperazine derivatives, it is less so and some compounds are more active than the previously reported phenylalanine‐derived analog.

## Graphical abstract

All‐*cis*‐fluorinated cyclohexyl motifs with progressively increasing levels of fluorine are introduced into the WKYMVm peptide and a class of keto‐piperazine derivatives and assayed against the FPR2 receptor or the parasite *Trypanosoma brucei,* respectively. Increasing fluorination, reduced Log P's, however it generally results in poorer bioactivities, indicating an inverse relationship between cyclohexane ring polarity and bioactivity in each case.

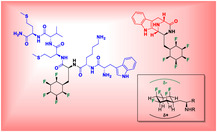

## 1 Introduction

**Figure 1:**
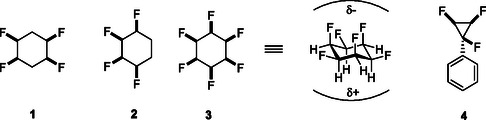
All‐*cis* fluorine substituted alicyclic rings generate polar aliphatic motifs.

Selective fluorination has proven to be of singular value in pharmaceuticals and bioactives development, to the point that presently around 20% of pharmaceuticals and 50% of agrochemicals products on the market contain at least one fluorine atom.^[^ ^1^ ^]^ The percentage of fluorinated small molecules in recent FDA approvals is consistently increasing too.^[^ ^2^ ^]^ Fluorine has unique properties in this context such as a low steric impact in replacing H or O for F, a strong and stable C—F bond, poor ability as a hydrogen bonding acceptor, and a high electronegativity which allows tuning of the electronic profile of a molecule.^[^ ^3^ ^]^ We have developed an interest in the properties of selectively fluorinated cyclohexane rings and continue to explore these motifs in the context of medicinal chemistry.^[^ ^4^ ^]^ The cyclohexane ring is a conspicuously lipophilic entity and when incorporated into bioactive candidates, it tends to raise molecular LogP and thus compromises optimal pharmacokinetic profiles. This can lead to rapid oxidative metabolism, an aspect associated with more lipophilic compounds.^[^ ^5^ ^]^ However selective fluorination of a cyclohexane ring, or other lipophilic motifs (cyclopropyl, tert‐butyl), generates fluoromethylene (‐CHF‐) or fluoromethyl (‐CH_2_F) carbons which either individually or in concert act to increase the overall molecular polarity and lower LogPs.^[^ ^6^, ^7^ ^]^ Thus selective fluorination of aliphatics offers a tool in medicinal chemistry design beyond just blocking sites of metabolism. A particularly dramatic manifestation of the properties of organic bound fluorine arises when all of the fluorines occupy one face (all‐*cis*) of a cyclohexane ring as this maximizes the molecular polarity, and particularly when axial C—F bonds align parallel. This polarization is reflected in the properties of, for example, *cis‐*1,2,4,5‐ **1** (mp = 109 °C, *μ* = 5.2D),^[4e]^ all *cis‐*1,2,3,4‐ **2** (mp = 83 °C, *μ* = 4.5D)^[4f]^ tetrafluoro‐cyclohexanes and all‐*cis*‐1,2,3,4,5,6‐hexafluorocyclohexane **3** (206 °C (dec), *μ* = 6.2 D).^[^ ^8^ ^]^ These are all crystalline compounds with high molecular dipoles (*μ*).^[^ ^3^, ^4^ ^]^ These have been termed Janus cyclohexanes,^[^ ^9^ ^]^ as they present electrostatically compensated faces pointing in opposite directions and extend to other alicyclic rings such as all‐*cis* 1,2,3‐trifluorocyclopropane **4** (**Figure** **1**).^[4c]^

**Scheme 1:**
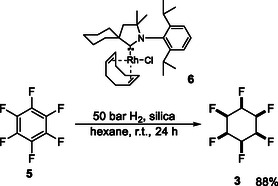
Aryl hydrogenation of hexafluorobenzene to **3** with Rh(CAAC)Cl catalyst **6**.^[^ ^11^ ^]^

This class of compounds is most conveniently accessed by high pressure fluoroaryl hydrogenations to generate fluorocyclohexanes, facilitated by a cyclic (amino)(alkyl)carbene (CAAC) rhodium catalyst **6**. Zeng's lab demonstrated that catalyst **6** could catalyze aryl hydrogenation of substituted arenes to cyclohexanes, carrying a range of functionality such as ketones, esters, or amides attached.^[^ ^10^ ^]^ Glorius's lab applied this protocol to aryl fluorides, and notably demonstrated conversion to fluorocyclohexanes as well as fluoropyridines to fluoropiperidines.^[^ ^11^ ^]^ An efficient hydrogenation of hexafluorobenzene **5** to generate **3** was accomplished.^[11b]^ Until that development, aryl‐hydrogenations with more than one fluorine, were always accompanied by fluoride ion elimination, and therefore Glorius's approach has opened up access to a diversity of multiply fluorinated cyclohexanes from fluoroarenes (**Scheme** **1**).

**Figure 2:**
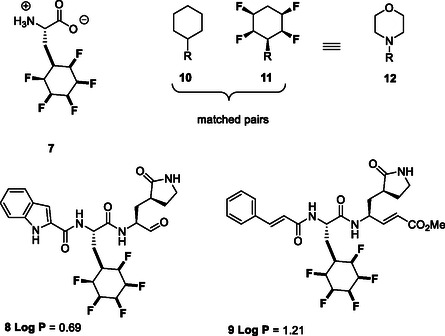
All‐*cis* fluorocyclohexane motifs.

We recently reported the synthesis of amino acid **7** by hydrogenation of the corresponding N‐Boc protected pentafluoroaryl l‐phenylalanine methyl ester with catalyst **6**, and then deprotection.^[6a]^ This amino acid was then selectively N‐Boc protected and incorporated into a range of small molecule antiviral candidates such as **8** and **9** via a peptide coupling strategy. A notable feature to emerge was that the incorporation of the pentafluorocyclohexyl motif into **8** and **9** did not increase in lipophilicity and the drug candidates retained LogP values in the 0–1.5 range. In a separate study from an industry lab,^[12a]^ the all‐*cis* tetrafluoro‐cyclohexyl moiety **11** ^[12b]^ was incorporated into 16 scaffolds and compared against analogous cyclohexyl matched pairs **10**. The overall analysis concluded that this tetrafluorocyclohexyl motif most resembled the morpholine moiety **12** in overview, after a range of pharmacokinetic properties were explored (**Figure** **2**).^[12a]^

**Figure 3:**
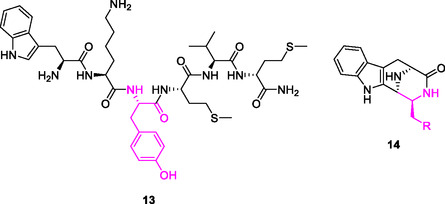
WKYMVm peptide **13** and 6‐5‐6‐6 fused carboline scaffold **14**.

In this article, we extend our analysis of these fluorocyclohexyl motifs and have selected to substitute the pentafluorocyclohexyl amino acid **7** and also analogous amino acids with lesser fluorines on the ring into known bioactives. In this context a series of derivatives of, WKYMVm (Trp‐Lys‐Tyr‐Met‐Val‐d‐Met‐NH_2_) **13** are prepared. WKYMVm is a modified synthetic hexapeptide, which is a highly potent formyl‐peptide receptor type 2 (FPR2) agonist where it exerts an immunoregulatory effect (**Figure** **3**).^[^ ^13^ ^,^ ^14^ ^]^

**Figure 4:**
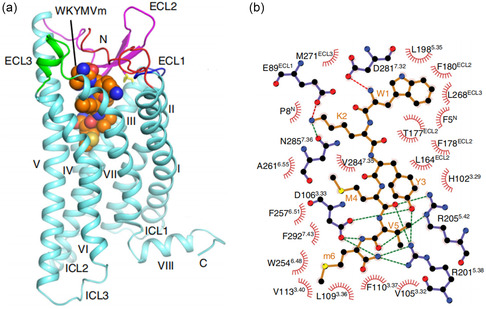
a) Representation of the FPR2 receptor structure with bound W‐peptide **13** from cryo‐EM. b) Flat representation of interactions between FPR2 and WKYMVm **13**. Figures are adapted from Chen et al.^[^ ^17^ ^]^

The formyl peptide receptors (FPRs) belong to the G protein‐coupled receptor family. These chemo‐attractant receptors are mainly involved in host defense and inflammation, and they contain three human isoforms: FPR1, FPR2/ALX, and FPR3.^[^ ^15^ ^]^ FPR2 also described as the lipoxin A4 receptor (ALX), recognizes a variety of ligands and is expressed in various cell types including neutrophils, macrophages, fibroblasts, and endothelial cells.^[^ ^16^ ^]^ Chen et al. in 2020 reported the cryo‐EM structure of the WKYMVm bound to the FPR2 receptor (**Figure** **4**).^[^ ^17^ ^]^ The whole structure participates in key contacts as it is located in a deep binding pocket within the receptor.

This work focused on swapping the Tyr (Y) residue of the WKYMVm peptide **13** with selectively fluorinated cyclohexane containing amino acids to assess the relative binding affinity of the analogs to FPR2 receptor. Tyr (Y3) was selected for replacement, given the broadly similar steric profile between the aryl and cyclohexane side chains.^[^ ^18^ ^]^

Alongside studies on WKYMVm **13**, attention also turned to the 6‐5‐6‐6 fused carboline scaffold **14**, various derivatives of which were recently shown to display promising antiparasitic activity against the neglected tropical diseases that cause human African trypanosomiasis (African sleeping sickness) and Chagas’ disease, the protozoan parasites *Trypanosoma brucei* and *Trypanosoma cruzi,* respectively.^[^ ^19^ ^]^ These and similar protozoan diseases that affect the tropics and subtropics impact both humans and animals, causing a social and economic burden on some of the poorest countries in the world.^[^ ^20^ ^]^ Current therapies are inadequate, expensive and they are hard to administer. They have low efficacy and drug resistance is an increasing issue, therefore new drug leads are welcome.^[^ ^21^, ^22^ ^]^

In this study, we report the preparation of analogs of **14**, modified as highlighted, with variously fluorinated cyclohexane rings to investigate the effect of increasingly polarized cyclohexane rings on bioactivity.

## 2 Results and Discussion

### 2.1 Synthesis of Ring‐Fluorinated Cyclohexane Derivatives of the Peptide WKYMVm

**Scheme 2:**
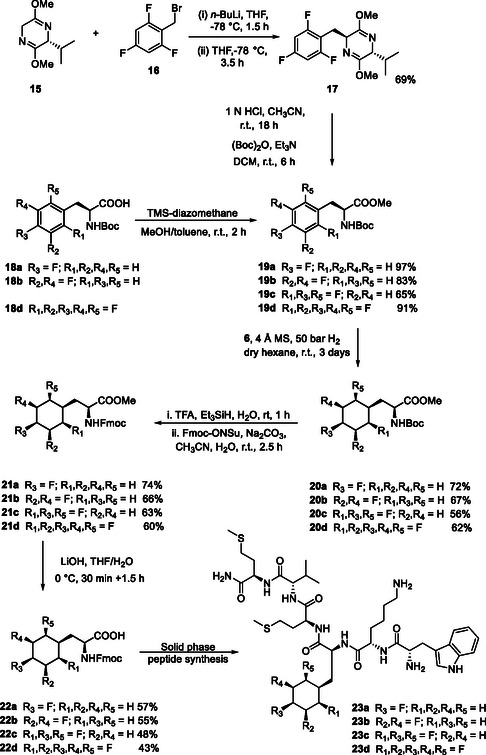
Synthesis route to keto‐piperizines **28a**,**b** and **d**.

The synthesis protocols, outlined in **Scheme** **2** started with TMS‐diazomethane mediated esterifications^[^ ^23^ ^]^ of the variously fluorinated N‐Boc protected phenylalanines **18a**,**b** and **d** to generate methyl esters **19a,b** and **d**. The trifluorinated amino acid ester **18c** was prepared separately via a Schöllkopf^[^ ^24^ ^]^ asymmetric amino acid synthesis protocol as the corresponding trifluorophenylalanine was not available. Accordingly alkylation of **15** with 2,4,6‐trifluorobenzylbromide **16** generated **17** as a single diastereoisomer. Hydrolysis and N‐Boc protection afforded methyl ester **18c**. Each of the protected amino acids **19a–d** was subjected to aryl hydrogenation with Rh catalyst **6**, and this generated the corresponding fluorinated cyclohexanes **20a–d**. This hydrogenation can be to one face or another of the aryl ring, always giving a *syn*‐stereochemistry for all substituents around the ring. It follows that a single stereoisomer is generated in all cases, despite which face is hydrogenated, because the fluorine substituents are arranged symmetrically around the ring. In the case of **20a** the reaction progressed efficiently (72% yield). It was clear however from ^19^F{^1^H} NMR that there were two aliphatic fluorine peaks in a ratio of 7:1. VT‐NMR (≈80 °C) did not result in a coalescence of the peaks and therefore it is concluded that they are diastereoisomers rather than conformational isomers, the major of which is assumed to have the *cis*‐stereochemistry **20a**. In all other cases, only a single stereoisomer was generated after aryl hydrogenation, giving the all‐*cis* stereochemistry in each case. For the synthesis of the W‐peptides, the N‐Boc protecting groups were switched to Fmoc to give esters **21a–d** and then these were hydrolyzed to the free carboxylic acids **22a–d** in preparation for solid phase peptide synthesis (SPPS).^[^ ^25^ ^]^ Consequently, the W‐peptide analogs **23a–d, 24** and **25** were generated by microwave‐assisted SPPS. Peptides **24** and **25** were prepared introducing either the mono‐ or penta‐fluoroaryl Fmoc‐phenylalanines, respectively, as comparative motifs. After cleavage, all of the W‐peptide analogs were purified by preparative high performance liquid chromatography (HPLC) 22 and their identity and purity were confirmed by liquid chromatography‐mass spectrometry (LC‐MS) (see Supporting Information).

**Figure 5:**
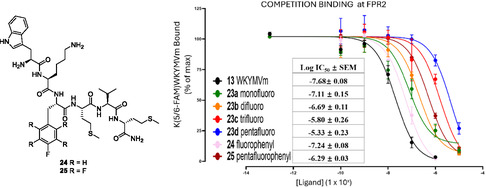
Competition binding analysis of WKYMVm **13**, **23a–d**, **24** and **25** against the FPR2 receptor with comparative Log‐IC_50_ values.

Competition binding assays were conducted to assess the relative affinity of WKYMVm **13** and analogs **23a–d**, **24** and **25** at the FPR2 receptor against a K(5/6‐FAM)‐WKYMVm peptide ligand, where (5/6‐FAM)‐indicates a carboxyfluorescein moiety.^[^ ^26^, ^27^ ^]^ Briefly, HEK293 cells expressing FPR2 were added to a mixture of K(5/6‐FAM)‐WKYMVm (10 nM) in combination with varying concentrations (0–10 μM) of the competing ligands and incubated for 90 min at 4 °C in the dark to allow equilibrium binding. Subsequently, mean fluorescence was measured using flow cytometry (Attune NxT). Data was expressed as a percentage of the maximum binding (i.e., no competitor) and errors were determined as a result of three independent experiments performed in duplicate (**Figure** **5**).

**Figure 6:**
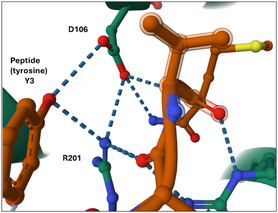
Hydrogen bonding contacts between the Y3 phenol of 13 and D106 and R201 of peptide **13**. from the cocrystal structure PDB 6LW5.^[^ ^17^ ^]^

The original WKYMVm **13** remained the highest affinity peptide, followed by the 4‐fluorophenyl‐modified peptide **24**. Notably replacing the tyrosine aryl‐OH with aryl‐F had no significant impact on the IC_50_ (Figure 5). Given that F has some aspects of an O mimetic, this suggests a limited importance of the hydrogen bonding donor ability of the tyrosine residue for binding. The co‐crystal structure of **13** with FPR2 indicates a hydrogen bond network between the peptide tyrosine (Y3), with Asp‐106 and Arg‐201.^[^ ^17^ ^]^ In particular the structure indicates that the phenol of Y3 will act as a hydrogen (donor) to the carboxylate of Asp‐106 as illustrated in **Figure** **6**. Clearly in peptide **24** this interaction is compromised although the fluorine may still pick up an electrostatic contact to the guanidinium residue of Arg‐201.

Among the four fluorocyclohexane analogs **23a–d**, the assays demonstrated a clear and progressive reduction in binding affinity with the level of fluorination. The monofluoro‐ cyclohexane **23a** had the highest affinity but significantly weaker than that of the WKYMVm **13**, progressing through difluoro‐ **23b** and trifluoro **23c** until the pentafluoro‐ **23e** cyclohexane which had the weakest affinity. There was an order of magnitude reduction in binding affinity between the difluoro‐ **23b** and the trifluoro‐ **23c** analogs (10^−7^ M versus 10^−6^ M, respectively). These data indicate that the more polar the cyclohexane, the weaker the binding. Notably, the introduction of the pentafluoroaryl motif caused a significant decrease in the binding affinity compared to the 4‐fluoro‐phenyl derivative (10^−6^ M vs 10^−7^ M).

**Figure 7:**
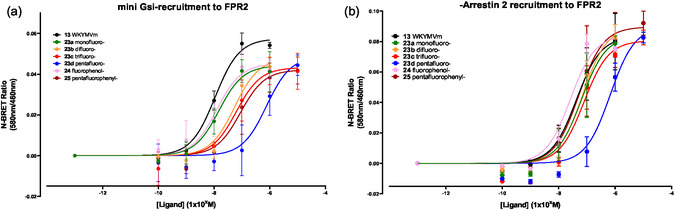
BRET analysis of a) mini‐Gsi protein and b) *β*‐Arrestin recruitment with FPR2 activated by WKYMVm **13**, **23a–d**, **24** and **25**.

Since these modifications of WKYMVm altered the relative affinity of the compounds to bind to FPR2, a further series of bioluminescence resonance energy transfer (BRET) assays were conducted to determine if there was any change in efficacy, specifically the ability to recruit molecular effectors such as G‐protein or *β*‐arrestin.^[^ ^28^, ^29^ ^]^ Briefly, HEK293 cells were transiently transfected with FPR2‐Rluc8 (donor) in combination with and either mGsi‐Venus or *β*‐arrestin 2‐YFP (acceptors) and replated after 24 h into white opaque 96‐well plates. Cells were assayed 24 h later at 37 °C, in HEPES‐buffered saline solution (HBSS) with coelenterazine H (5 μM). Cells were then stimulated with increasing concentrations of WKYMVm or analogs. Luminescence and fluorescence values were measured at 460/30 nm (FPR2) and 480/40 nm (mini‐Gsi or *β*‐arrestin protein), respectively, and the acceptor: donor ratio was determined, normalized by subtraction of the ratio observed in untreated cells (**Figure** **7**).

The FPR2 receptor belongs to the G protein‐coupled receptor family^[^ ^30^ ^]^ which, following ligand binding and activation, undergo a conformational change resulting in G‐protein coupling and the recruitment of molecular machinery such a *β*‐arrestins. BRET allows quantification of these interactions and can elucidate their dynamics.^[^ ^29^ ^]^ WKYMVm **13** was again the most efficacious at FPR2 as revealed by a concentration dependent increase in both Gi and *β*‐arrestin recruitment (Figure 7A,B), with the 4‐fluorophenylalanine analog **24** most similar. However, the mono‐ **23a**, di‐ **23b**, and tri‐ **23c** fluoro cyclohexyl analogs had comparable efficacy for either Gi or *β*‐arrestin recruitment, while the more polar pentafluorocyclohexane analog **23d** proved to be significantly weaker. This trend is consistent with the competition binding assays.

### 2.2 Tryptophan Keto‐Piperazines with Variously Ring Fluorinated Cyclohexylalanines

**Scheme 3:**
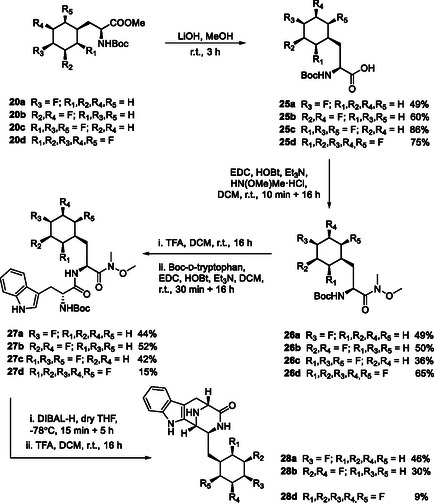
Synthetic routes to introduce Janus face amino acids into W‐peptides **23a–d**.

A second aspect of this study focused on introducing ring fluorinated cyclohexyl analogs into bridged tryptophan keto‐piperazines. Representatives of this alkaloid class were recently found to display antitrypanosomal activity.^[^ ^19^, ^31^ ^]^ One aspect of that design was to proactively introduce a sp^3^ rich 6‐5‐6‐6 heterocyclic scaffold. The scaffold is prepared by an intramolecular Pictet–Splenger cyclization^[^ ^32^ ^]^ of a tryptophan containing dipeptide. In this case we have coupled ring fluorinated cyclohexylalanines with tryptophan as to generate keto‐piperazines **28a**,**b** and **28d** as illustrated in **Scheme** **3**.

Accordingly N‐Boc amino acid esters **20a–d** were hydrolyzed to their free carboxylic acids **25a–d** and then progressed in each case to the corresponding Weinreb–Nahm amides^[^ ^33^ ^]^ **26a–d**. N‐Boc deprotection followed by coupling to N‐Boc‐tryptophan generated dipeptides **27a–d** as the required precursors for cyclization. The intramolecular Pictet–Spengler condensations, followed a previous protocol^[^ ^19^ ^]^ and 4‐monofluoro **28a**, 3,5‐difluoro‐ **28b**, and 2,3,4,5,6‐pentafluoro‐ **28d** keto‐piperazines could be prepared following this method. It is noteworthy that the pentafluoro‐ analog was accompanied by what appeared to be a high level of defluorinated side products, however **28d** was conveniently purified by HPLC rather than column chromatography. The trifluorocyclohexane **28c** could not be obtained cleanly by this route and proved susceptible to decomposition. Thus, 4‐monofluoro‐ **28a**, 3,5‐difluoro‐ **28b**, and 2,3,4,5,6‐pentafluoro‐ **28d** cyclohexyl‐amino acid tryptophan diketopiperazines were prepared and explored in antitrypanosomal assays. Given the importance of molecular lipophilicity in the context of medicinal chemistry, a Log P assessment (HPLC) was made of the diketopiperazines products **28a**, **28b**, and **28d** as illustrated in **Table** **1**. As anticipated, there is an increase in polarity (LogP reduction) as the number of fluorines increases. We also know from previous work^[6b]^ that the Ph ring is more polar than cyclohexane but less polar than the progressively fluorinated cyclohexane rings. The keto‐piperazines **28a**, **28b**, and **28d** were assayed^[^ ^20^ ^]^ against growing cells of *T. brucei* and *T. cruzi,* as well as the human cell line, HeLa and the data is presented in Table 1.

**Table 1:** Trypanocidal and mammalian activities (EC_50_'s) of keto‐piperazines **28a**, **28b**, and **28d**.

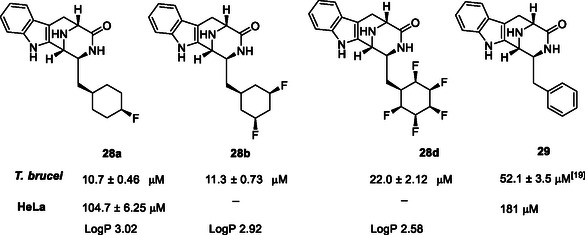


In the event the anti‐trypanocidal activity of the three tested keto‐piperazines against *T. brucei* were all more active than that of the previously reported phenylalanine derived keto‐piperazine **29**.^[^ ^19^ ^]^ The best compound was judged to be **28a**, and it had a selectivity for *T. brucei* over HeLa of ≈10:1 which is at a threshold level set by the Drugs of Neglected Diseases initiative (DNDi) for selectivity. It is notable that the least active compound **28d** has the most highly polarized cyclohexane ring.

## 3 Conclusions

The study presents the synthesis and incorporation of ring fluorinated cyclohexylalanine amino acids into the W‐peptide and a relatively complex keto‐piperazine heterocyclic scaffold. In each case the parent compounds are known bioactives, either agonists of the FPR2 receptor (WKYMVm) or they display antitrypanosomal activity (keto‐piperazine). In general, the fluorinated cyclohexane products showed modest to good activities, but as the number of fluorines on the cyclohexane ring increased, there was a noticeable decrease in the inherent activity. This is presumably as polarity is increasing and the ring system is acquiring its own unique characteristics relative to hydrophobic or aromatic rings. We present amino acid building blocks, containing these fluorocyclohexyl ring systems, which can be prepared and introduced into advanced bioactive structures for bioassay studies. However, they are sufficiently unique that they should be introduced at the beginning of drug discovery programmes and not used as ‘‘replacements’’ as they do not appear to offer an obvious bioisostere of any current functional grouping frequently found in drug molecules.

## Conflict of Interest

The authors declare no conflict of interest.

## Supporting information

Supplementary Material

## Data Availability

The data that support the findings of this study are available in the supplementary material of this article.
