# Supplementary material for: Bioactivity Profiles of Progressively Ring‐Fluorinated Cyclohexyl Motifs in the WKYMVm Peptide as Formylpeptide FPR2 Agonists and in Keto‐Piperazines as Antitrypanosome Agents
Source: Chembiochem. 2025 Oct 3;26(22):e202500384. doi: 10.1002/cbic.202500384 (PMC12631002; doi:10.1002/cbic.202500384)
Supplement: Supplementary file 1 — Supplementary Material [file CBIC-26-e202500384-s001.pdf]

SUPPLEMENTARY INFORMATION

Bioactivity profiles of progressively ring-fluorinated cyclohexyl motifs in the WKYMVm peptide as formylpeptide FPR2 agonists and in keto-piperazines as *anti*-trypanosome agents.

Mengfan He,[a] Christina M. Thomson,[b] Dawn Thompson,[b]* Vytautus Kuodis,[a] Terence K. Smith,[a] Sergio Dall'Angelo,[c]* and David O'Hagan [a]*

[a] School of Chemistry and Centre for Biomolecular Sciences,

University of St. Andrews, North Haugh, St. Andrews, Fife, KY16 9S, UK.

E-mail: do1@st-andrews.ac.uk

[b] Institute of Medical Sciences, University of Aberdeen, School of Medicine, Medical Sciences and Nutrition, Foresterhill, Aberdeen, AB25 2ZD, UK.

E-mail: dthompson@abdn.ac.uk

[c] John Mallard Scottish PET Centre, School of Medicine, Medical Sciences and Nutrition, University of Aberdeen, Foresterhill, Aberdeen, AB25 2ZD, UK.

E-mail: s.dallangelo@abdn.ac.uk

Table of Contents

General S-2

Synthesis protocols and NMR data S-4

NMR Spectra S-32

Additional experimental procedures.. S-96

Synthesis of Janus face W-peptide .... S_96

Bioactivities assays of Janus face W-peptide S-97

Bioactivities assays of Janus face Tryptophan keto-piperazines S-99

References.. S-100

General

NMR Spectra were recorded on Bruker AVIII 500, AVIII-HD 500 or AVIII-HD 700 spectrometer. NMR analyses were carried out at room temperature in indicated deuterated solvents unless otherwise noted. Chemical shift data are reported as *δ* in units of ppm relative to respective NMR solvent. Coupling constant *J* was reported in Hz. ^{1}H, ^{13}C, ^{19}F NMR spectra were recorded at 470 MHz with and without ^{1}H decoupling, relative to CCl_3F (δF = 0.00 ppm). Structural assignments were made with additional information from gCOSY, gHSQC, and gHMBC experiments in conjunction with ^{1}H, ^{13}C, and ^{19}F NMR data. Multiplicities are indicated by: s for singlet, d for doublet, t for triplet, q for quartet, p for septet and m for multiplet and br. for the broad brand.

All reactions were carried out under an argon atmosphere with standard Schlenk techniques unless otherwise specified. The reaction glassware was flame dried or oven dried and cooled under vacuum. Commercially available chemicals were purchased from Acros, Alfa Aeser, Fisher Scientific,

Fluorochem, Sigma Aldrich, Strem Chemicals, and TCI (UK) and used as received unless otherwise stated.

DCM and THF were dried and deoxygenated using an MBraun SPS-800 solvent system. Room temperature refers to the temperature range 15-25 ºC. *In vacuo* refer to the use of rotary evaporator with membrane pump at 30-50 mbar Analytical thin-layer chromatography was carried out on aluminium backed Merck TLC silica gel 60 F254 plates. These plates were visualised using UV light at 254 nm wavelength, dyed by potassium permanganate or phosphomolybdic acid followed by air dryer heating. Flash column chromatography was performed with Sigma-Aldrich silica gel, 60 Å pore size and 230-400 mesh, 40-63 µm particle size under 5 psi compressed air. High resolution mass spectra were recorded on a Thermo Scientific Exactive orbitrap mass spectrometer by the University of St Andrews, UK.

Synthesis protocols and NMR data

Esterification of fluorinated (*S*)-phenylalanine (General Procedure A)

TMS-diazomethane (2M solution in hexanes, max. 2 eq.) was added dropwise to a solution of (*S*)-Boc-para-fluorophenylalanine (1 eq.) in dry MeOH/toluene (1/1 v/v) at r.t. and the mixture was stirred until TLC analysis revealed a completed reaction after 4 h. The mixture was concentrated under reduced pressure and the product was purified by chromatography.

Boc protected (*S*)-phenylalanine methyl ester (19a)

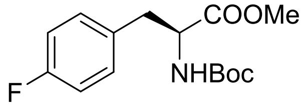

Ester **19a** was prepared according to General Procedure A from TMS-diazomethane (max. 2 mL, max. 4 mmol), and (*S*)-Boc-para-fluorophenylalanine **18a** (283.3 mg, 1 mmol) in dry MeOH/toluene (6 mL) and was purified by chromatography (hexane : ethyl acetate = 30 : 1 to 20 : 1 to 10 : 1 and TLC hexane : ethyl acetate = 6 : 1) to give the product as a white crystalline solid (290 mg, 97%). ^{1}H NMR (400 MHz, Chloroform-*d*) δ 7.14 – 6.89 (m, 4H), 4.98 (d, *J* = 8.3 Hz, 1H), 4.56 (m, *J* = 6.6 Hz, 1H), 3.71 (s, 3H), 3.17 – 2.93 (m, 2H), 1.41 (s, 9H). ^{19}F{^{1}H} NMR (377 MHz, Chloroform-*d*) δ -115.9. HRMS (ES^{+}) *m/z*: [M + Na]$^{+}$ calculated for $C_{15}H_{20}FNO_{4}Na$ 320.1269; found 320.1268.

Data identical to; F-M Meyer, S. Liras, A. Guzman-Perez, C. Perreault, J.Bian, K. James, *Org. Lett.,* **2010**, *12*, 3870–3873.

Boc protected (*S*)-phenylalanine methyl ester (19b)

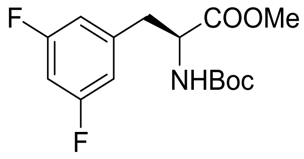

Ester **19b** was prepared according to General Procedure A from TMS-diazomethane (max. 6.5 mL, max. 13 mmol), and (*S*)-Boc-3,5-difluorophenylalanine **18b** (994 mg, 3.3 mmol) in dry MeOH/toluene (12 mL) and was purified by chromatography (hexane : ethyl acetate = 30 : 1 to 20 : 1 to 10 : 1 and TLC hexane : ethyl acetate = 6 : 1) to give the product as a white crystalline solid (863.2 mg, 83%). ^{1}H NMR (400 MHz, Chloroform-*d*) δ 6.75 – 6.60 (m, 3H), 5.02 (d, *J* = 8.5 Hz, 1H), 4.58 (m, *J* = 7.3 Hz, 1H), 3.74 (s, 3H), 3.13 (dd, *J* = 5.5, 13.9 Hz, 1H), 3.01 (dd, *J* = 6.2, 13.9 Hz, 1H), 1.43 (s, 9H). ^{13}C NMR (126 MHz, Chloroform- *d*) δ 171.9, 163.1 (d, $^{1}J_{CF}$ = 249Hz), 155.1, 140.1 (t, $^{2}J_{CF}$ = 10.1 Hz), 112.3, 102.7, 80.3, 54.2, 52.6, 38.2, 28.4. ^{19}F{^{1}H} NMR (376 MHz, Chloroform-*d*) δ -110.0. HRMS (ES^{+}) *m/z*: [M + Na]$^{+}$ calculated for $C_{15}H_{19}F_2NO_4Na$ 338.1174; found 338.1176.

Data identical to; A. J. Ross, F. Dreiocker, M. Sch€afer, J. Oomens, A. J. H. M. Meijer,B. T. Pickup, R. F. W. Jackson, *J. Org Chem.,* **2011**, *76*, 1727–1734.

Boc protected (*S*)-phenylalanine methyl ester (19d)

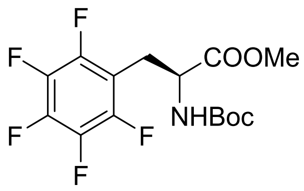

Ester **19d** was prepared according to General Procedure A from TMS-diazomethane (max. 5.6 mL, max. 11.3 mmol), and

(*S*)-Boc-pentafluorophenylalanine **18d** (1 g, 2.81 mmol) in dry MeOH/toluene (18 mL) and was purified by chromatography (hexane : ethyl acetate = 30 : 1 to 20 : 1 to 10 : 1 and TLC hexane : ethyl acetate = 6 : 1) to give the product as a white crystalline solid (945.9 mg, 91%). ^{1}H NMR (400 MHz, Chloroform-*d*) δ 5.12 (d, *J* = 8.2 Hz, 1H), 4.59 (m, *J* = 6.9 Hz, 1H), 3.78 (s, 3H), 3.33 (dd, *J* = 5.4, 14.2 Hz, 1H), 3.06 (dd, *J* = 7.2, 14.0 Hz, 1H), 1.40 (s, 9H). ^{19}F{^{1}H} NMR (377 MHz, Chloroform-*d*) δ -141.7 – -143.5 (m), -155.4 (t, *J* = 20.8 Hz), -162.3 (td, *J* = 8.1, 21.8 Hz).

Data identical to J. L. Clark, R. M. Neyyappadath, C. Yu, A. M. Z. Slawin, D. B. Cordes, D. O'Hagan, *Chem. Eur. J.*, **2021**, *27*, 16000 – 16005.

(2*R*,5*S*)-2-Isopropyl-3,6-dimethoxy-5-(2,4,6-trifluorobenzyl)-2,5-dihydro-pyrazine (17)

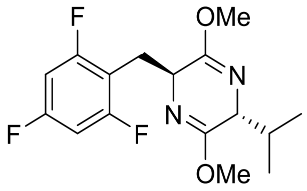

n-Butyllithium (*n*-BuLi, 2.5 M in THF, 2.6 mL, 5.76 mmol) was added slowly to a solution of **15** (888.9 mg, 4.8 mmol) in 8 mL in dry THF at -78 °C. The reaction was stirred at -78 °C for 1.5 h. A cooled solution of 2-(bromomethyl)-1,3,5-trifluorobenzene (**16**, 1.3 g, 5.76 mmol) in THF (3 mL) was then added *via* cannula, and the mixture was stirred at -78 °C for 3.5 h. After quenching with of water (8 mL) at -78 °C, the reaction mixture was partitioned between ethyl acetate and 1 M HCl. The aqueous layer was extracted with ethyl acetate (3×15 mL). The combined organic phase was washed with brine (30 mL) and dried over anhydrous Na_2SO_4, concentrated under reduced pressure, and purified by chromatography (hexane : ethyl

acetate = 50 : 1 and TLC hexane : ethyl acetate = 10 : 1) to give the product as a yellow oil (1.09 g, 69%). ^{1}H NMR (400 MHz, Chloroform-*d*) δ 6.65 – 6.55 (m, 2H), 4.21 (dd, *J* = 5.0, 8.2 Hz, 1H), 3.71 (s, 3H), 3.67 (d, *J* = 3.4 Hz, 1H), 3.59 (s, 3H), 3.26 – 2.83 (m, 2H), 2.20 (m, *J* = 3.5, 6.9, 10.4 Hz, 1H), 1.00 (d, *J* = 6.9 Hz, 3H), 0.63 (d, *J* = 6.8 Hz, 3H). ^{13}C NMR (126 MHz, Chloroform-*d*) δ 164.4, 163.3 (dd, *J* = 11.6, 15.4 Hz), 163.1, 162.7 (t, *J* = 15.5 Hz), 161.3 (dd, *J* = 11.7, 15.3 Hz), 160.8 (t, *J* = 15.5 Hz), 110.8 (td, *J* = 4.7, 20.6 Hz), 102.2 – 99.3 (m), 60.9, 55.6, 52.8 (d, *J* = 21.5 Hz), 31.9, 28.1, 19.4, 16.9. ^{19}F{^{1}H} NMR (377 MHz, Chloroform-*d*) δ -110.5, -110.9 (t, *J* = 5.7 Hz). HRMS (ES^{+}) *m/z*: [M + Na]$^{+}$ calculated for $C_{16}H_{19}F_{3}N_{2}O_{2}Na$ 351.1291; found 351.1286.

Methyl (*S*)-2-((*tert*-butoxycarbonyl)amino)-3-(2,4,6-trifluorophenyl) pro-panoate (19c)

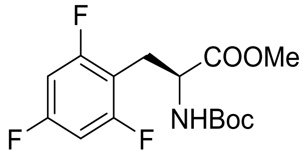

HCl (1 N, 7 mL) was added to a solution of **17** (1 g, 3 mmol) in CH_3CN (6 mL). The reaction was stirred for 18 h and concentrated under reduced pressure. MeOH was added, and the mixture was again concentrated. This was repeated three times, and then the same process was repeated with toluene once. The residue was dissolved in DCM (7 mL), and Et_3N (3 mL, 21 mmol) and di-*tert*-butyl dicarbonate (Boc_2O, 2.5 g, 11.4 mmol) were added. The reaction was stirred for 6 h, diluted with DCM, and washed sequentially with 1 N HCl (10 mL x 2) and brine (10 mL). The organic phase was dried over anhydrous Na_2SO_4, concentrated in under reduced pressure, and purified *via* flash column chromatography (hexane : ethyl acetate = 35 : 1 to 25 : 1 to 15 : 1 and TLC hexane : ethyl acetate = 6 : 1) to give the product as a white crystalline solid (650 mg, 65%). M.p. 63-64 °C. ^{1}H NMR (400 MHz,

Chloroform-*d*) δ 6.70 – 6.58 (m, 2H), 5.08 (d, *J* = 8.5 Hz, 1H), 4.56 (m, *J* = 7.1 Hz, 1H), 3.75 (s, 3H), 3.29 – 2.89 (m, 2H), 1.38 (s, 9H). ^{13}C NMR (126 MHz, Chloroform-*d*) δ 172.0, 162.9 (q, *J* = 13.1, 14.5 Hz), 161.5 – 160.1 (m), 155.0, 108.6 (td, *J* = 4.7, 20.5 Hz), 100.2 (t, *J* = 27.5 Hz), 80.1, 58.7, 52.8 (d, *J* = 29.0 Hz), 52.2, 31.5, 28.4 (d, *J* = 15.6 Hz), 25.7, 19.1, 17.7. ^{19}F{^{1}H} NMR (376 MHz, Chloroform-*d*) δ -109.3 (t, *J* = 6.0 Hz), -111.6 (d, *J* = 6.0 Hz). HRMS (ES^{+}) *m/z*: [M + Na]$^{+}$ calculated for $C_{15}H_{18}F_{3}NO_{4}Na$ 356.1080; found 356.1081.

Rh Catalyst (Rh(CAAC)(COD)Cl) 6

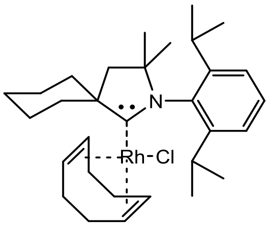

6

The preparation of Rh(CAAC)(COD)Cl **6** followed that reported in the literature[9,10] with minor modification. $[RhCODCl]_2$ (154 mg, 0.32 mmol), 2-(2,6-Diisopropylphenyl)-3,3-dimethyl-2-azaspiro[4.5]dec-1-en-2-ium hydrogen dichloride (262 mg, 0.658 mmol) and KHMDS (300 mg, 1.50 mmol) were added to a Schlenk tube inside an argon-filled glovebox. THF (10 mL) was added dropwise over 10 min at -78 °C. The resulting suspension was stirred for 10 min at -78 °C, and then warmed to room temperature and stirred for 16 h. The mixture was filtered and concentrated under reduced pressure to give the crude product, which was purified *via* flash column chromatography (pentane : Et_2O = 19 : 1). Active fractions were combined and concentrated under reduced pressure to give an oily residue. The residue was redissolved in DCM (1 mL) and **6** was precipitated by dropwise addition of pentane. The excess solvent was decanted giving the catalyst as a yellow solid **6** (183.8 mg, 50%).

Hydrogenation of fluorinated (*S*)-phenylalanines (General Procedure B)

Activated 4 Å molecular sieves (m(ester) x 10) and the selected fluorinated (*S*)-phenylalanine ester (1 eq.) and **6** (1.6 mol%) were suspended in dry hexane in a vial and placed inside an autoclave. The autoclave was pressurised with hydrogen gas to 50 bar and the reaction mixture stirred at r.t. for 3 d. After depressurising and removing the vial, the suspension was filtered and concentrated under reduced pressure to give the product, which was purified by chromatography.

Boc protected (*S*)-cyclohexylalanine methyl ester (20a)

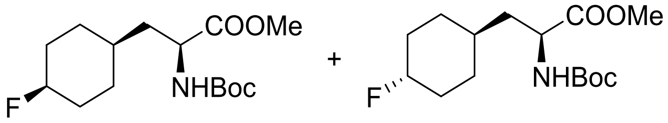

Ester **20a** was prepared according to General Procedure B from 4 Å molecular sieves (3 g), **19a** (297.33 mg, 1 mmol), and **6** (9 mg, 0.016 mmol) in dry hexane (9 mL) and was purified by chromatography (hexane : ethyl acetate = 30 : 1 to 20 : 1 to 10 : 1 and TLC hexane : ethyl acetate = 6 : 1) to give the product as a light yellow oil (218.4 mg, 72%). ^{1}H NMR (400 MHz, Chloroform-*d*) δ 4.90 (d, *J* = 9.0 Hz, 1H), 4.80 (m, *J* = 48.5 Hz, 1H), 4.35 (m, *J* = 6.9 Hz, 1H), 3.73 (s, 3H), 2.05 – 1.92 (m, 2H), 1.68 (m, *J* = 9.1 Hz, 1H), 1.56 – 1.45 (m, 4H), 1.44 (s, 9H), 1.43 – 1.23 (m, 4H). ^{13}C NMR (126 MHz, Chloroform-*d*) δ 174.1, 155.6, 89.6, 88.2, 80.1, 52.4 (d, *J* = 5.5 Hz), 51.5, 40.1, 33.1, 32.5 – 31.5 (m), 30.5 (t, *J* = 20.5 Hz), 29.6, 28.4, 27.3, 26.3. ^{19}F{^{1}H} NMR (376 MHz, Chloroform-*d*) δ -170.0, -184.6. HRMS (ES^{+}) *m/z*: [M + Na]$^{+}$ calculated for $C_{15}H_{26}FNO_4Na$ 326.1727; found 326.1731.

Boc protected (*S*)-cyclohexylalanine methyl ester (20b)

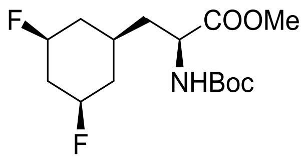

Ester **20b** was prepared according to General Procedure B from 4 Å molecular sieves (7 g), **19b** (787.4 mg, 1 mmol), and **6** (22.8 mg, 0.04 mmol) in dry hexane (15 mL) and was purified by chromatography (hexane : ethyl acetate = 30 : 1 to 20 : 1 to 10 : 1 and TLC hexane : ethyl acetate = 6 : 1) to give the product as a light yellow oil (537.7 mg, 67%). ^{1}H NMR (400 MHz, Chloroform-*d*) δ 4.97 (d, *J* = 8.8 Hz, 1H), 4.59 – 4.30 (m, 2H), 3.74 (s, 3H), 2.61 (m, *J* = 5.5 Hz, 1H), 2.34 – 2.04 (m, 2H), 1.82 (ddt, *J* = 6.5, 13.8 Hz, 1H), 1.65 – 1.56 (m, 2H), 1.48 (m, 1H), 1.43 (s, 9H), 1.24 – 1.06 (m, 2H). ^{13}C NMR (126 MHz, Chloroform-*d*) δ 173.3, 155.5, 89.1 (d, *J* = 15.9 Hz), 87.7 (d, *J* = 15.9 Hz), 80.4, 52.6, 51.5, 39.9 – 39.0 (m), 38.4 (d, *J* = 18.1 Hz), 37.5 (d, *J* = 18.3 Hz), 28.4, 27.4 (t, *J* = 12.0 Hz), 14.3. ^{19}F{^{1}H} NMR (377 MHz, Chloroform-*d*) δ -174.4, -174.5. HRMS (ES^{+}) *m/z*: [M + Na]$^{+}$ calculated for $C_{15}H_{25}F_{2}NO_{4}Na$ 344.1644; found 344.1647.

Boc protected (*S*)-cyclohexylalanine methyl ester (20c)

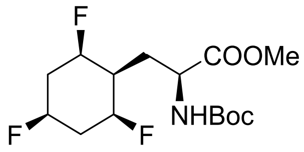

Ester **20c** was prepared according to General Procedure B from 4 Å molecular sieves (6 g), **19c** (600 mg, 1.8 mmol), and **6** (16.5 mg, 0.03 mmol) in dry hexane (10 mL) and was purified by chromatography (hexane : ethyl acetate = 30 : 1 to 10 : 1 to 1 : 1 and TLC hexane : ethyl acetate = 2 : 1) to give the product as a light yellow oil (342 mg, 56%). ^{1}H NMR (400 MHz, Chloroform-*d*) δ 5.18 – 4.62 (m, 3H), 4.43 (m, 1H), 3.74 (s, 3H), 2.36 – 2.01 (m, *J* = 12.6 Hz, 2H), 1.83 (ddt, *J* = 14.7, 27.7, 44.8 Hz, 4H), 1.41 (s, 9H), 1.24 (td, *J* = 3.1, 6.3,

7.1 Hz, 2H). ^{13}C NMR (126 MHz, Chloroform-*d*) δ 173.3, 155.9, 89.0, 87.6, 87.1, 85.7, 85.5, 84.1, 80.4, 52.7, 51.4, 39.1, 35.5 – 33.7 (m), 31.1, 28.4. $^{19}F\{^1H\}$ NMR (377 MHz, Chloroform-*d*) δ -177.9 (t, J = 13.3 Hz), -190.8 (dd, J = 13.3, 18.6 Hz), -192.6 (dd, J = 13.4, 18.5 Hz). HRMS (ES^+) *m/z*: $[M + Na]^+$ calculated for $C_{15}H_{24}F_3NO_4Na$ 362.1550; found 362.1546.

Boc protected (*S*)-cyclohexylalanine methyl ester (20d)

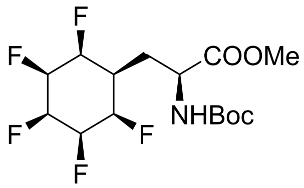

Ester **20d** was prepared according to General Procedure B from 4 Å molecular sieves (5 g), **19d** (499 mg, 1.35 mmol), and **6** (12.4 mg, 0.02 mmol) in dry hexane (10 mL) and was purified by chromatography (hexane : ethyl acetate = 30 : 1 to 10 : 1 to 1 : 1 and TLC hexane : ethyl acetate = 2 : 1) to give the product as a light yellow solid (314.2 mg, 62%). M.p. 172-173 °C. ^1H NMR (400 MHz, Chloroform-*d*) δ 5.50 – 4.22 (m, 6H), 3.79 (s, 3H), 2.56 – 1.98 (m, J = 12.4 Hz, 2H), 1.99 – 1.69 (m, 2H), 1.43 (s, 9H). $^{19}F\{^1H\}$ NMR (377 MHz, Chloroform-*d*) δ -203.4 (dt, J = 11.9, 60.8 Hz), -211.1 (d, J = 28.3 Hz), -212.9 (d, J = 29.2 Hz), -216.8 (tt, J = 11.2, 26.4 Hz). $[\alpha]^{20}_D$ = -23.7° (c = 0.1, MeOH).

Fmoc protection of fluorinated amino acids (General Procedure C)

The hydrogenated cyclohexane products (1 eq.) were dissolved in a mixture of 95% TFA, 2.5% water and 2.5% triethylsilane, and stirred at r.t. for 1 h. The mixture was then concentrated under reduced pressure, and the residue was treated with a mixture of 10% aq solution of Na_2CO_3 aq. (40%), water (25%) and acetonitrile (35%) at pH~10, followed by an addition of Fmoc-ONSu (1 eq.).

The resulting solution was stirred at r.t. for 2.5 h, then poured into 2 N HCl solution, and extracted with DCM (3 × 15 ml). The combined organic layers were washed with a 10:1 mixture of brine and 1N HCl and dried over anhydrous Na_2SO_4. The mixture was concentrated under reduced pressure and the product was purified by chromatography.

Fmoc protected (*S*)-cyclohexylalanine methyl ester (21a)

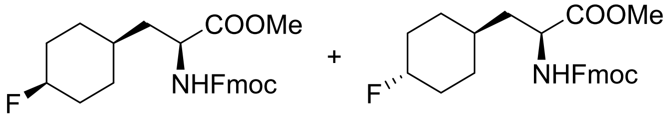

Ester **21a** was prepared according to General Procedure C from **20a** (364 mg, 1.2 mmol) in 11 mL TFA, water and triethylsilane mixture; Fmoc-ONSu (404.8 mg, 1.2 mmol) in 26 mL Na_2CO_3 solution, water and acetonitrile mixture and was purified by chromatography (hexane : ethyl acetate = 30 : 1 to 20 : 1 to 10 : 1 and TLC hexane : ethyl acetate = 4 : 1) to give the product as a white solid (377.8 mg, 74%). M.p. 51-53 °C. ^{1}H NMR (400 MHz, Chloroform-*d*) δ 7.77 (d, *J* = 7.6 Hz, 2H), 7.60 (t, *J* = 6.9 Hz, 2H), 7.41 (t, *J* = 7.6 Hz, 2H), 7.32 (td, *J* = 1.4, 7.4 Hz, 2H), 5.18 (d, *J* = 8.8 Hz, 1H), 4.81 (m, *J* = 48.2 Hz, 1H), 4.52 – 4.34 (m, 2H), 4.23 (m, *J* = 7.0 Hz, 1H), 3.75 (s, 3H), 2.00 (m, 2H), 1.79 – 1.64 (m, 1H), 1.56 – 1.28 (m, 8H). ^{13}C NMR (126 MHz, Chloroform-*d*) δ 173.7, 156.1, 144.0 (d, *J* = 23.6 Hz), 141.5, 127.9, 127.2, 125.2 (d, *J* = 7.2 Hz), 120.2, 89.5, 88.2, 67.1, 52.6, 51.9, 47.3, 40.0, 33.0, 32.2, 30.5 (t, *J* = 20.8 Hz), 27.3, 26.2. ^{19}F{^{1}H} NMR (377 MHz, Chloroform-*d*) δ -170.2, -184.7. HRMS (ES$^+$) *m/z*: [M + Na]$^+$ calculated for $C_{25}H_{28}FNO_4Na$ 448.1895; found 448.1892.

Fmoc protected (*S*)-cyclohexylalanine methyl ester (21b)

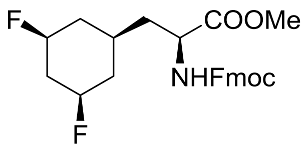

Ester **21b** was prepared according to General Procedure C from **20b** (385.6 mg, 1.2 mmol) in 11 mL TFA, water and triethylsilane mixture; Fmoc-ONSu (404.8 mg, 1.2 mmol) in 26 mL Na_2CO_3 solution, water and acetonitrile mixture and was purified *via* flash column chromatography (hexane : ethyl acetate = 30 : 1 to 20 : 1 to 10 : 1 and TLC hexane : ethyl acetate = 4 : 1) to give the product as a white solid (351.2 mg, 66%). M.p. 59-60 °C. ^{1}H NMR (400 MHz, Chloroform-*d*) δ 7.77 (d, *J* = 7.6 Hz, 2H), 7.59 (t, *J* = 6.3 Hz, 2H), 7.41 (t, *J* = 7.5 Hz, 2H), 7.32 (td, *J* = 1.4, 7.4 Hz, 2H), 5.25 (d, *J* = 8.7 Hz, 1H), 4.63 – 4.31 (m, 4H), 4.22 (m, *J* = 6.7 Hz, 1H), 3.76 (s, 3H), 2.61 (m, 1H), 2.34 – 2.05 (m, 2H), 1.86 (ddd, *J* = 4.9, 8.4, 13.6 Hz, 1H), 1.73 – 1.56 (m, 2H), 1.41 (t, *J* = 7.1 Hz, 1H), 1.22 – 1.07 (m, 2H). ^{13}C NMR (126 MHz, Chloroform-*d*) δ 173.0, 156.1, 143.9 (d, *J* = 27.3 Hz), 141.5, 127.9, 127.2, 125.1, 120.2 (d, *J* = 2.9 Hz), 89.0 (d, *J* = 19.0 Hz), 87.6 (d, *J* = 18.1 Hz), 67.1, 52.8, 51.9, 47.3, 40.3 – 38.6 (m), 38.4 (d, *J* = 18.7 Hz), 37.4 (d, *J* = 18.3 Hz), 27.3. ^{19}F{^{1}H} NMR (377 MHz, Chloroform-*d*) δ -174.4 (d, *J* = 3.0 Hz), -174.6 (d, *J* = 2.9 Hz). HRMS (ES$^+$) *m/z*: $[M + Na]^+$ calculated for $C_{25}H_{27}F_2NO_4Na$ 466.1800; found 466.1795.

Fmoc protected (*S*)-cyclohexylalanine methyl ester (21c)

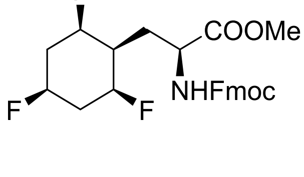

Ester **21c** was prepared according to General Procedure C from **20c** (441.1 mg, 1.3 mmol) in 12 mL TFA, water and triethylsilane mixture; Fmoc-ONSu (438.6 mg, 1.3 mmol) in 28 mL Na_2CO_3 solution, water and acetonitrile mixture and was purified by chromatography (hexane : ethyl acetate = 30 : 1 to 20 : 1

to 10 : 1 and TLC hexane : ethyl acetate = 2 : 1) to give the product as a white solid (378 mg, 63%). M.p. 65-67 °C. ^{1}H NMR (400 MHz, Chloroform-*d*) δ 7.77 (d, *J* = 7.5 Hz, 2H), 7.59 (t, *J* = 8.3 Hz, 2H), 7.41 (t, *J* = 7.5 Hz, 2H), 7.31 (td, *J* = 1.2, 7.4 Hz, 2H), 5.37 (d, *J* = 8.3 Hz, 1H), 4.97 (m, *J* = 39.3 Hz, 1H), 4.81 (m, 1H), 4.70 (d, *J* = 47.3 Hz, 1H), 4.48 – 4.33 (m, 2H), 4.21 (t, *J* = 6.8 Hz, 1H), 3.78 (s, 3H), 2.51 (d, *J* = 46.8 Hz, 2H), 1.98 – 1.68 (m, 4H), 1.27 (d, *J* = 11.5 Hz, 2H). ^{13}C NMR (126 MHz, Chloroform-*d*) δ 173.1, 156.3, 143.9 (d, *J* = 27.3 Hz), 141.5, 127.9, 127.2, 120.2, 89.1 (d, *J* = 19.5 Hz), 87.6 (d, *J* = 18.1 Hz), 67.2, 52.9, 51.8, 47.3. ^{19}F{^{1}H} NMR (377 MHz, Chloroform-*d*) δ -178.1 (t, *J* = 13.2 Hz), -190.6 (dd, *J* = 13.2, 17.4 Hz), -192.4 (dd, *J* = 13.0, 17.5 Hz). HRMS (ES$^+$) *m/z*: $[M + Na]^+$ calculated for $C_{25}H_{26}F_3NO_4Na$ 484.1706; found 484.1705.

Fmoc protected (*S*)-cyclohexylalanine methyl ester (21d)

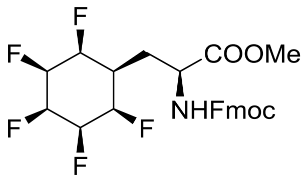

Ester **21d** was prepared according to General Procedure C from **20d** (320 mg, 0.85 mmol) in 8 mL TFA, water and triethylsilane mixture; Fmoc-ONSu (287.6 mg, 0.85 mmol) in 14.5 mL Na_2CO_3 solution, water and acetonitrile mixture and was purified *via* flash column chromatography (hexane : ethyl acetate = 20 : 1 to 10 : 1 to 1 : 1 and TLC hexane : ethyl acetate = 1 : 1) to give the product as a white solid (253.7 mg, 60%). ^{1}H NMR (400 MHz, Acetone-d_6) δ 7.87 (d, *J* = 7.6 Hz, 2H), 7.69 (t, *J* = 7.5 Hz, 2H), 7.42 (t, *J* = 7.5 Hz, 2H), 7.33 (td, *J* = 1.2, 7.4 Hz, 2H), 5.56 – 4.78 (m, 5H), 4.53 – 4.15 (m, 4H), 3.73 (s, 3H), 2.88 – 2.73 (m, 2H), 2.51 (ddd, *J* = 4.5, 9.3, 14.0 Hz, 1H), 2.37 – 2.12 (m, 1H). ^{19}F{^{1}H} NMR (376 MHz, Acetone-d_6) δ -205.1 (dt, *J* = 11.5, 21.0 Hz), -211.7 (ddd, *J* = 11.4, 19.6, 29.6 Hz), -213.3 (ddd, *J* = 11.1, 18.9, 30.2 Hz), -217.5 (tt,

J = 10.9, 25.1 Hz). HRMS (ES$^+$) *m/z*: $[M + Na]^+$ calculated for $C_{25}H_{24}F_5NO_4Na$ 520.1518; found 520.1514.

Synthesis of Fmoc protected fluorinated amino acids (General Procedure D)

The Fmoc protected products (1 eq.) were dissolved in THF. Lithium hydroxide (LiOH) (1.95 eq.) dissolved in ice-cold water was added to the reaction and stirred at 0 °C for 30 min. A further 1 eq. of LiOH in water was added and the solution stirred for a further 2 h. The reaction was quenched with ice-cold HCl solution (0.1 M, 20 mL) and extracted into ethyl acetate (3 x 15 mL). The organic layers were combined, dried over anhydrous Na_2SO_4, filtered and concentrated. The product was then purified by chromatography.

Fmoc protected (*S*)-cyclohexylalanine (22a)

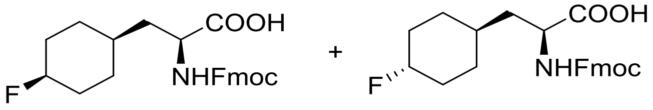

Carboxylic acid **22a** was prepared according to General Procedure D from **21a** (310.6 mg, 0.73 mmol), $LiOH{\cdot}H_2O$ (59.7 mg and 30.6 mg, 1.42 mmol and 0.73 mmol) in THF (3 mL) and water (3 mL) and was purified by chromatography (hexane : ethyl acetate = 20 : 1 and then DCM : MeOH = 100 : 0 to 50 : 1 to 10 : 1 and TLC DCM : MeOH = 12 : 1) to give the product as a white solid (171.2 mg, 57%). M.p. 76-77 °C. ^{1}H NMR (500 MHz, Chloroform-*d*) δ 7.75 (d, *J* = 7.6 Hz, 2H), 7.57 (t, *J* = 10.6 Hz, 2H), 7.38 (t, *J* = 7.5 Hz, 2H), 7.32 – 7.26 (m, 2H), 5.40 (s, 1H), 4.79 (d, *J* = 48.5 Hz, 1H), 4.39 (m, 3H), 4.20 (dd, *J* = 7.0 Hz, 1H), 2.12 – 1.26 (m, 13H). ^{13}C NMR (126 MHz, Chloroform-*d*) δ 156.4, 143.8 (d, *J* = 24.3 Hz), 141.4, 127.9, 127.2, 125.2 (d, *J* = 6.9 Hz), 124.8, 120.2, 92.9, 88.8 (d, *J* = 167.5 Hz), 68.1, 67.2, 47.2, 39.5, 33.0 (d, *J* = 22.7 Hz), 32.2 (t, *J* = 20.1 Hz),

30.4 (t, J = 20.9 Hz), 29.7 (d, J = 40.2 Hz), 27.3, 26.1, 25.7, 18.5 (d, J = 1077.7 Hz). $^{19}F\{^1H\}$ NMR (470 MHz, Chloroform-*d*) δ -170.1, -184.5. HRMS (ES$^+$) *m/z*: $[M + Na]^+$ calculated for $C_{24}H_{26}FNO_4Na$ 434.1738; found 434.1733.

Fmoc protected (*S*)-cyclohexylalanine (22b)

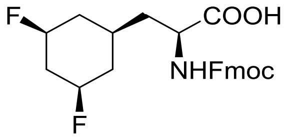

Carboxylic acid **22b** was prepared according to General Procedure D from **21b** (250 mg, 0.56 mmol), LiOH·H_2O (46.2 mg and 23.6 mg, 1.42 mmol and 0.56 mmol) in 3 mL THF and 4 mL water and was purified by chromatography (hexane : ethyl acetate = 20 : 1 and then DCM : MeOH = 100 : 0 to 50 : 1 to 10 : 1 and TLC DCM : MeOH = 8 : 1) to give the product as a white solid (133 mg, 55%). M.p. 82-83 °C. ^1H NMR (400 MHz, Chloroform-*d*) δ 7.75 (d, J = 7.6 Hz, 2H), 7.55 (t, J = 9.3 Hz, 2H), 7.42 – 7.33 (m, 2H), 7.33 – 7.26 (m, 2H), 5.39 (s, 1H), 4.28 (d, J = 82.0 Hz, 6H), 2.65 – 1.30 (m, 9H). $^{19}F\{^1H\}$ NMR (377 MHz, Chloroform-*d*) δ -174.3, -174.4.

Fmoc protected (*S*)-cyclohexylalanine (22c)

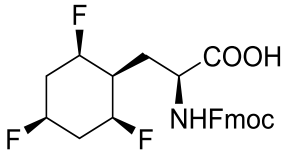

Carboxylic acid **22c** was prepared according to General Procedure D from **21c** (140 mg, 0.3 mmol), LiOH·H_2O (24.5 mg and 12.6 mg, 0.58 mmol and 0.3 mmol) in 2 mL THF and 2 mL water and was purified by chromatography (hexane : ethyl acetate = 20 : 1 and then DCM : MeOH = 100 : 0 to 50 : 1 to 10 : 1 and TLC DCM : MeOH = 12 : 1) to give the product as a white solid (65 mg,

48%). M.p. 88-89 °C. ^{1}H NMR (400 MHz, Chloroform-*d*) δ 7.76 (d, *J* = 7.3 Hz, 2H), 7.58 (t, *J* = 8.3 Hz, 2H), 7.52 (t, *J* = 7.3 Hz, 2H), 7.22 (td, *J* = 3.2, 7.6 Hz, 2H), 5.36 (d, *J* = 8.3 Hz, 1H), 4.87 (d, *J* = 39.3 Hz, 1H), 4.82 (m, 1H), 4.70 (d, *J* = 47.4 Hz, 1H), 4.46 – 4.30 (m, 2H), 4.22 (t, *J* = 6.9 Hz, 1H), 2.53 (d, *J* = 46.6 Hz, 2H), 1.99 – 1.68 (m, 4H), 1.25 (d, *J* = 11.3 Hz, 2H). ^{19}F{^{1}H} NMR (376 MHz, Chloroform-*d*) δ -178.2, -190.4, -192.0. HRMS (ES$^+$) *m/z*: [M + Na]$^+$ calculated for $C_{24}H_{24}F_3NO_4Na$ 470.1550; found 470.1548.

Fmoc protected (*S*)-cyclohexylalanine (22d)

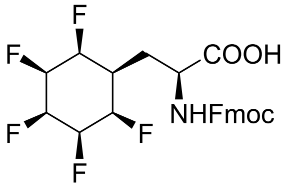

Carboxylic acid **22d** was prepared according to General Procedure D from **21d** (597 mg, 1.2 mmol), LiOH·H_2O (98.2 mg and 50 mg, 2.34 mmol and 1.2 mmol) in 4 mL THF and 4 mL water and was purified by chromatography (hexane : ethyl acetate = 20 : 1 and then DCM : MeOH = 100 : 0 to 50 : 1 to 10 : 1 and TLC DCM : MeOH = 8 : 1) to give the product as a white solid (249.4 mg, 43%). ^{1}H NMR (400 MHz, Acetone-d_6) δ 7.86 (dt, *J* = 1.2, 7.8 Hz, 2H), 7.69 (d, *J* = 7.6 Hz, 2H), 7.34 (tdd, *J* = 0.6, 1.2, 7.5 Hz, 2H), 7.32 (tdd, *J* = 1.5, 2.6, 7.3 Hz, 2H), 5.62 – 4.75 (m, 5H), 4.52 – 4.15 (m, 4H), 2.83 – 2.68 (m, 2H), 2.51 (ddd, *J* = 4.5, 9.6, 14.0 Hz, 1H), 2.22 (m, 1H). ^{19}F{^{1}H} NMR (376 MHz, Acetone-d_6) δ -205.0, -211.8, -213.3, -217.5. $[\alpha]^{20}_D$ = -14.67° (c = 0.1, MeOH). HRMS (ES$^+$) *m/z*: [M + Na]$^+$ calculated for $C_{24}H_{22}F_5NO_4Na$ 506.1361; found 506.1363.

Synthesis of Boc protected fluorinated amino acids (General Procedure E)

Lithium hydroxide (2 eq.) dissolved in ice-cold water was added to a solution of the Boc protected product (1 eq.) in MeOH and the reaction was stirred at r.t. for 3 h. The reaction was quenched with ice-cold HCl (0.1 M, 20 mL) and extracted into ethyl acetate (3 x 15 mL). The organic layers were combined, dried over anhydrous Na_2SO_4, filtered and concentrated under reduced pressure to give the crude product. It was then purified *via* flash column chromatography.

Boc protected (*S*)-cyclohexylalanine (26a)

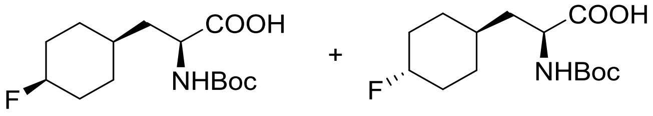

26a was prepared according to General Procedure E from **20a** (200.2 mg, 0.66 mmol), $LiOH·H_2O$ (55.4 mg, 1.32 mmol) in 3.3 mL MeOH and 3.3 mL water and was purified *via* flash column chromatography (hexane : ethyl acetate = 20 : 1 and then DCM : MeOH = 100 : 0 to 50 : 1 to 10 : 1 and TLC DCM : MeOH = 12 : 1) to give the product as a white solid (93.5 mg, 49%). M.p. 55-56 °C. ^{1}H NMR (500 MHz, Chloroform-*d*) δ 4.92 (d, *J* = 8.8 Hz, 1H), 4.79 (m,1H), 4.35 (dd, *J* = 7.0 Hz, 1H), 2.05 – 1.92 (m, 2H), 1.65 (d, *J* = 9.1 Hz, 1H), 1.60 – 1.45 (m, 4H), 1.44 – 1.23 (m, 13H). ^{13}C NMR (126 MHz, Chloroform-*d*) δ 177.5 (d, *J* = 46.8 Hz), 156.8, 155.7 (d, *J* = 15.8 Hz), 92.3 (d, *J* = 171.7 Hz), 89.5, 88.2, 81.8, 80.2 (d, *J* = 14.7 Hz), 52.5 (d, *J* = 13.6 Hz), 51.5 (d, *J* = 6.9 Hz), 39.7, 32.9 (d, *J* = 37.6 Hz), 32.2 (t, *J* = 19.5 Hz), 30.4 (t, *J* = 19.9 Hz), 29.6 (d, *J* = 41.5 Hz), 28.4 (d, *J* = 6.8 Hz), 27.2 (d, *J* = 8.5 Hz), 26.2. ^{19}F{^{1}H} NMR (376 MHz, Chloroform-*d*) δ -170.0, -184.6.

Boc protected (*S*)-cyclohexylalanine (26b)

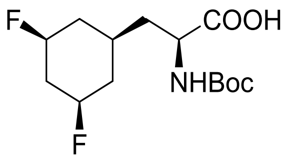

Carboxylic acid **26b** was prepared according to General Procedure E from **20b** (560 mg, 1.74 mmol), LiOH·H_2O (146.2 mg, 3.49 mmol) in 7 mL MeOH and 7 mL water and was purified *via* flash column chromatography (hexane : ethyl acetate = 20 : 1 and then DCM : MeOH = 100 : 0 to 50 : 1 to 10 : 1 and TLC DCM : MeOH = 12 : 1) to give the product as a white solid (321.4 mg, 60%). M.p. 60-61 °C. ^{1}H NMR (500 MHz, Chloroform-*d*) δ 5.01 (d, *J* = 8.9 Hz, 1H), 4.46 (dt, *J* = 4.8, 52.5 Hz, 2H), 2.62 (d, *J* = 9.2 Hz, 1H), 2.32 – 2.09 (m, 2H), 1.88 (dd, *J* = 11.7, 15.7 Hz, 1H), 1.64 (dt, *J* = 10.6, 21.2 Hz, 3H), 1.45 (d, *J* = 5.3 Hz, 9H), 1.27 – 1.06 (m, 3H). ^{13}C NMR (126 MHz, Chloroform-*d*) δ 177.3, 176.5, 157.0, 155.8, 89.1 (d, *J* = 16.0 Hz), 87.7 (d, *J* = 16.1 Hz), 82.5, 80.7, 59.7 (d, *J* = 249.1 Hz), 53.6, 52.4, 51.4, 40.1 – 38.7 (m), 38.3 (d, *J* = 17.6 Hz), 37.7 (dd, *J* = 18.0, 35.5 Hz), 28.4, 27.8 – 26.7 (m). ^{19}F{^{1}H} NMR (377 MHz, Chloroform-*d*) δ -174.3 – -174.7 (m). HRMS (ES^{+}) *m/z*: [M + Na]$^{+}$ calculated for $C_{14}H_{23}F_2NO_4Na$ 330.1487; found 330.1485.

Boc protected (*S*)-cyclohexylalanine (26c)

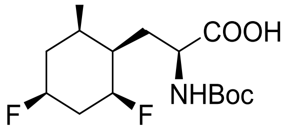

Carboxylic acid **26c** was prepared according to General Procedure E from **20c** (605.4 mg, 1.78 mmol), LiOH·H_2O (150 mg, 3.57 mmol) in 7 mL MeOH and 7 mL water and was purified *via* flash column chromatography (hexane : ethyl acetate = 20 : 1 and then DCM : MeOH = 100 : 0 to 50 : 1 to 10 : 1 and TLC DCM : MeOH = 12 : 1) to give the product as a white solid (500 mg, 86%). M.p. 69-71 °C. ^{1}H NMR (500 MHz, Methanol-d_4) δ 4.90 (d, *J* = 3.6 Hz, 1H), 4.80 (m,

1H), 4.80 – 4.67 (m, 1H), 4.25 (dd, *J* = 4.5, 10.8 Hz, 2H), 2.49 (qdd, *J* = 2.9, 11.9, 15.1 Hz, 2H), 2.34 (ddd, *J* = 4.8, 9.3, 13.8 Hz, 2H), 2.07 – 1.76 (m, 4H), 1.44 (s, 9H). ^{13}C NMR (126 MHz, Methanol-d_4) δ 175.0, 156.9, 89.3, 87.9, 87.1, 85.8 (d, *J* = 15.5 Hz), 84.5, 79.2, 51.1, 38.6 (t, *J* = 19.4 Hz), 33.7 (q, *J* = 21.0 Hz), 29.3, 27.3. ^{19}F{^{1}H} NMR (377 MHz, Methanol-d_4) δ -178.1 (t, *J* = 13.5 Hz), -191.6 (dd, *J* = 13.7, 19.0 Hz), -193.4 (dd, *J* = 13.6, 19.0 Hz). HRMS (ES$^+$) *m/z*: [M + Na]$^+$ calculated for $C_{14}H_{22}F_3NO_4Na$ 348.1393; found 348.1391.

Boc protected (*S*)-cyclohexylalanine (26d)

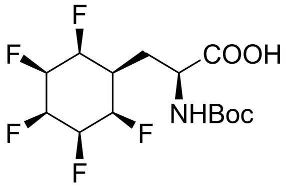

Carboxylic acid **26d** was prepared according to General Procedure E from **20d** (293 mg, 0.78 mmol), LiOH·H_2O (65.5 mg, 1.56 mmol) in 5 mL MeOH and 5 mL water and was purified *via* flash column chromatography (hexane : ethyl acetate = 20 : 1 and then DCM : MeOH = 100 : 0 to 50 : 1 to 10 : 1 and TLC DCM : MeOH = 12 : 1) to give the product as a white solid (211.4 mg, 75%). ^{1}H NMR (500 MHz, Methanol-d_4) δ 5.33 – 4.55 (m, 6H), 4.26 (dd, *J* = 4.5, 10.6 Hz, 1H), 2.46 (d, *J* = 12.4 Hz, 1H), 1.99 – 1.69 (m, 2H), 1.43 (s, 9H). ^{19}F{^{1}H} NMR (377 MHz, Methanol-d_4) δ -206.1 (dt, *J* = 13.2, 23.3 Hz), -212.4 (dq, *J* = 13.1, 14.0, 26.3 Hz), -213.7 – -214.3 (m), -218.2 (dq, *J* = 12.0, 13.1, 29.2 Hz). $[\alpha]^{20}_D$ = -14.23° (c = 0.1, MeOH).

Synthesis of Weinreb amides (General Procedure F)

1-Ethyl-3-(3-dimethylaminopropyl) carbodiimide (EDC, 2 eq.), 1-hydroxybenzotriazole (HOBt, 2 eq.) and triethylamine (4 eq.) were added to

a solution of Boc-protected cyclohexyl amino acids (1 eq.) in DCM. The reaction was then stirred for 20 min after which time N,O-dimethyl hydroxylamine (4 eq.) was added and the system was stirred for 16 h. The mixture was then diluted with DCM (10 mL), washed successively with sodium bicarbonate (1.0 M aq., 10 mL) and potassium hydrogen sulfate solution (1.0 M aq., 10 mL) then dried over anhydrous Na_2SO_4, filtered and concentrated under reduced pressure. The product was then purified by chromatography.

Boc protected (S)-cyclohexylalanine amide (27a)

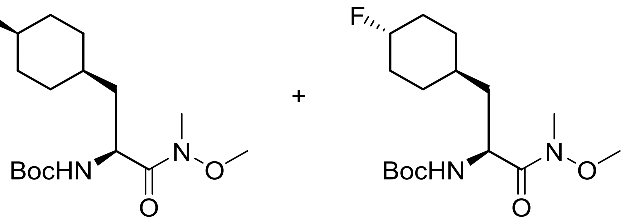

Amide **27a** was prepared according to General Procedure F from **26a** (774 mg, 2.68 mmol), EDC (830.5 mg, 5.35 mmol), HOBt (867.3 mg, 5.35 mmol), Et_3N (0.15 mL, 1.44 mmol), and N,O-dimethyl hydroxylamine (1 g, 10.72 mmol) in 8 mL DCM and was purified *via* flash column chromatography (hexane : ethyl acetate = 20 : 1 to 10 : 1 to 2 : 1 and TLC hexane : ethyl acetate = 3 : 1) to give the product as a white solid (436.5 mg, 49%). M.p. 136-137 °C. ^{1}H NMR (400 MHz, Chloroform-*d*) δ 4.97 (d, *J* = 82.2 Hz, 2H), 4.74 (m, 1H), 3.78 (s, 3H), 3.20 (s, 3H), 2.00 (d, *J* = 16.2 Hz, 2H), 1.78 (d, *J* = 12.1 Hz, 1H), 1.65 – 1.48 (m, 4H), 1.43 (d, *J* = 1.3 Hz, 9H), 1.40 – 1.29 (m, 4H). ^{13}C NMR (126 MHz, Methanol-d_4) δ 158.0, 93.7, 92.4, 90.5, 89.1, 80.4, 62.1, 50.3, 50.0, 39.5, 38.9, 34.2 (d, *J* = 9.0 Hz), 33.4 (dd, *J* = 18.6, 28.1 Hz), 32.5, 31.9, 31.4 (dd, *J* = 21.2, 26.1 Hz), 30.1 (d, *J* = 11.6 Hz), 28.7 (d, *J* = 3.2 Hz), 26.9. ^{19}F{^{1}H} NMR (376 MHz, Chloroform-*d*) δ -170.0, -184.2.

Boc protected (*S*)-cyclohexylalanine amide (27b)

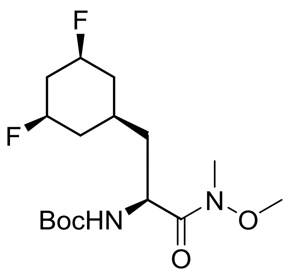

Amide **27b** was prepared according to General Procedure F from **26b** (750 mg, 2.44 mmol), EDC (757.6 mg, 4.88 mmol), HOBt (791.1 mg, 4.88 mmol), Et_3N (1 mL, 9.76 mmol), and N,O-dimethyl hydroxylamine (952 mg, 9.76 mmol) in 6 mL DCM and was purified *via* flash by column chromatography (hexane : ethyl acetate = 20 : 1 to 10 : 1 to 1 : 1 and TLC hexane : ethyl acetate = 3 : 1) to give the product as a white solid (427.5 mg, 50%). M.p. 142-144 °C. ^{1}H NMR (400 MHz, Methanol-d_4) δ 4.67 (d, *J* = 9.3 Hz, 1H), 4.62 – 4.38 (m, 2H), 3.83 (s, 3H), 3.20 (s, 3H), 2.54 (t, *J* = 5.4 Hz, 1H), 2.31 – 2.05 (m, 2H), 1.64 – 1.48 (m, 4H), 1.44 (s, 9H), 1.43 – 1.30 (m, 3H). ^{13}C NMR (126 MHz, Methanol-d_4) δ 175.0, 172.9, 157.9, 90.2 (dd, *J* = 16.1, 18.6 Hz), 88.8 (dd, *J* = 16.0, 18.5 Hz), 80.5, 62.1, 61.5, 50.0, 40.6 (t, *J* = 19.7 Hz), 39.7, 38.8, 38.2 (d, *J* = 18.0 Hz), 32.6 (d, *J* = 31.3 Hz), 28.7, 28.5 (t, *J* = 12.1 Hz), 23.7, 20.9, 14.5 (d, *J* = 4.5 Hz). ^{19}F{^{1}H} NMR (377 MHz, Methanol-d_4) δ -175.6 (dt, *J* = 8.6, 18.3 Hz). HRMS (ES$^+$) *m/z*: [M + Na]$^+$ calculated for $C_{16}H_{28}F_2N_2O_4Na$ 373.1909; found 373.1907.

Boc protected (*S*)-cyclohexylalanine amide (27c)

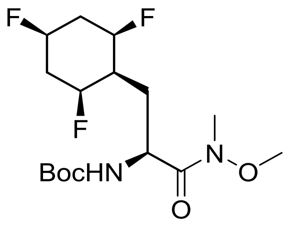

Amide **27c** was prepared according to General Procedure F from **26c** (950.9 mg, 2.92 mmol), EDC (907.5 mg, 5.85 mmol), HOBt (948.4 mg, 5.85 mmol),

Et_3N (1.2 mL, 11.68 mmol), and N,O-dimethyl hydroxylamine (1.1 g, 11.68 mmol) in 7 mL DCM and was purified *via* flash column chromatography (hexane : ethyl acetate = 20 : 1 to 10 : 1 to 1 : 1 and TLC hexane : ethyl acetate = 1 : 1) to give the product as a white solid (387.2 mg, 36%). M.p. 148-149 °C. ^{1}H NMR (400 MHz, Methanol-d_4) δ 4.99 – 4.60 (m, 4H), 3.82 (s, 3H), 3.22 (s, 3H), 2.48 (dddt, *J* = 6.1, 12.3, 15.7, 24.5 Hz, 2H), 2.24 (ddd, *J* = 3.6, 9.9, 13.8 Hz, 1H), 2.09 (d, *J* = 14.7 Hz, 2H), 2.00 – 1.78 (m, 2H), 1.43 (s, 9H). ^{13}C NMR (126 MHz, Methanol-d_4) δ 175.0, 172.8, 157.9, 90.9, 89.5, 88.3, 87.0 (d, *J* = 32.5 Hz), 85.7, 80.5, 62.1, 61.4, 54.8, 50.8, 39.7 (t, *J* = 19.2 Hz), 35.1 (dt, *J* = 20.5, 25.5 Hz), 32.6 (d, *J* = 20.5 Hz), 30.3, 28.7, 23.6, 20.9, 14.5. ^{19}F{^{1}H} NMR (377 MHz, Methanol-d_4) δ -178.0 (t, *J* = 13.3 Hz), -191.2 (t, *J* = 16.0 Hz), -193.0 (dd, *J* = 13.2, 18.7 Hz).

Boc protected (S)-cyclohexylalanine amide (27d)

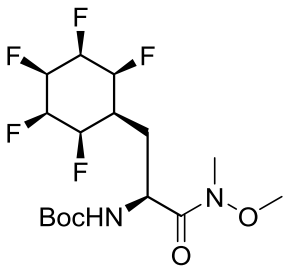

Amide **27d** was prepared according to General Procedure F from **26d** (681 mg, 1.89 mmol), EDC (585.3 mg, 3.77 mmol), HOBt (611.2 mg,3.77 mmol), Et_3N (0.8 mL, 7.54 mmol), and N,O-dimethyl hydroxylamine (735.5 mg, 7.54 mmol) in 5 mL DCM and was purified *via* flash column chromatography (hexane : ethyl acetate = 20 : 1 to 10 : 1 to 1 : 1 and TLC hexane : ethyl acetate = 1 : 1) to give the product as a white solid (495.4 mg, 65%). M.p. 162-164 °C. ^{1}H NMR (400 MHz, Methanol-d_4) δ 5.43 – 4.52 (m, 6H), 4.29 (td, *J* = 6.0, 11.9, 13.6 Hz, 1H), 3.75 (s, 3H), 3.35 (s, 3H), 2.41 (dd, *J* = 6.3, 10.7 Hz, 1H), 1.93 (d, *J* = 17.6 Hz, 2H), 1.44 (s, 9H). ^{19}F{^{1}H} NMR (377 MHz, Methanol-d_4) δ -205.7 – -206.7

(m), -212.4 (d, J = 27.0 Hz), -214.0 (d, J = 25.6 Hz), -218.2 (td, J = 13.5, 28.3, 29.0 Hz). HRMS (ES$^+$) *m/z*: [M + Na]$^+$ calculated for $C_{16}H_{25}F_5N_2O_4Na$ 427.1627; found 427.1625.

Synthesis of carbamate dipeptides (General Procedure G)

TFA (3 eq.) was added to a solution of the carbamate (1 eq.) in DCM and the mixture was stirred at r.t. for 16 h. The mixture was concentrated under reduced pressure to give the crude. A solution of Boc-D-tryptophan (1.5 eq.), EDC (3 eq.), HOBt (3 eq.) and Et_3N (6 eq.) in DCM was stirred at r.t. for 30 min. A solution of the above in DCM was then added and the mixture was stirred for 16 h. The mixture was then diluted with DCM (10 mL), washed successively with sodium bicarbonate (1.0 M aq., 10 mL) and potassium hydrogen sulfate solution (1.0 M aq., 10 mL) then dried over anhydrous Na_2SO_4, filtered and concentrated under reduced pressure to give the crude product. It was then purified *via* flash column chromatography.

Boc protected dipeptide (28a)

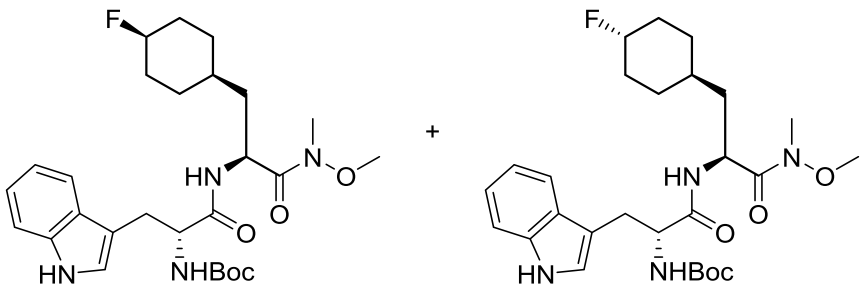

Dipeptide **28a** was prepared according to General Procedure G from **27a** (524.8 mg, 1.58 mmol), TFA (0.4 mL, 4.74 mmol) in 5 mL DCM, and Boc-D-tryptophan (723.3 mg, 2.37 mmol), EDC (735.8 mg, 4.74 mmol), and HOBt (768.4 mg, 4.74 mmol), Et_3N (1 mL, 9.48 mmol) in 3 mL x 2 DCM and was purified *via* flash column chromatography (DCM : MeOH = 100 : 0 to 50 : 1

to 10 : 1 and TLC DCM : MeOH = 12 : 1) to give the product as a white solid (360.5 mg, 44%). M.p. 100-101 °C. ^{1}H NMR (400 MHz, Chloroform-*d*) δ 8.37 (m, 1H), 7.72 – 7.57 (m, 1H), 7.37 – 7.30 (m, 1H), 7.17 (ddd, *J* = 1.3, 7.0, 8.2 Hz, 1H), 7.09 (ddt, *J* = 1.5, 7.0, 8.0 Hz, 1H), 7.05 (d, *J* = 2.3 Hz, 1H), 6.50 (t, *J* = 11.4 Hz, 1H), 5.16 – 4.86 (m, 2H), 4.75 (d, *J* = 48.5 Hz, 1H), 4.48 (d, *J* = 25.2 Hz, 1H), 3.76 (s, 3H), 3.24 (d, *J* = 6.4 Hz, 2H), 3.13 (s, 3H), 1.93 (d, *J* = 13.1 Hz, 2H), 1.62 (d, *J* = 28.8 Hz, 1H), 1.44 (d, *J* = 13.2 Hz, 4H), 1.38 (s, 9H), 1.31 – 1.15 (m, 4H). ^{13}C NMR (126 MHz, Chloroform-*d*) δ 174.9, 172.9 (d, *J* = 32.3 Hz), 172.3, 155.6, 136.3 (d, *J* = 6.3 Hz), 127.9 (d, *J* = 40.5 Hz), 123.2 (d, *J* = 15.7 Hz), 122.1 (d, *J* = 11.7 Hz), 119.6 (d, *J* = 6.5 Hz), 118.8 (d, *J* = 25.5 Hz), 111.3, 110.2 (d, *J* = 16.1 Hz), 92.4 (d, *J* = 171.6 Hz), 89.1 (d, *J* = 167.2 Hz), 80.1 (d, *J* = 16.4 Hz), 61.7, 55.3, 54.4, 53.5, 47.0 (d, *J* = 34.5 Hz), 38.9 (d, *J* = 82.9 Hz), 32.9 – 31.7 (m), 30.2 (dd, *J* = 20.8, 25.7 Hz), 28.4 (d, *J* = 19.5 Hz), 27.7 (d, *J* = 37.6 Hz), 25.8. ^{19}F{^{1}H} NMR (377 MHz, Chloroform-*d*) δ -170.1, -184.1.

Boc protected dipeptide (28b)

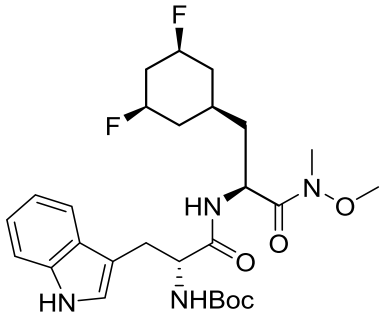

Dipeptide **28b** was prepared according to General Procedure G from **27b** (456.2 mg, 1.3 mmol), TFA (0.4 mL, 3.9 mmol) in 4.5 mL DCM, and Boc-D-tryptophan (593.5 mg, 1.95 mmol), EDC (605.4 mg, 3.9 mmol), and HOBt (632.3 mg, 3.9 mmol), Et_3N (1 mL, 7.8 mmol) in 2.5 mL x 2 DCM and was purified *via* flash column chromatography (hexane : ethyl acetate = 20 : 1

to 10 : 1 to 1 : 1 and TLC DCM : MeOH = 12 : 1) to give the product as a white solid (362 mg, 52%). M.p. 103-104 °C. ^{1}H NMR (400 MHz, Methanol-d_4) δ 7.60 (d, *J* = 7.9 Hz, 1H), 7.34 (d, *J* = 8.1 Hz, 1H), 7.13 (m, 1H), 7.10 (t, *J* = 7.9 Hz, 1H), 7.02 (t, *J* = 7.6 Hz, 1H), 4.89 (m, 1H), 4.43 (q, *J* = 6.9 Hz, 1H), 4.38 – 4.02 (m, 2H), 3.78 (s, 3H), 3.21 (dd, *J* = 7.8, 14.4 Hz, 2H), 3.16 (s, 3H), 2.23 (d, *J* = 157.0 Hz, 2H), 1.78 (m, 1H), 1.62 – 1.43 (m, 3H), 1.40 (s, 9H), 1.26 – 0.80 (m, 3H). ^{13}C NMR (126 MHz, Methanol-d_4) δ 174.4, 173.7, 157.5, 137.9, 128.9, 124.6, 122.4 (d, *J* = 13.4 Hz), 119.9, 119.4 (d, *J* = 8.0 Hz), 112.3, 110.8 (d, *J* = 15.8 Hz), 90.0 (t, *J* = 16.0 Hz), 88.6 (t, *J* = 15.9 Hz), 80.7, 62.2, 58.3, 56.9, 54.8, 48.1, 40.4 (t, *J* = 19.6 Hz), 39.7 (t, *J* = 17.2 Hz), 39.1, 38.0 (d, *J* = 18.0 Hz), 32.4, 29.3, 28.7, 27.9 (d, *J* = 12.0 Hz), 18.4. ^{19}F{^{1}H} NMR (377 MHz, Methanol-d_4) δ -175.7 (q, *J* = 12.8, 15.6 Hz). HRMS (ES$^+$) *m/z*: [M + Na]$^+$ calculated for $C_{27}H_{38}F_2N_4O_5Na$ 559.2703; found 559.2698.

Boc protected dipeptide (28c)

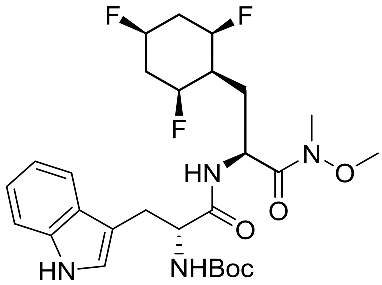

Dipeptide **28c** was prepared according to General Procedure G from **27c** (386 mg, 1.05 mmol), TFA (0.23 mL, 3.15 mmol) in 2.6 mL DCM, and Boc-D-tryptophan (480.9 mg, 1.58 mmol), EDC (489 mg, 3.15 mmol), and HOBt (510.7 mg, 3.15 mmol), Et_3N (0.65 mL, 6.3 mmol) in 1.9 mL x 2 DCM and was purified *via* flash column chromatography (DCM : MeOH = 50 : 1 and TLC DCM : MeOH = 12 : 1) to give the product as a white solid (243.5 mg, 42%). M.p. 107-109 °C. ^{1}H NMR (400 MHz, Methanol-d_4) δ 7.59 (d, *J* = 7.9 Hz, 1H), 7.32 (dt, *J* = 1.0, 8.1 Hz, 1H), 7.15 (m, 1H), 7.13 – 7.06 (m, 1H), 7.02 (t, *J*

= 7.5 Hz, 1H), 5.03 (d, *J* = 11.1 Hz, 1H), 4.68 (ddd, *J* = 3.4, 14.3, 48.0 Hz, 2H), 4.45 (d, *J* = 6.2 Hz, 1H), 4.37 (d, *J* = 38.5 Hz, 1H), 3.76 (s, 3H), 3.25 – 3.19 (m, 2H), 3.17 (s, 3H), 2.39 – 2.05 (m, 2H), 1.75 – 1.43 (m, 2H), 1.39 (d, *J* = 7.7 Hz, 9H), 1.34 – 1.08 (m, 2H). ^{13}C NMR (126 MHz, Methanol-d_4) δ 174.5, 157.6, 137.9, 128.8, 124.6, 122.5, 119.9, 119.5, 112.3, 110.9, 89.5, 88.1, 87.0, 86.7, 85.7, 80.7, 62.2, 56.8, 54.8, 47.7, 39.3, 34.8 (dt, *J* = 20.8, 42.2 Hz), 32.5, 30.9, 29.1, 28.6, 18.4. ^{19}F{^{1}H} NMR (377 MHz, Methanol-d_4) δ -178.1 (t, *J* = 13.7 Hz), -190.8 (t, *J* = 16.6 Hz), -193.4 (dd, *J* = 13.3, 18.7 Hz).

Boc protected dipeptide (28d)

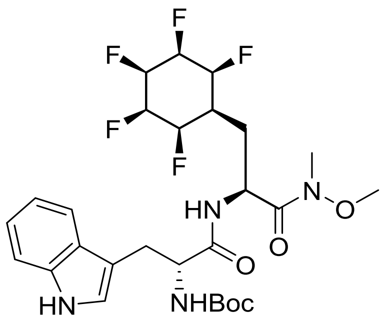

Dipeptide **28d** was prepared according to General Procedure G from **27d** (657.1 mg, 1.63 mmol), TFA (0.35 mL, 4.88 mmol) in 4 mL DCM, and Boc-D-tryptophan (760.8 mg, 2.5 mmol), EDC (760 mg, 4.9 mmol), and HOBt (794.4 mg, 4.9 mmol), Et_3N (1 mL, 9.75 mmol) in 2.9 mL x 2 DCM and was purified *via* flash column chromatography (DCM : MeOH = 50 : 1 to 20 : 1 and TLC DCM : MeOH = 12 : 1) to give the product as a white solid (141.2 mg, 15%). M.p. 113-114 °C. ^{1}H NMR (400 MHz, Methanol-d_4) δ 7.62 – 7.57 (m, 1H), 7.35 (d, *J* = 8.1 Hz, 1H), 7.19 (s, 1H), 7.12 (ddd, *J* = 1.2, 6.9, 8.1 Hz, 1H), 7.09 – 6.97 (m, 1H), 5.21 – 4.93 (m, 4H), 4.74 (d, *J* = 37.0 Hz, 1H), 4.63 – 4.38 (m, 2H), 4.16 – 3.83 (m, 2H), 3.73 (s, 3H), 3.28 – 3.19 (m, 2H), 3.16 (s, 3H), 3.05 (dd, *J* = 6.6, 14.6 Hz, 1H), 2.40 – 2.09 (m, 1H), 1.58 (q, *J* = 10.6, 12.1 Hz, 1H), 1.39 (d, *J* = 6.7 Hz, 9H). ^{13}C NMR (126 MHz, Methanol-d_4) δ 175.9, 174.5 (d, *J*

= 11.3 Hz), 157.7, 137.8 (d, J = 36.2 Hz), 128.9 (d, J = 8.0 Hz), 124.7 (d, J = 12.8 Hz), 124.4, 122.6, 122.3, 119.9 (d, J = 38.8 Hz), 119.3 (t, J = 19.9 Hz), 112.3 (d, J = 14.6 Hz), 111.0 (d, J = 16.3 Hz), 80.5, 62.2, 56.8, 55.9, 47.5 (d, J = 45.9 Hz), 32.4, 29.6, 28.9 – 28.3 (m). $^{19}F\{^1H\}$ NMR (377 MHz, Methanol-d_4) δ -205.6 (dt, J = 11.4, 66.1 Hz), -211.5 – -211.9 (m), -213.9 – -214.3 (m), -218.2 (dtt, J = 11.1, 25.4, 36.8 Hz). HRMS (ES$^+$) *m/z*: [M + Na]$^+$ calculated for $C_{27}H_{35}F_5N_4O_5Na$ 613.2420; found 613.2418.

Synthesis of indolones (General Procedure H)

A stirred solution of the dipeptide (1 eq.) in dry THF was cooled to -78°C. After stirring for 15 mins, a solution of DIBAL-H (1.0 M in hexane, 10 eq.) was added *via* a syringe pump over 1 h. The reaction was stirred for a further 4 h, then diluted with DCM (5 mL). MeOH (2 mL) was added dropwise and the reaction allowed to warm to room temperature. The mixture was washed with potassium hydrogen sulfate (1.0 M aq., 10 mL), dried over anhydrous Na_2SO_4 and filtered.

TFA (50 eq.) was added to the resulting organics. The reaction was stirred for 16 h, before being quenched with sodium bicarbonate (1.0 M aq., 10 mL), then the organics were separated and the aqueous further extracted with DCM (10 mL). Combined organics were dried over anhydrous Na_2SO_4, filtered and concentrated under reduced pressure to give the crude product. It was then purified *via* flash column chromatography.

Indolone (29a)

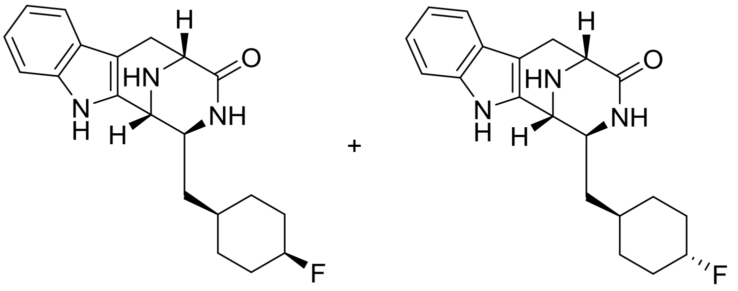

Indolone **29a** was prepared according to General Procedure H from **28a** (330 mg, 0.64 mmol), DIBAL-H (6.4 mL, 6.36 mmol) in 5 mL dry THF, and TFA (3 mL, 31.8 mmol) and was purified *via* flash column chromatography (DCM : MeOH = 50 : 1 and TLC DCM : MeOH = 19 : 1) to give the product as a yellow solid (99.9 mg, 46%). M.p. 268-270 °C. ^{1}H NMR (400 MHz, Methanol-d_4) δ 7.40 (d, *J* = 7.9 Hz, 1H), 7.36 – 7.28 (m, 1H), 7.15 – 7.05 (m, 1H), 7.05 – 6.94 (m, 1H), 5.31 (d, *J* = 10.4 Hz, 1H), 4.94 – 4.88 (m, 1H), 4.75 (m, 1H), 3.63 (dq, *J* = 6.8, 23.1 Hz, 2H), 3.13 (ddd, *J* = 2.7, 5.6, 15.4 Hz, 1H), 3.01 (ddd, *J* = 1.6, 3.3, 15.7 Hz, 1H), 2.01 (m, 2H), 1.79 – 1.52 (m, 6H), 1.41 (q, *J* = 10.3, 11.3 Hz, 3H). ^{13}C NMR (126 MHz, Chloroform-*d*) δ 170.9, 154.0, 136.1, 131.1, 126.8, 122.8 (d, *J* = 15.7 Hz), 120.2 (d, *J* = 16.7 Hz), 118.7 (d, *J* = 28.3 Hz), 111.3, 108.8, 88.7 (d, *J* = 167.4 Hz), 81.5 (d, *J* = 14.0 Hz), 55.7, 54.1, 53.6, 52.6, 49.8, 48.3, 41.9, 32.9, 30.4 (t, *J* = 22.0 Hz), 28.5 (d, *J* = 6.7 Hz), 27.3, 26.5, 25.3 (d, *J* = 44.4 Hz). ^{19}F{^{1}H} NMR (377 MHz, Methanol-d_4) δ -171.2, -185.8. HRMS (ES^{+}) *m/z*: [M + Na]$^{+}$ calculated for $C_{20}H_{24}FN_3ONa$ 364.1796; found 364.1790.

Indolone (29b)

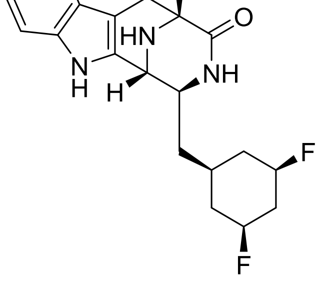

Indolone **29b** was prepared according to General Procedure H from **28b** (350 mg, 0.65 mmol), DIBAL-H (6.5 mL, 6.52 mmol) in 10 mL dry THF, and TFA (2.7 mL, 32.8 mmol) and was purified *via* flash column chromatography (DCM : MeOH = 50 : 1 and TLC DCM : MeOH = 19 : 1) to give the product as a yellow solid (70.5 mg, 30%). M.p. 270-271 °C. ^{1}H NMR (400 MHz, Methanol-d_4) δ 7.40 (d, J = 7.9 Hz, 1H), 7.33 – 7.29 (m, 1H), 7.10 (td, J = 1.6, 7.9 Hz, 1H), 7.01 (td, J = 1.2, 7.3 Hz, 1H), 5.28 (d, J = 19.0 Hz, 1H), 4.92 (d, J = 5.3 Hz, 1H), 4.69 – 4.43 (m, 2H), 3.67 (q, J = 6.4 Hz, 1H), 3.13 (dd, J = 5.6, 15.7 Hz, 1H), 3.06 – 2.96 (m, 1H), 2.41 (d, J = 140.0 Hz, 2H), 1.76 (dt, J = 6.9, 14.9 Hz, 2H), 1.60 (dt, J = 10.3, 21.2 Hz, 2H), 1.45 – 1.30 (m, 3H). ^{13}C NMR (126 MHz, Methanol-d_4) δ 173.7 – 172.6 (m), 155.4, 154.8, 137.8, 132.3 (d, J = 23.1 Hz), 127.8, 123.0 (d, J = 7.2 Hz), 120.3 (d, J = 5.6 Hz), 118.9 (d, J = 7.0 Hz), 112.3, 108.2 (d, J = 38.5 Hz), 90.9 – 89.8 (m), 88.8 (d, J = 16.0 Hz), 82.6 (d, J = 4.0 Hz), 61.5, 56.7 (d, J = 21.8 Hz), 55.3, 53.8, 50.9, 42.5 (d, J = 7.9 Hz), 41.0 – 40.0 (m), 39.7 – 38.3 (m), 32.7, 28.6 (d, J = 12.1 Hz), 28.3 – 27.5 (m), 26.2 (d, J = 63.5 Hz), 23.7, 20.9, 14.4 (d, J = 3.6 Hz). ^{19}F{^{1}H} NMR (377 MHz, Methanol-d_4) δ -175.6 (t, J = 3.4 Hz), -175.6 (t, J = 3.2 Hz). HRMS (ES$^+$) *m/z*: [M + Na]$^+$ calculated for $C_{20}H_{23}F_2N_3ONa$ 382.1701; found 382.1697.

Indolone (29d)

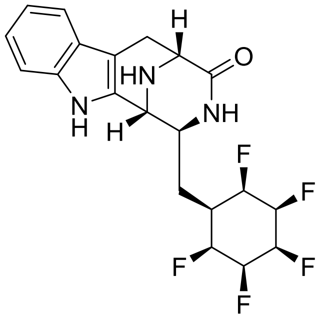

Indolone **29d** was prepared according to General Procedure H from **28d** (141.2 mg, 0.24 mmol), DIBAL-H (2.4 mL, 2.4 mmol) in 4 mL dry THF, and

TFA (0.98 mL, 12 mmol) and was purified *via* flash column chromatography (DCM : MeOH = 100 : 0 to 80 : 1 to 50 : 1 and TLC DCM : MeOH = 19 : 1) to give the product as a yellow solid (9.3 mg, 9%). M.p. 274-275 °C. ^{1}H NMR (700 MHz, Methanol-d_4) δ 7.43 (d, *J* = 7.9 Hz, 1H), 7.34 (d, *J* = 8.1 Hz, 1H), 7.13 (t, *J* = 7.6 Hz, 1H), 7.04 (t, *J* = 7.5 Hz, 1H), 5.37 (d, *J* = 32.3 Hz, 2H), 5.08 (d, *J* = 49.9 Hz, 1H), 4.97 (d, *J* = 5.5 Hz, 1H), 4.83 (m, 1H), 3.82 (q, *J* = 7.2, 8.1 Hz, 1H), 3.74 (m, 1H), 3.20 – 3.13 (m, 1H), 3.06 (d, *J* = 15.5 Hz, 1H), 2.29 – 2.15 (m, 1H), 2.17 – 1.88 (m, 1H), 1.36 (m, 2H). ^{13}C NMR (126 MHz, Methanol-d_4) δ 172.0, 155.4, 152.2, 137.8, 132.3 (d, *J* = 23.1 Hz), 127.8, 120.5, 123.0 (d, *J* = 7.2 Hz), 123.5 (d, *J* = 7.8 Hz), 118.9 (d, *J* = 7.0 Hz), 112.5, 112.3, 108.2 (d, *J* = 38.5 Hz), 90.9 – 89.8 (m), 88.8 (d, *J* = 16.0 Hz), 82.6 (d, *J* = 4.3 Hz), 56.7 (d, *J* = 21.8 Hz), 55.3, 53.8, 50.9, 42.5 (d, *J* = 8.1 Hz), 41.0 – 40.0 (m), 39.9, 32.1, 28.6 (d, *J* = 12.3 Hz), 27.2, 26.2 (d, *J* = 63.7 Hz), 23.8, 20.9, 14.4 (d, *J* = 3.5 Hz). $^{19}F\{^{1}H\}$ NMR (377 MHz, Methanol-d_4) δ -205.6 (dt, *J* = 10.8, 58.5 Hz), -211.5, -214.7 – -215.0 (m), -218.1 – -218.5 (m). HRMS (ES$^+$) *m/z*: [M + Na]$^+$ calculated for $C_{20}H_{20}F_5N_3ONa$ 436.1419; found 436.1415.

NMR Spectra

(19a)

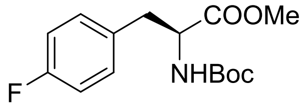

^{1}H NMR (400 MHz, Chloroform-*d*)

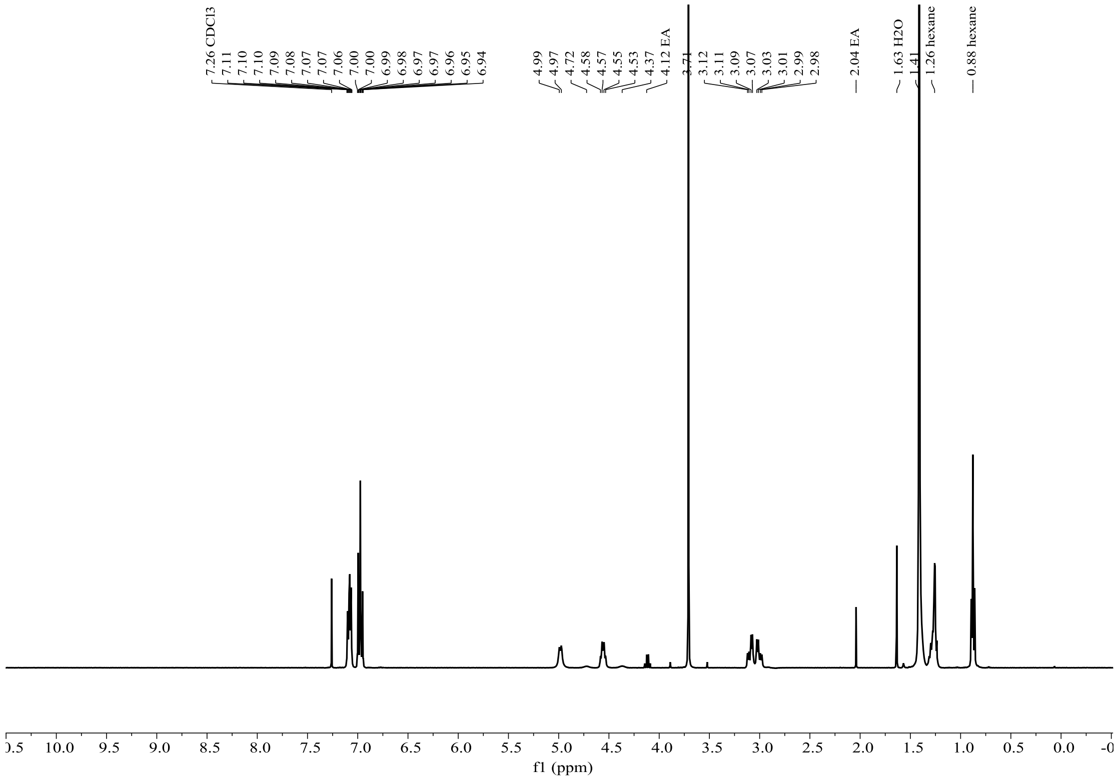

$^{19}F\{^1H\}$ NMR (377 MHz, Chloroform-*d*)

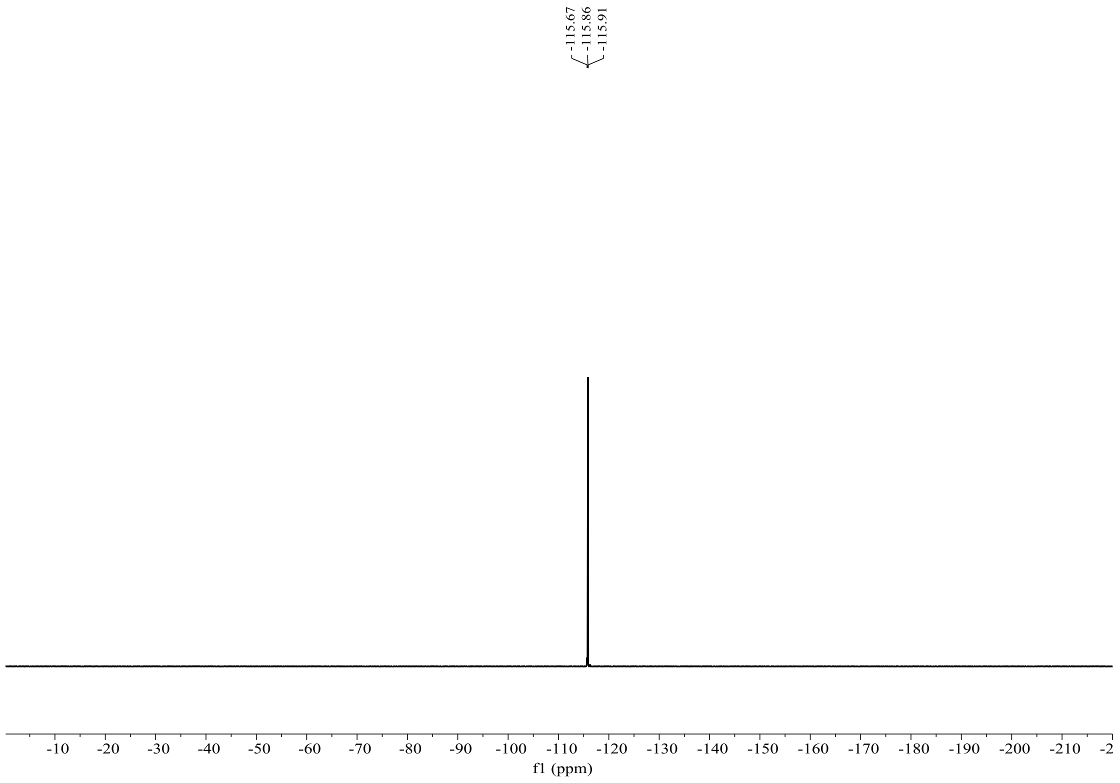

(19b)

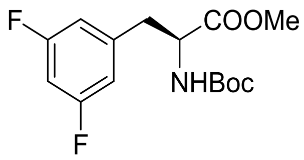

^{1}H NMR (400 MHz, Chloroform-*d*)

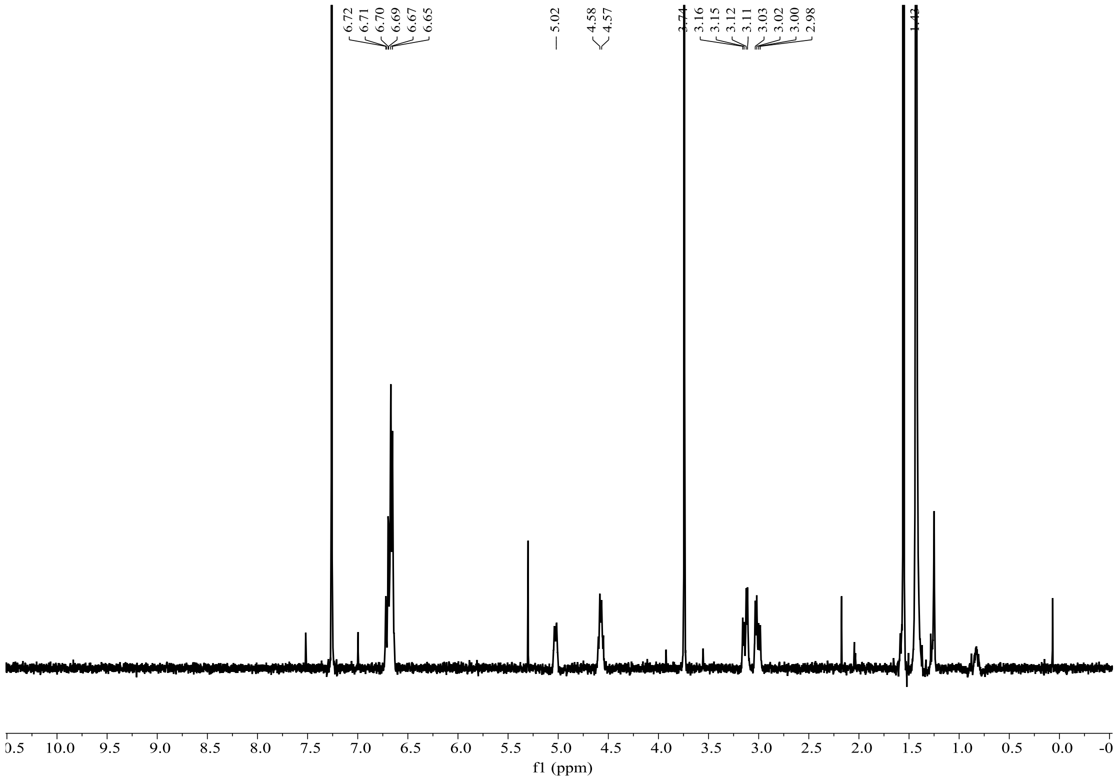

^{13}C NMR (126 MHz, Chloroform-*d*)

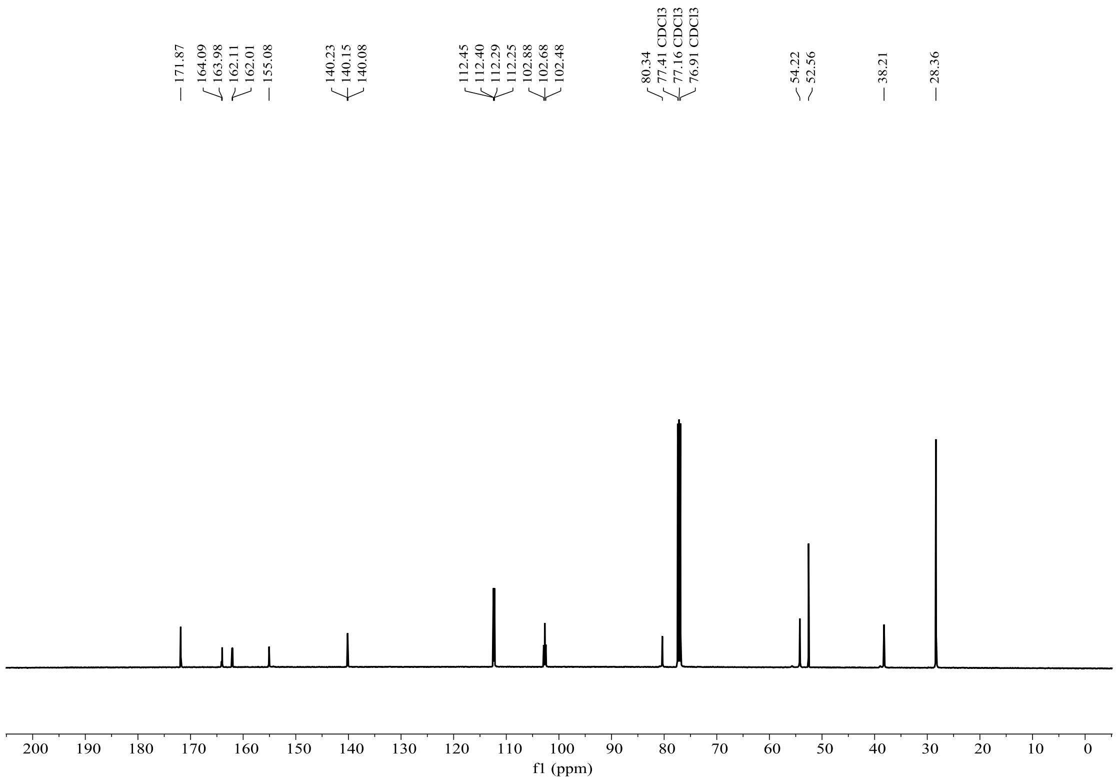

$^{19}F\{^{1}H\}$ NMR (376 MHz, Chloroform-*d*)

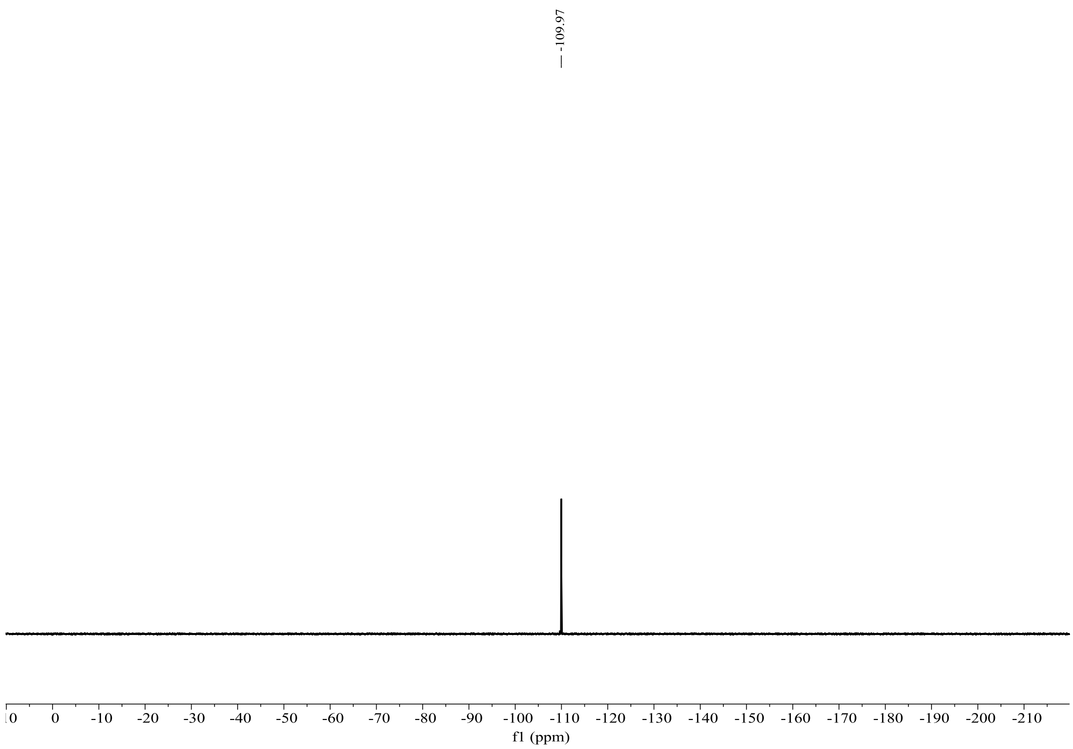

(19d)

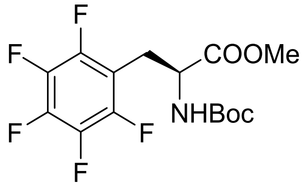

^{1}H NMR (400 MHz, Chloroform-*d*)

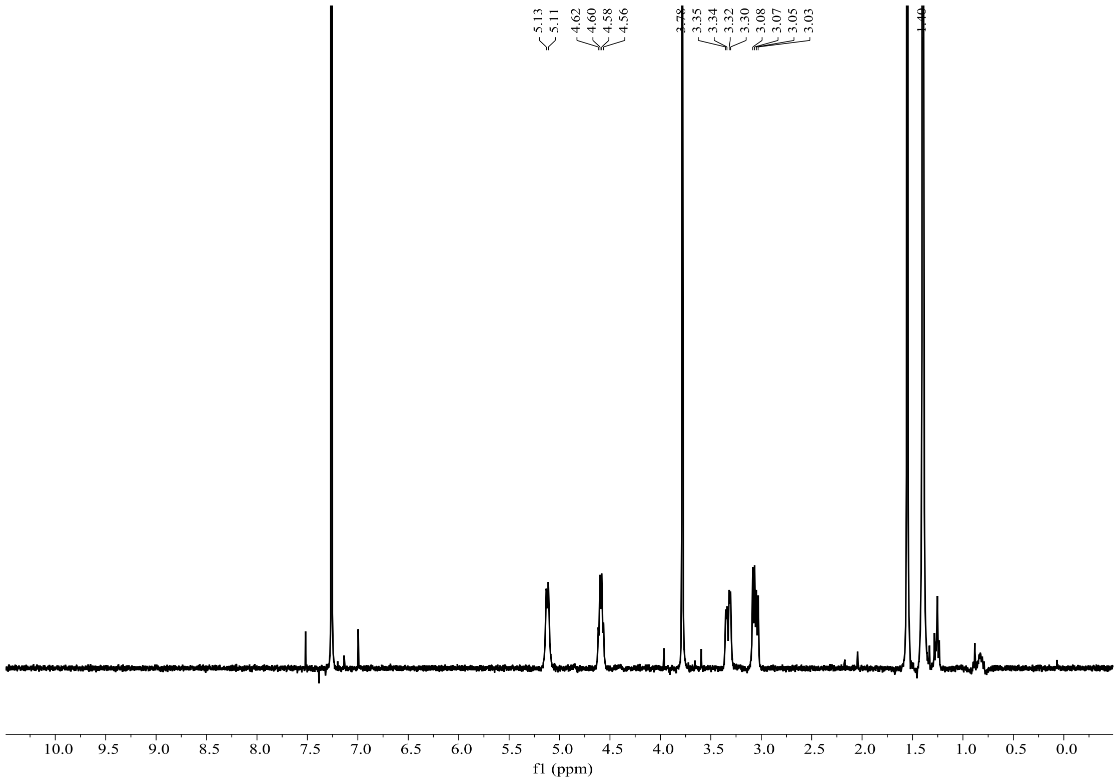

$^{19}F\{^{1}H\}$ NMR (377 MHz, Chloroform-*d*)

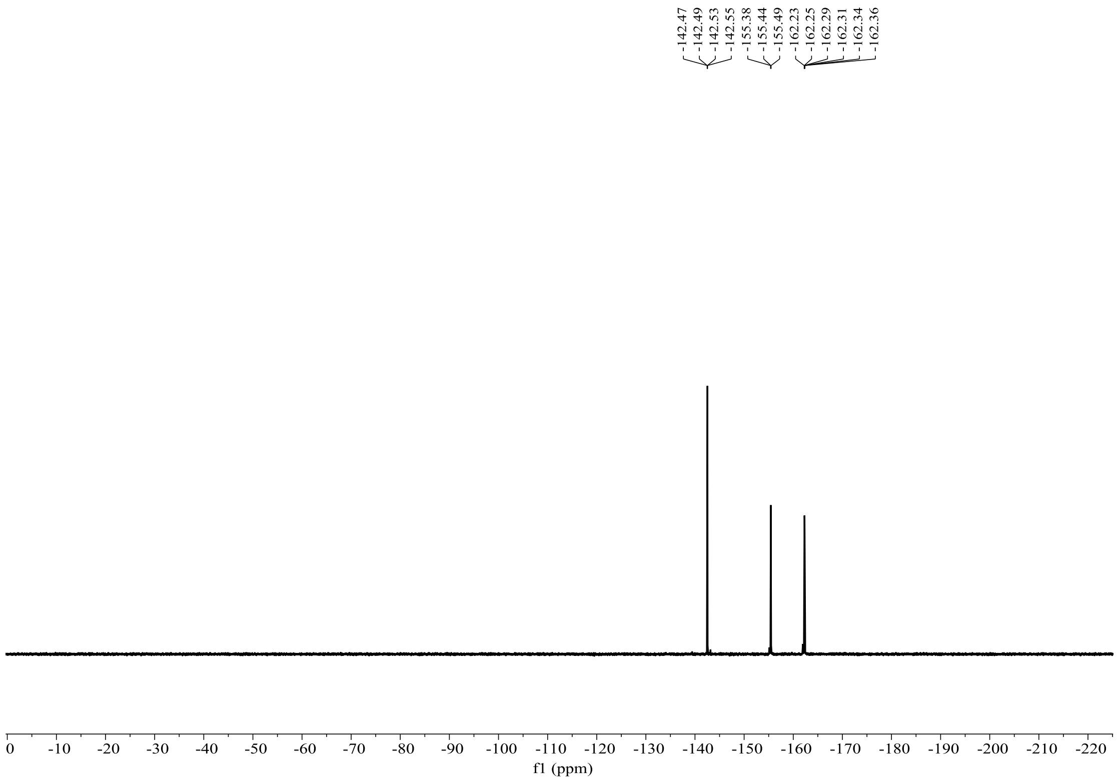

(17)

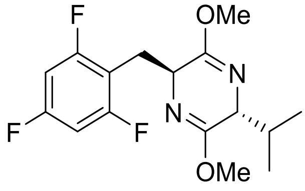

^{1}H NMR (400 MHz, Chloroform-*d*)

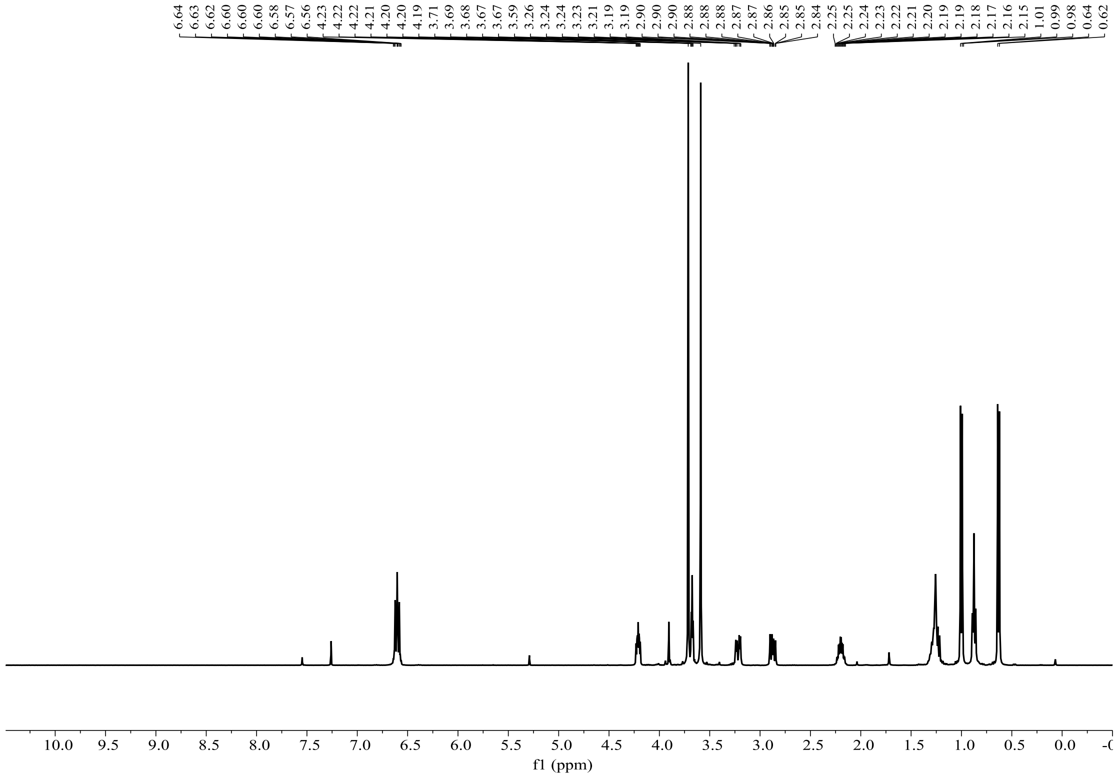

^{13}C NMR (126 MHz, Chloroform-*d*)

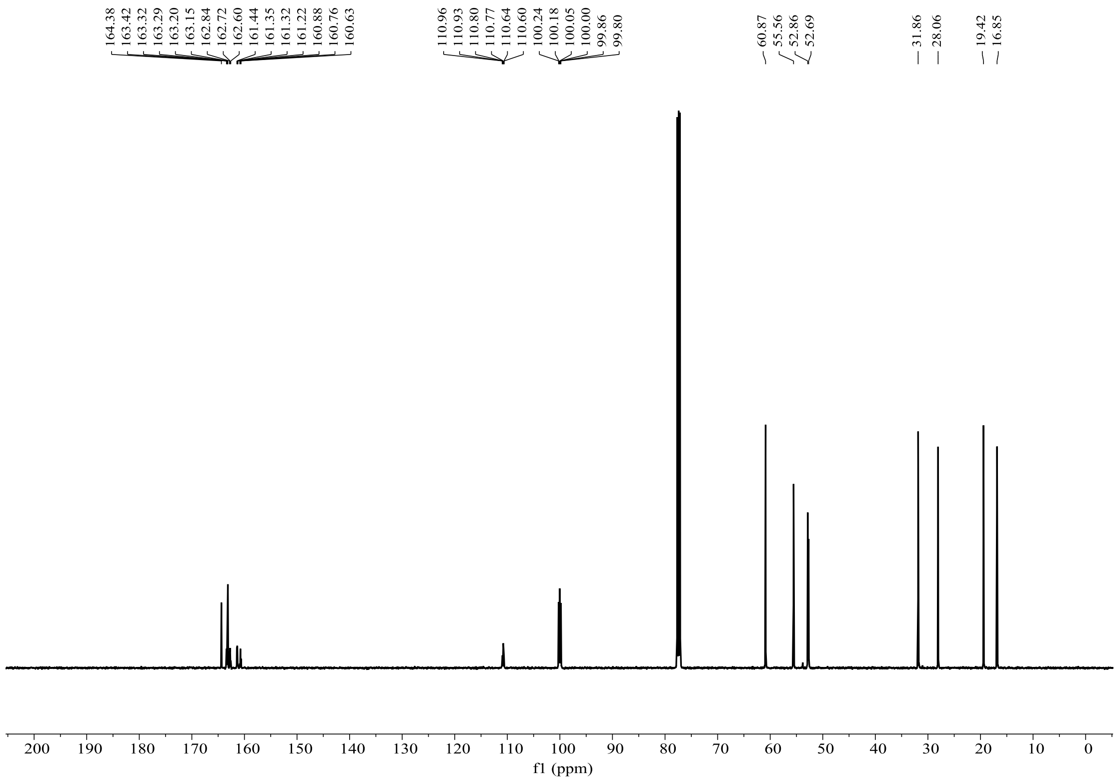

$^{19}F\{^1H\}$ NMR (377 MHz, Chloroform-*d*)

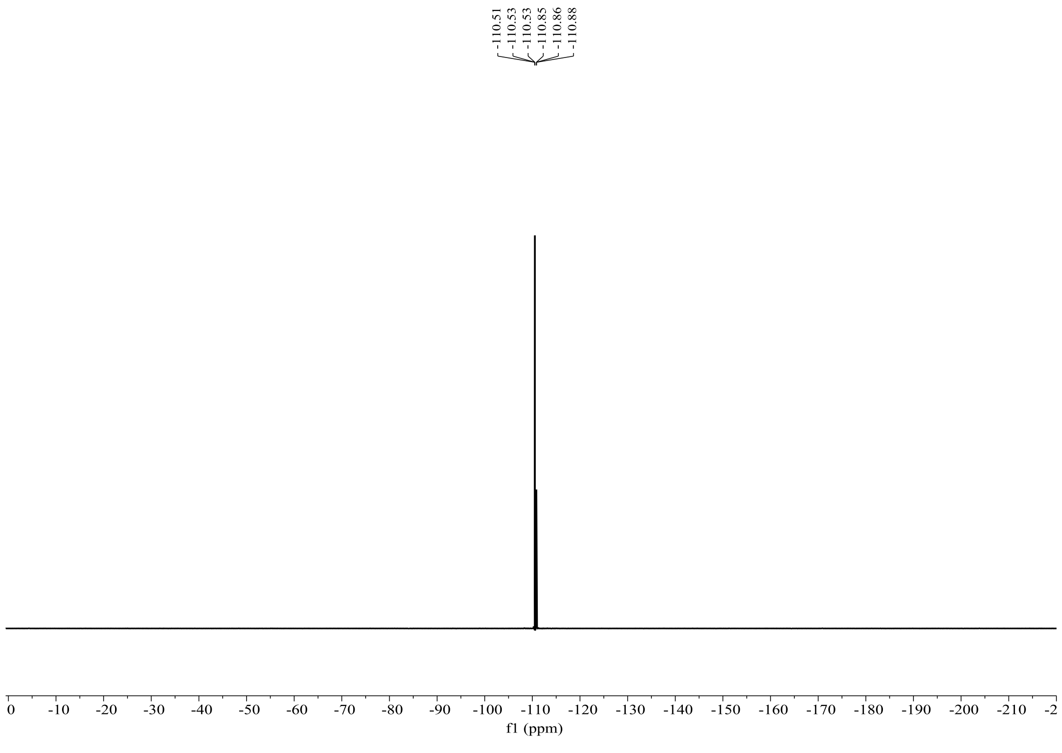

(19c)

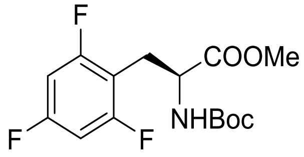

^{1}H NMR (400 MHz, Chloroform-*d*)

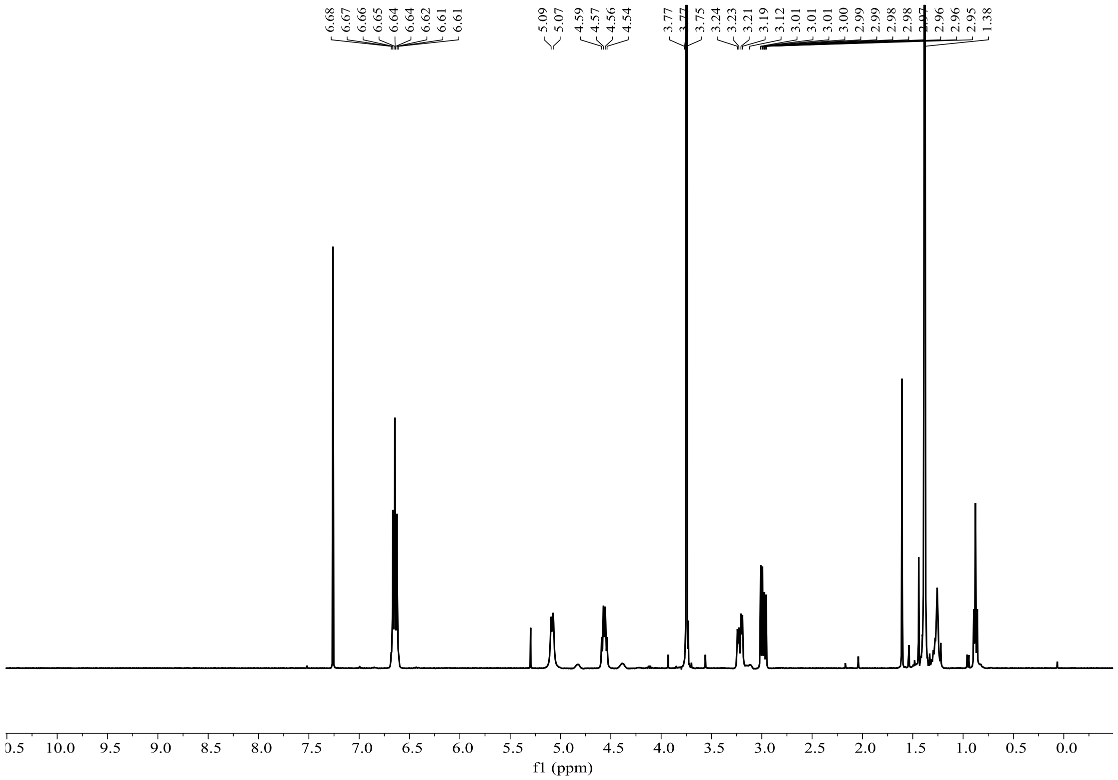

^{13}C NMR (126 MHz, Chloroform-*d*)

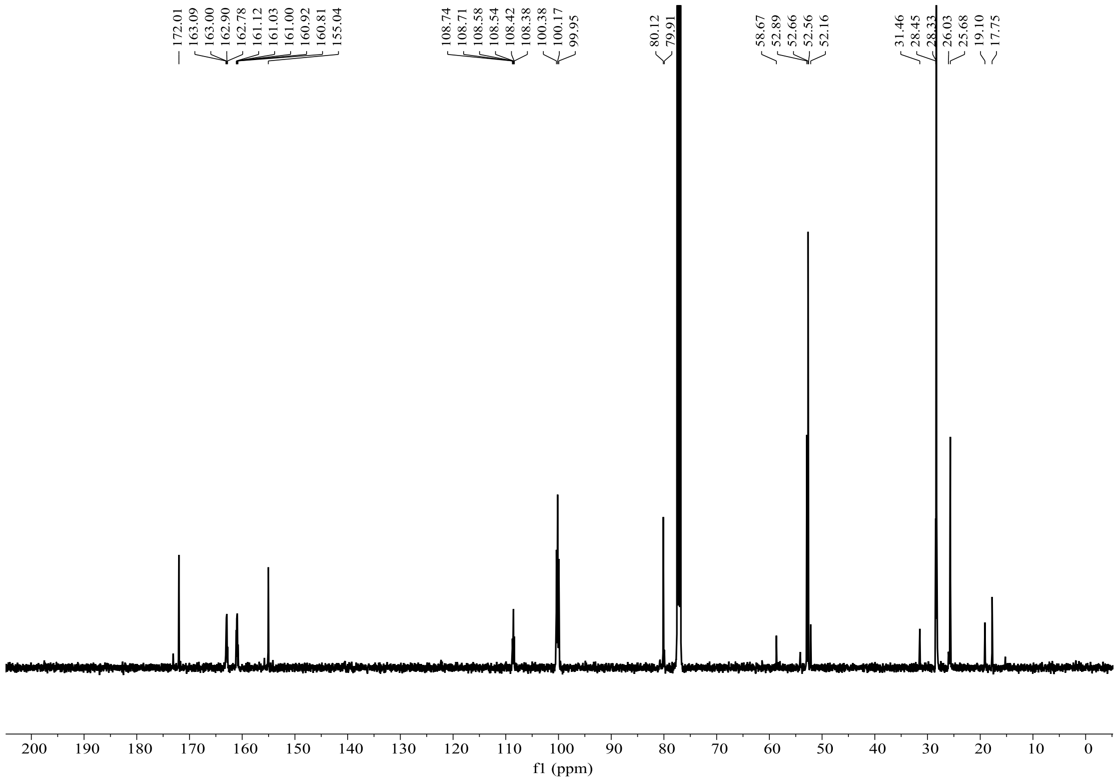

^{19}F{^{1}H} NMR (376 MHz, Chloroform-*d*)

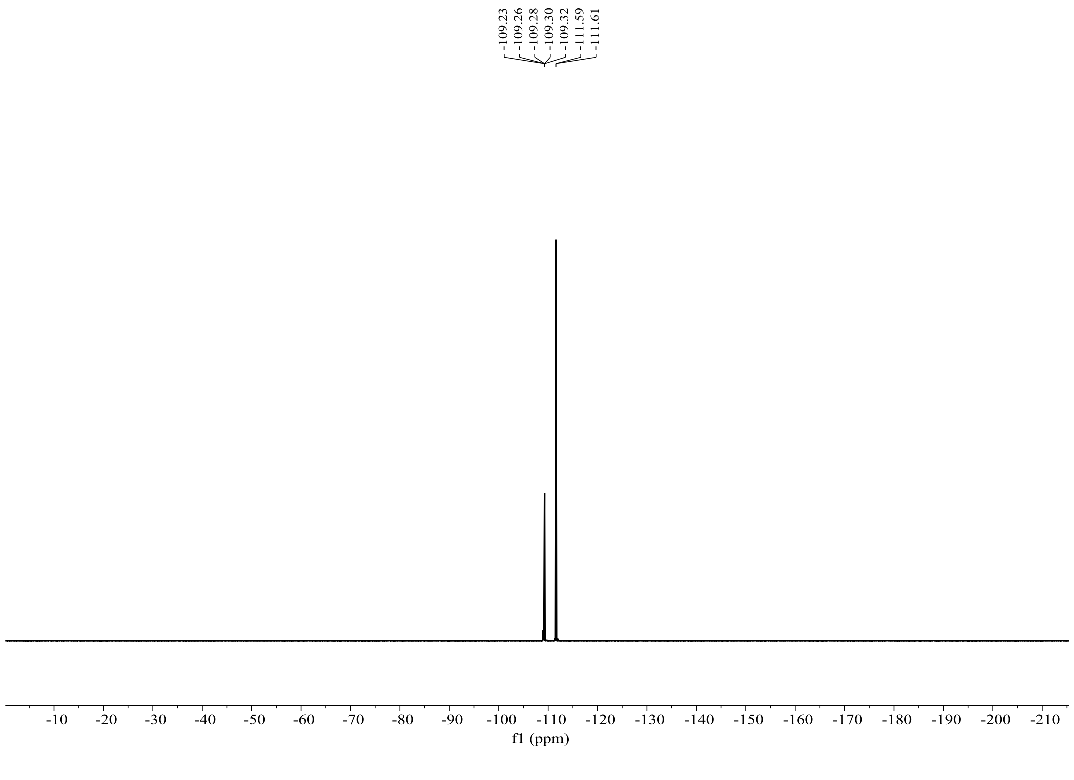

(20a)

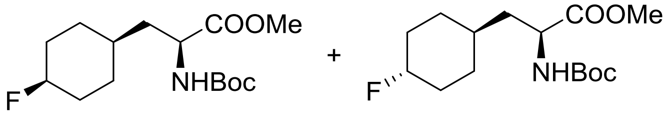

^{1}H NMR (400 MHz, Chloroform-*d*)

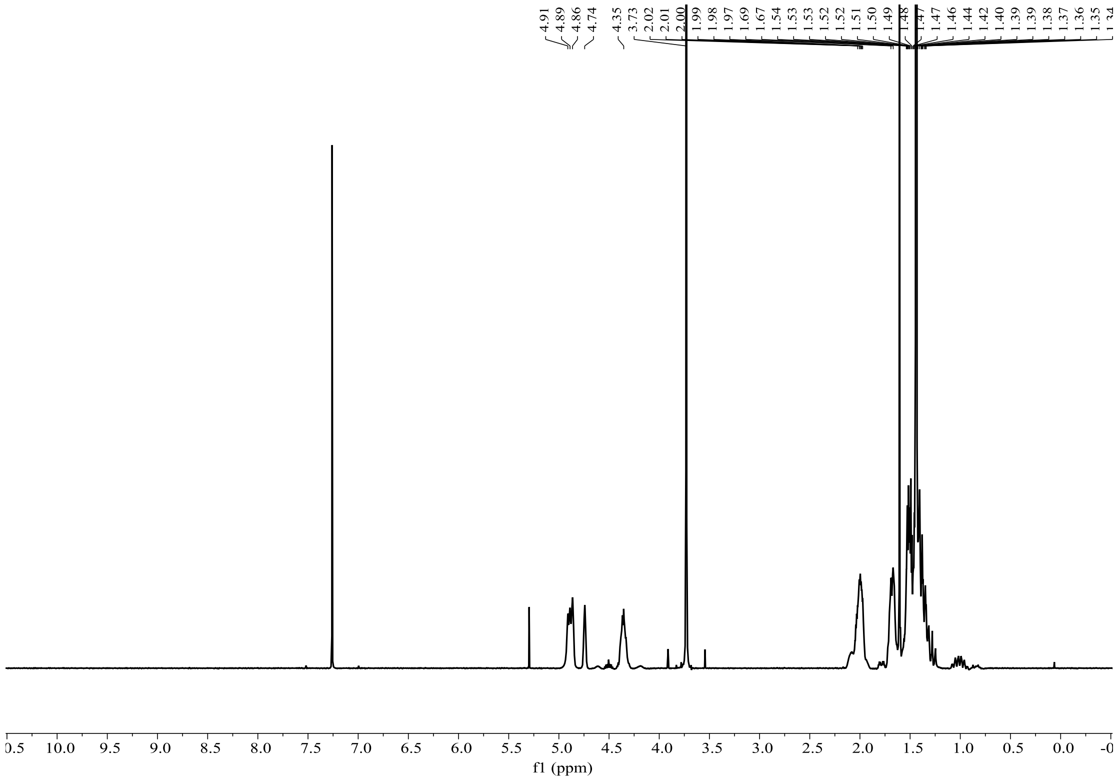

^{13}C NMR (126 MHz, Chloroform-*d*)

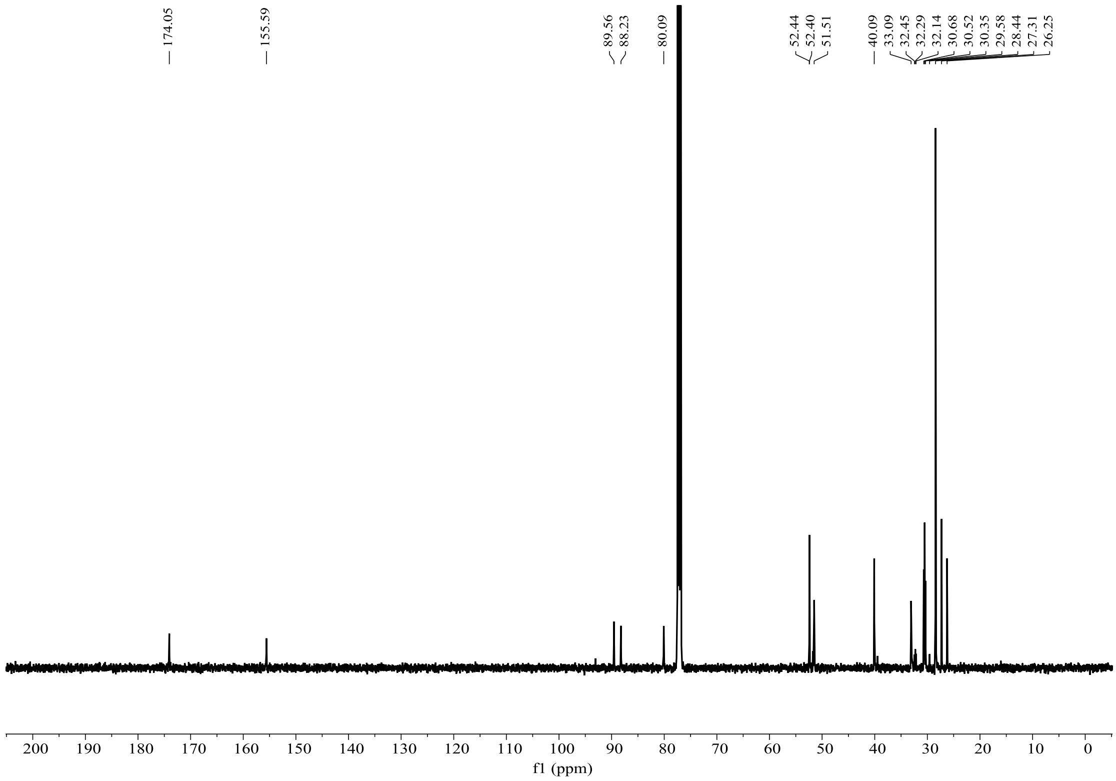

$^{19}F\{^{1}H\}$ NMR (376 MHz, Chloroform-*d*)

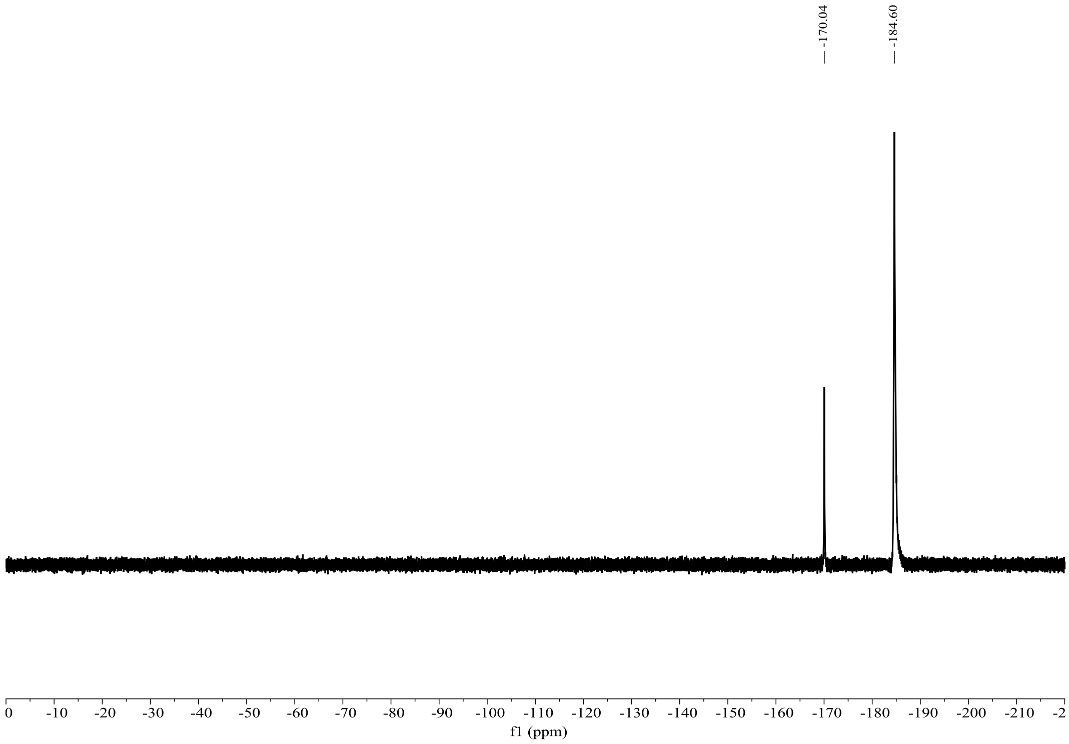

(20b)

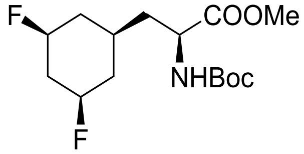

^{1}H NMR (400 MHz, Chloroform-*d*)

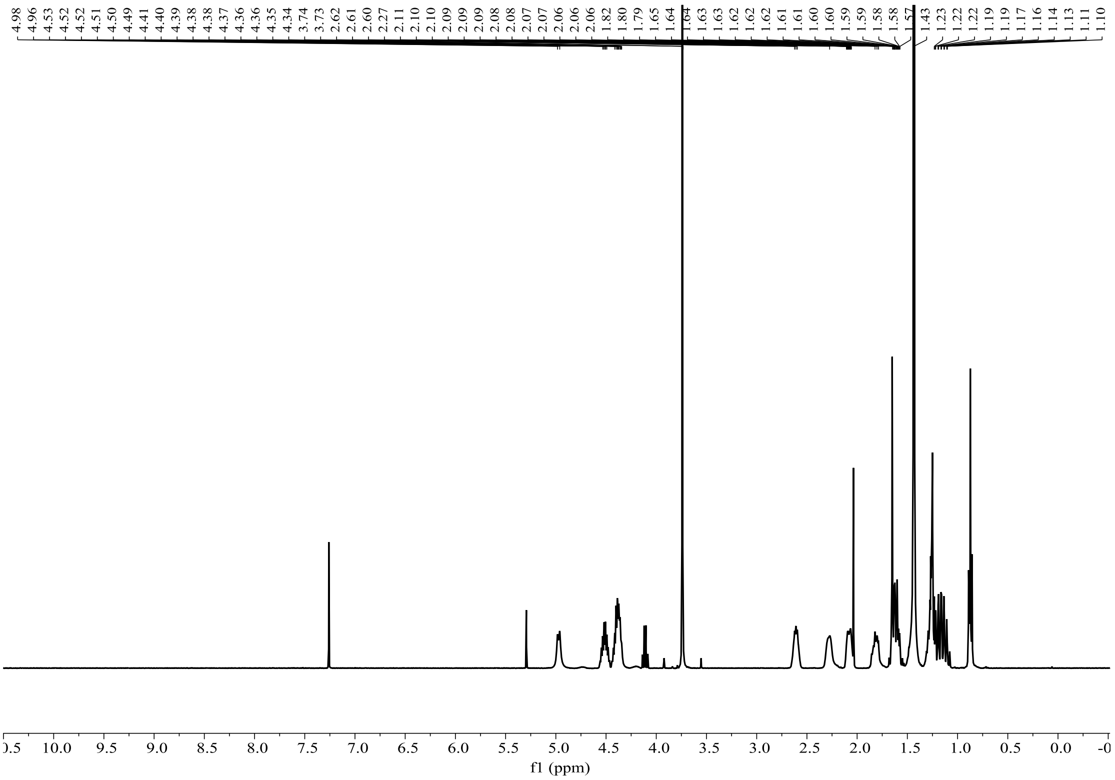

^{13}C NMR (126 MHz, Chloroform-*d*)

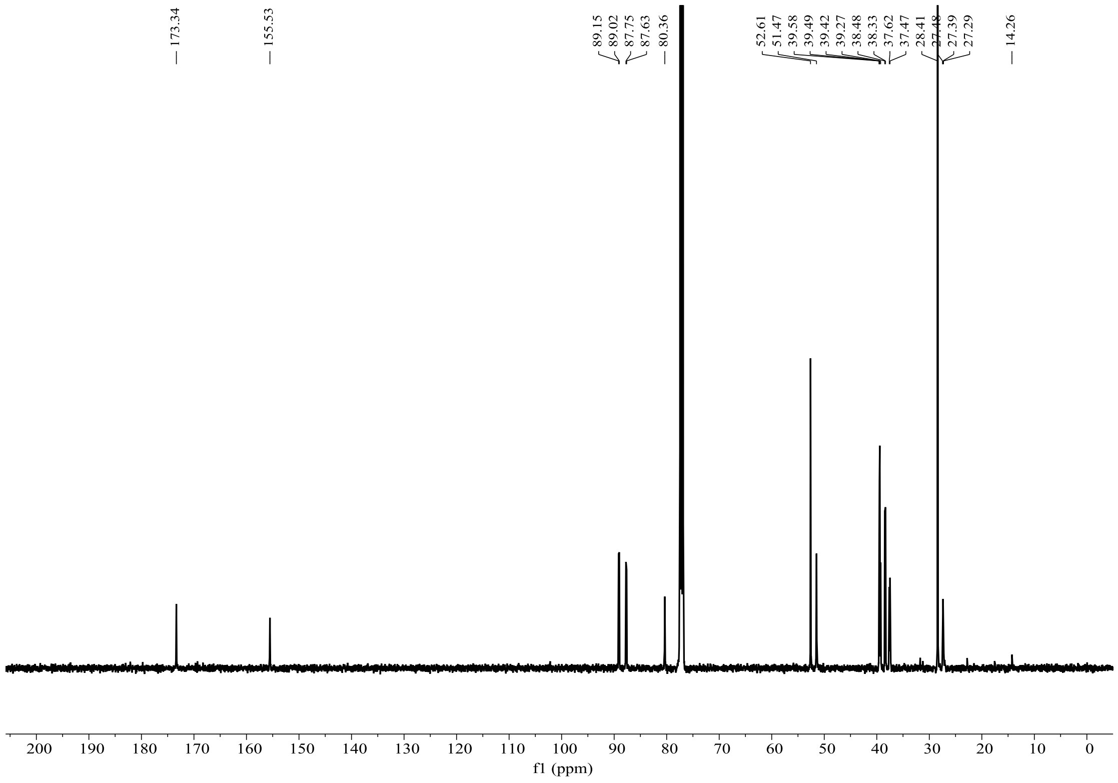

$^{19}F\{^{1}H\}$ NMR (377 MHz, Chloroform-*d*)

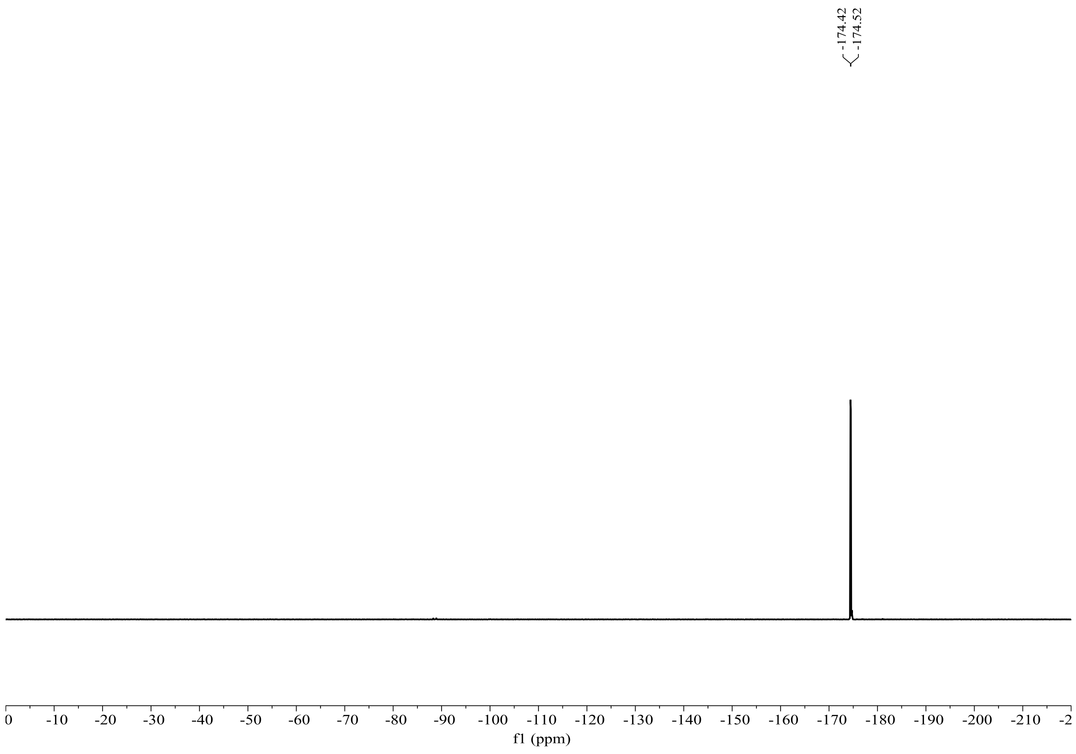

(20c)

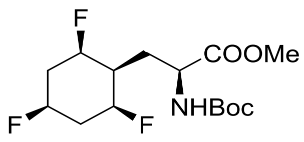

^{1}H NMR (400 MHz, Chloroform-*d*)

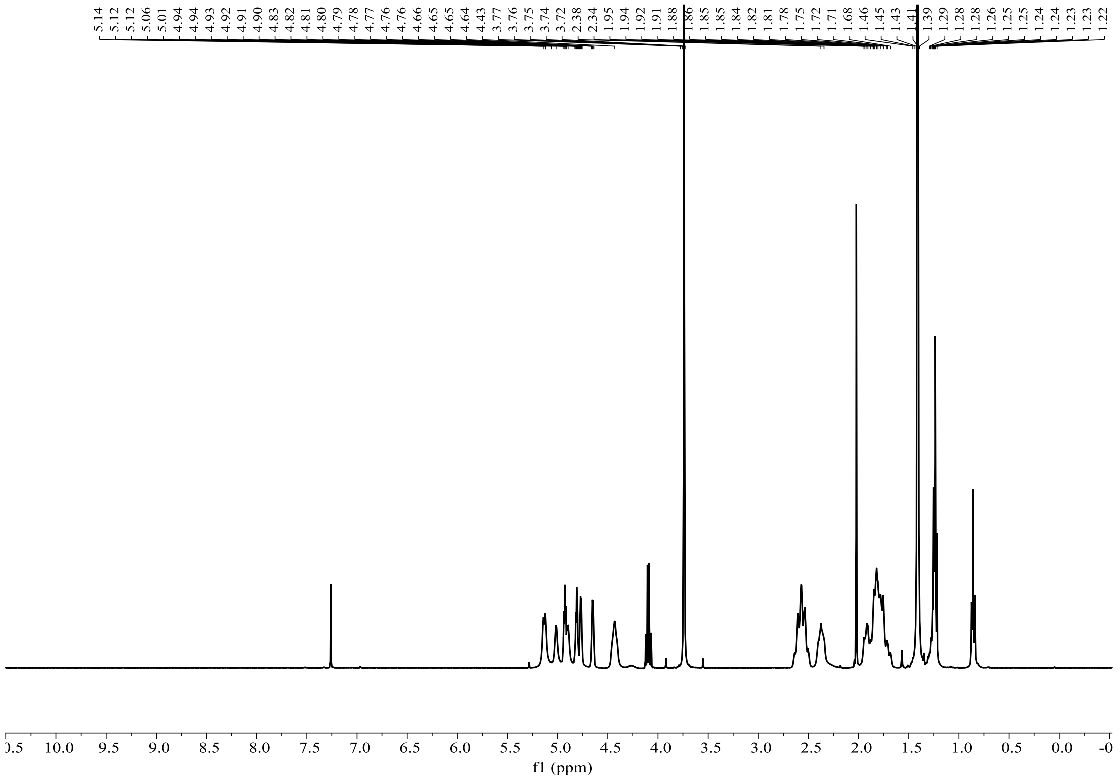

^{13}C NMR (126 MHz, Chloroform-*d*)

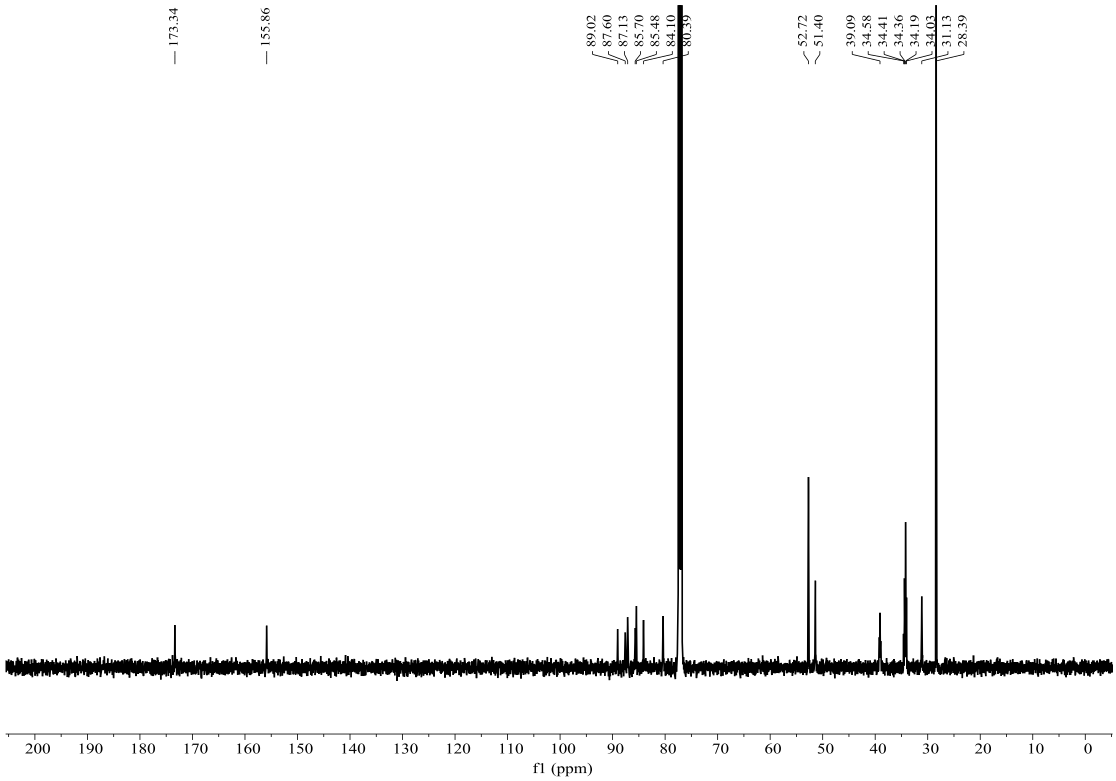

$^{19}F\{^1H\}$ NMR (377 MHz, Chloroform-*d*)

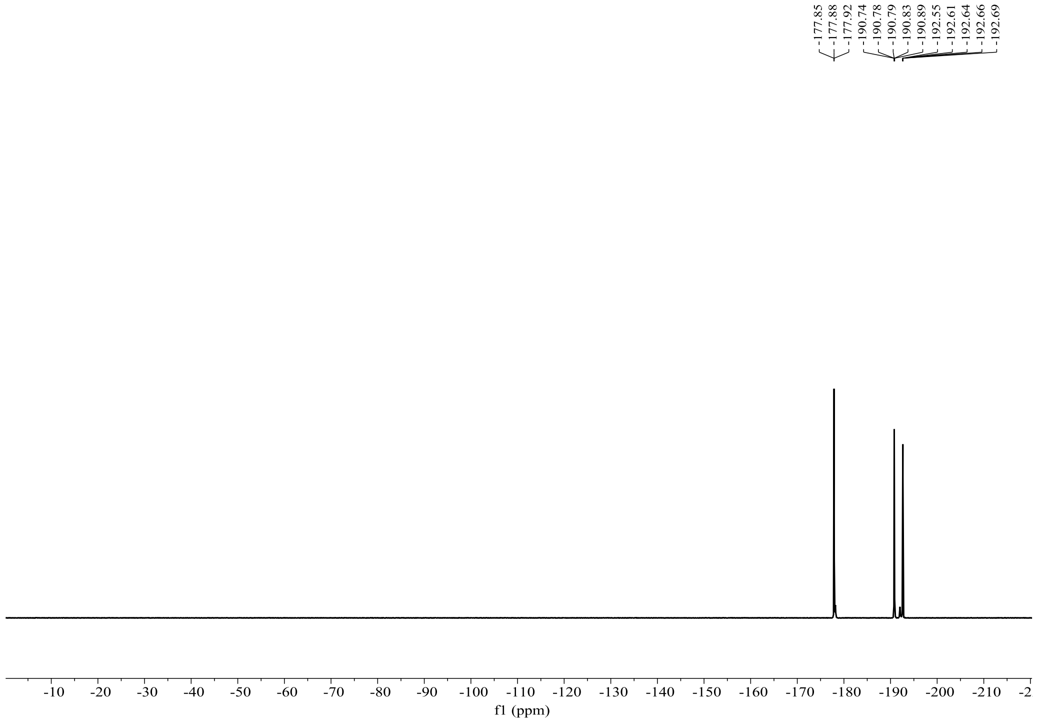

(20d)

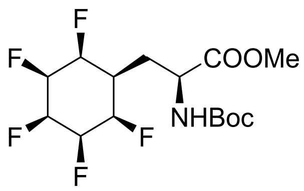

^{1}H NMR (400 MHz, Chloroform-*d*)

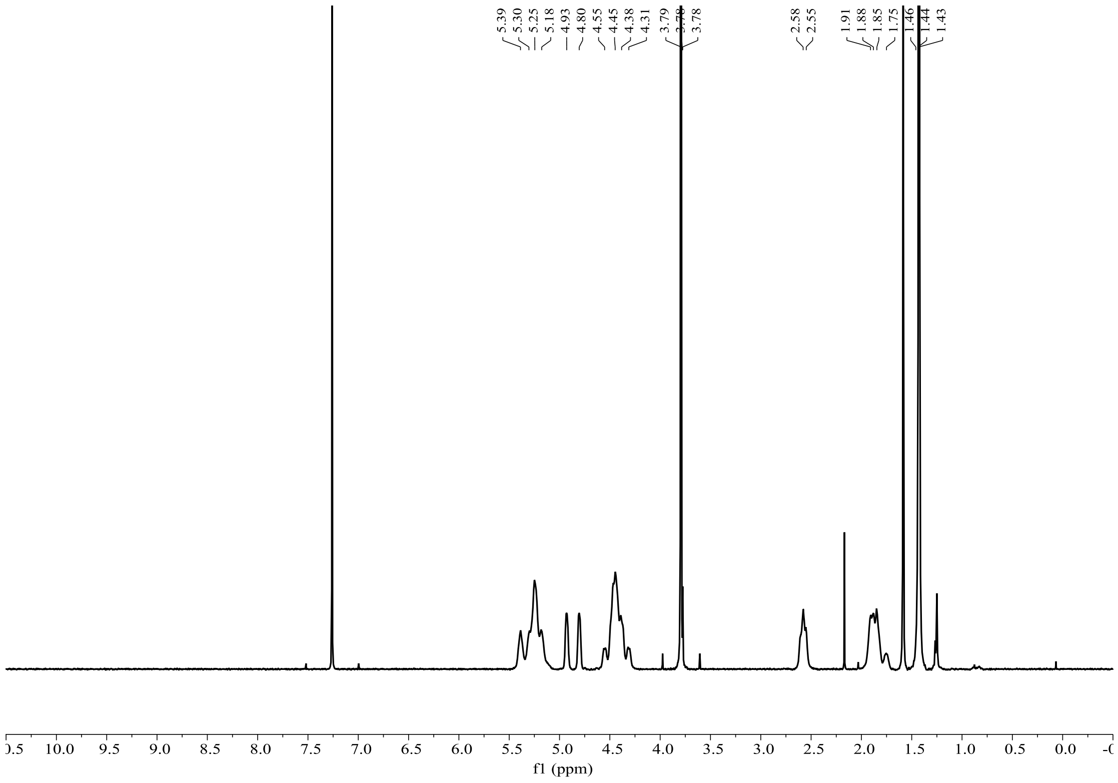

$^{19}F\{^{1}H\}$ NMR (377 MHz, Chloroform-*d*)

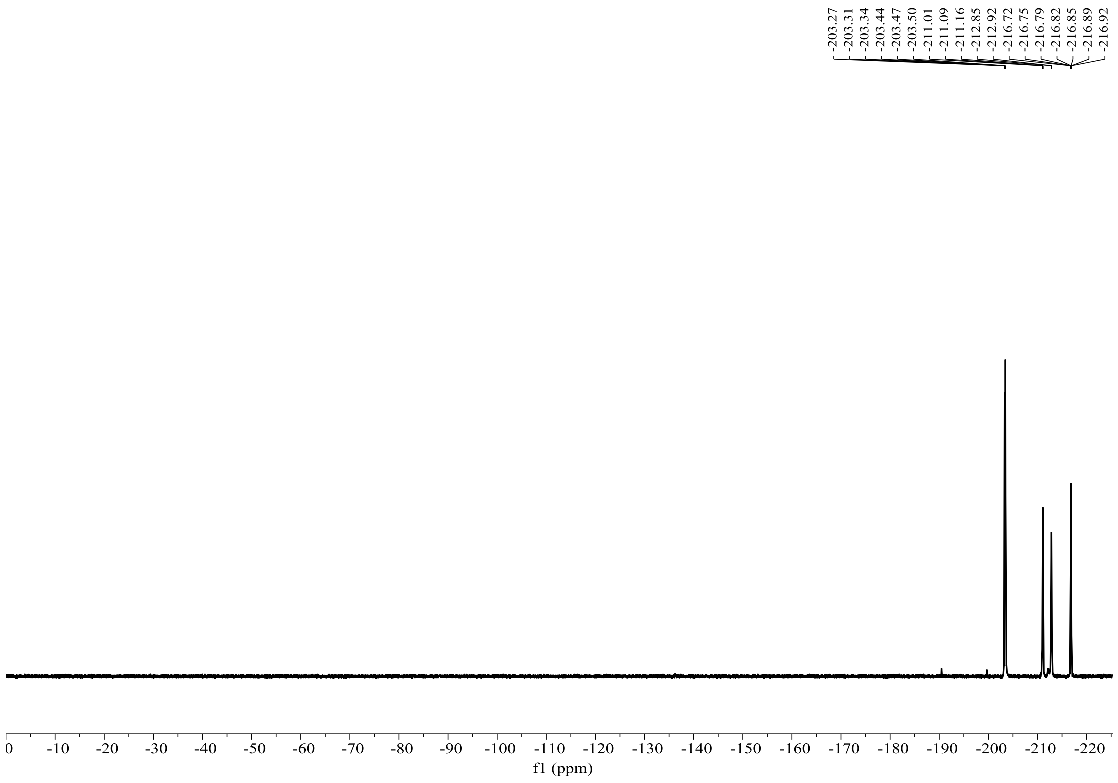

(21a)

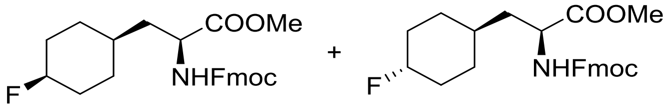

^{1}H NMR (400 MHz, Chloroform-*d*)

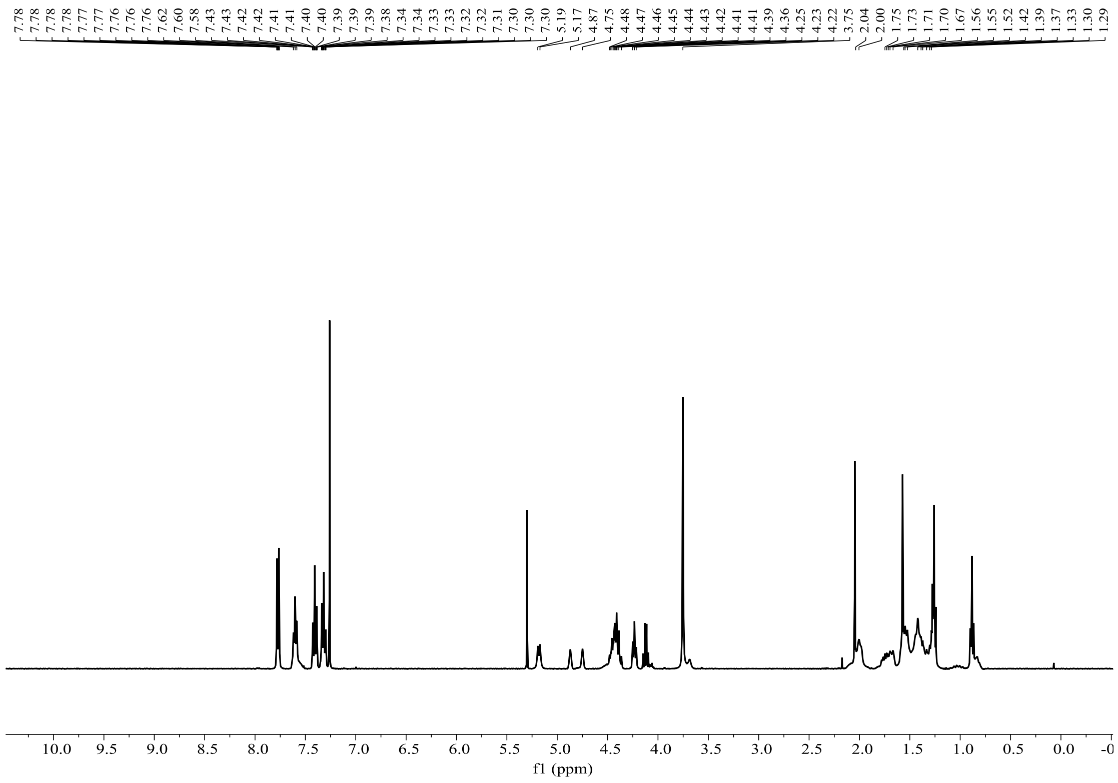

^{13}C NMR (126 MHz, Chloroform-*d*)

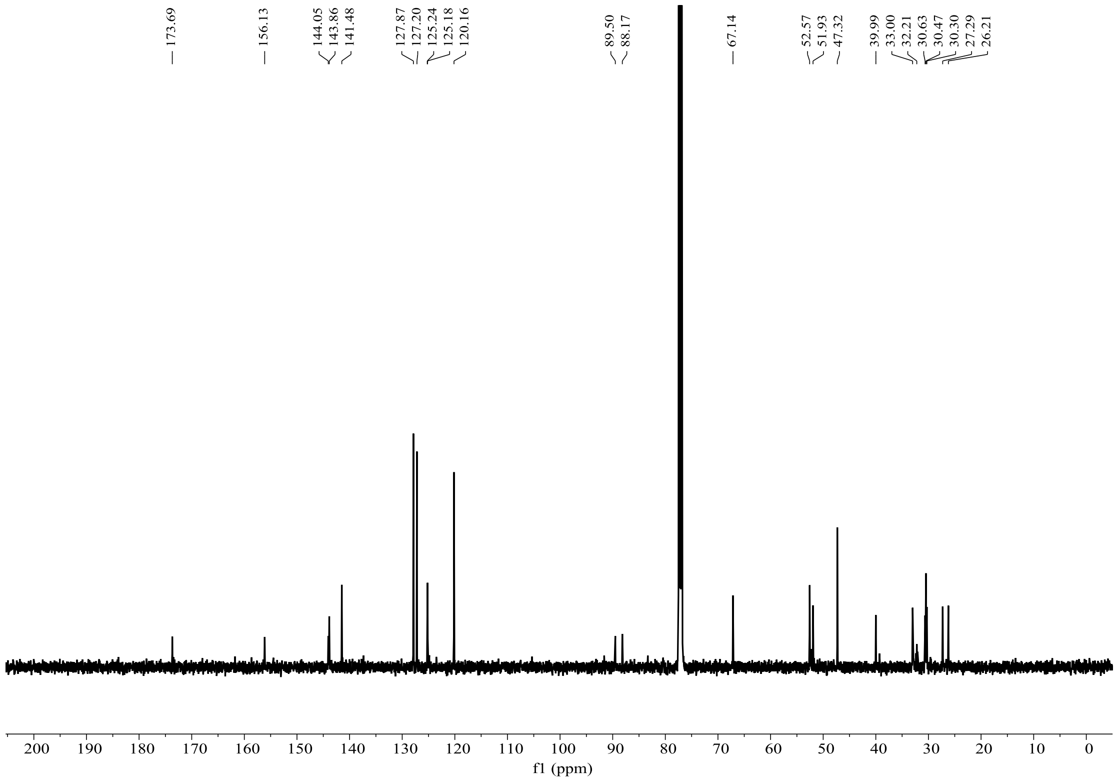

$^{19}F\{^1H\}$ NMR (377 MHz, Chloroform-*d*)

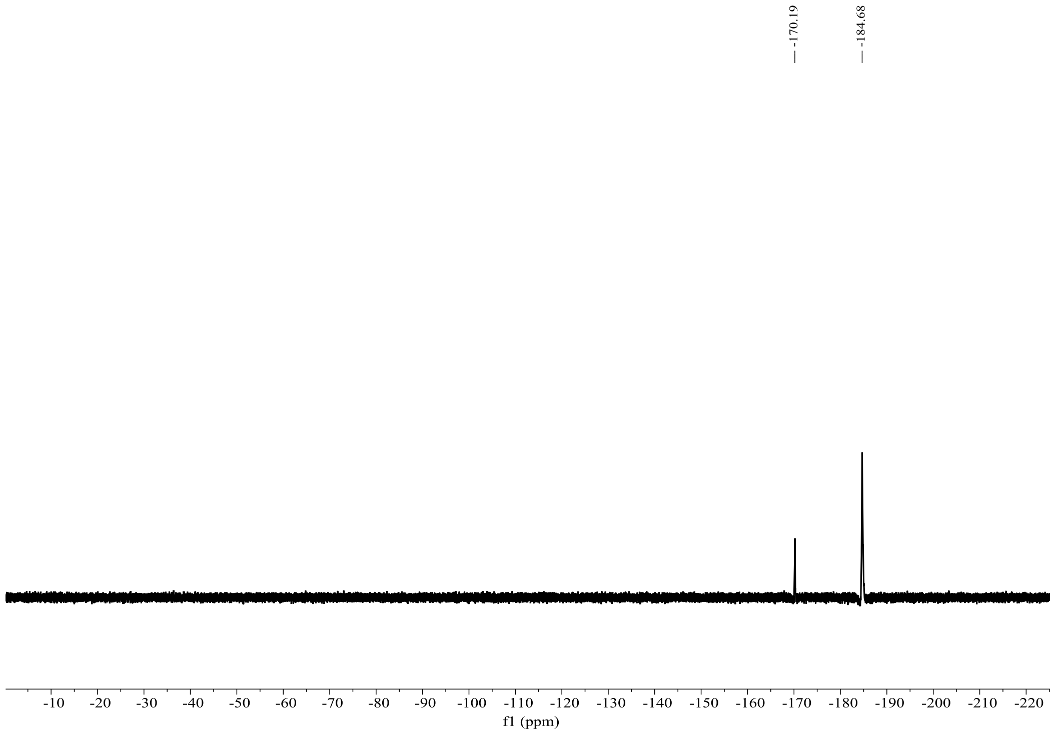

(21b)

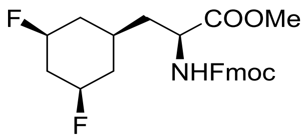

^{1}H NMR (400 MHz, Chloroform-*d*)

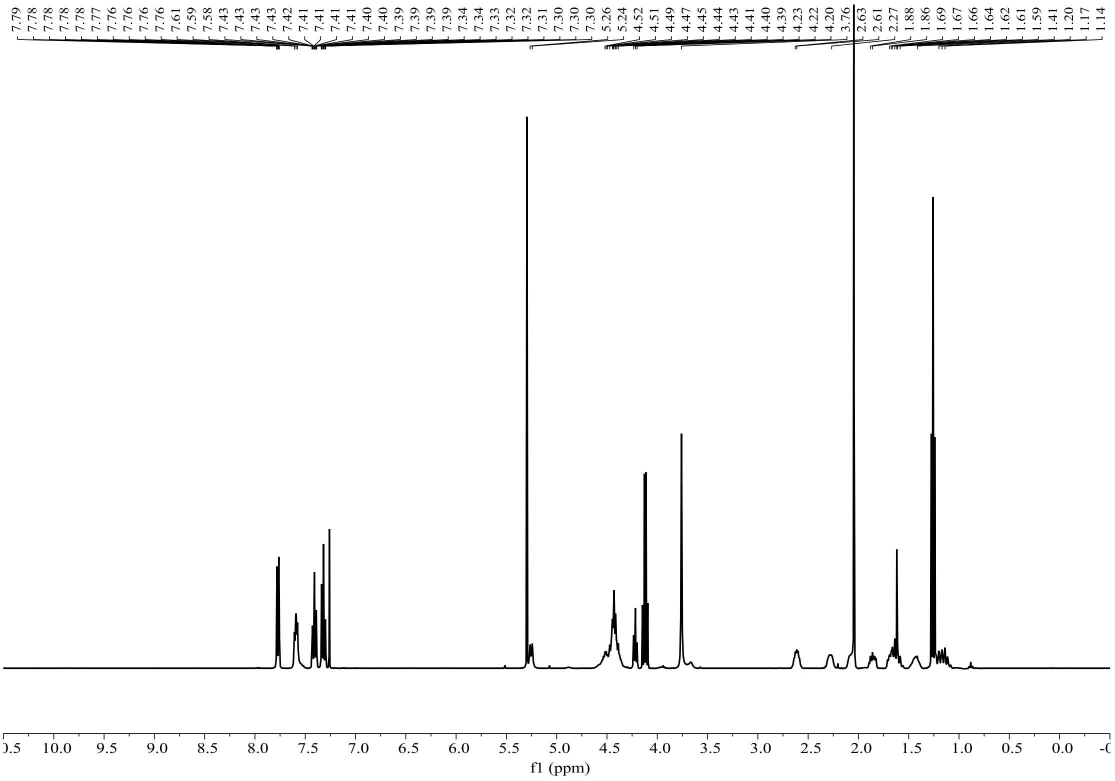

^{13}C NMR (126 MHz, Chloroform-*d*)

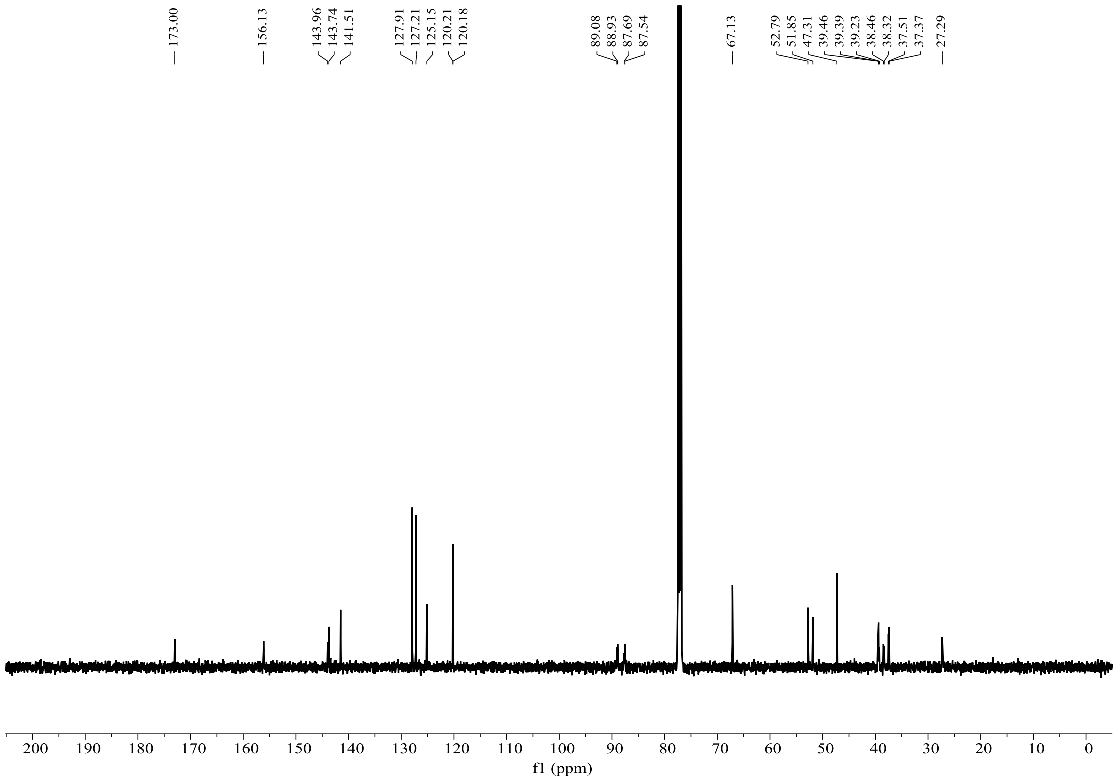

^{19}F{^{1}H} NMR (377 MHz, Chloroform-*d*)

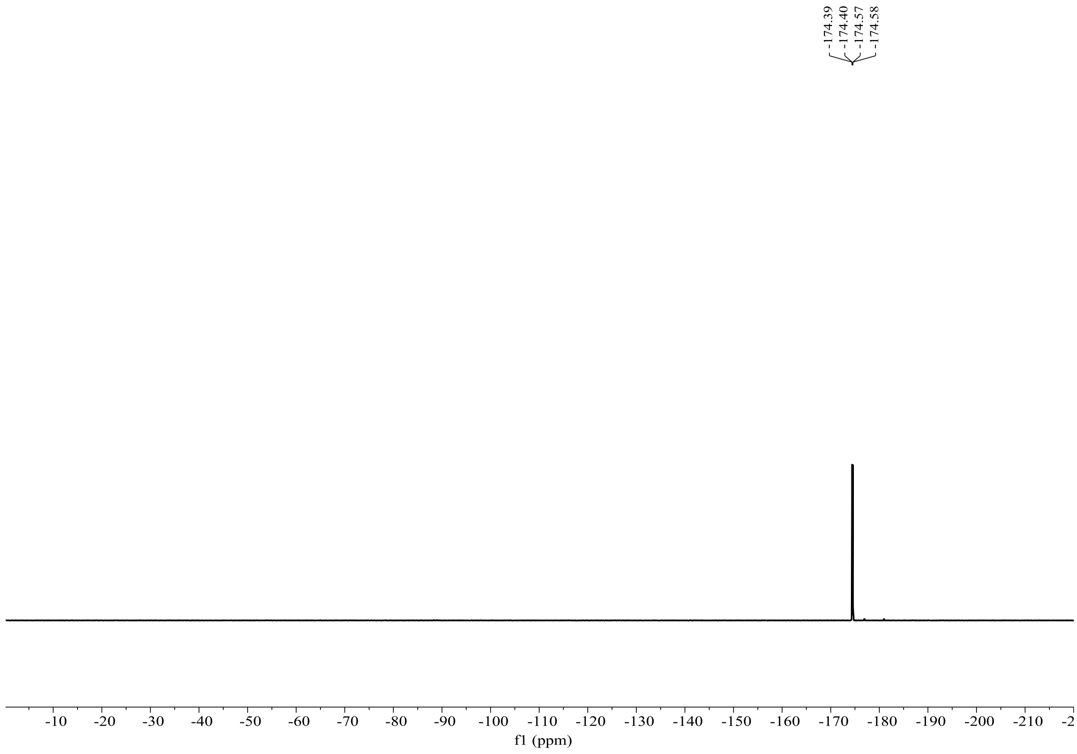

(21c)

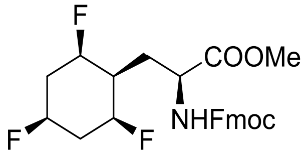

^{1}H NMR (400 MHz, Chloroform-*d*)

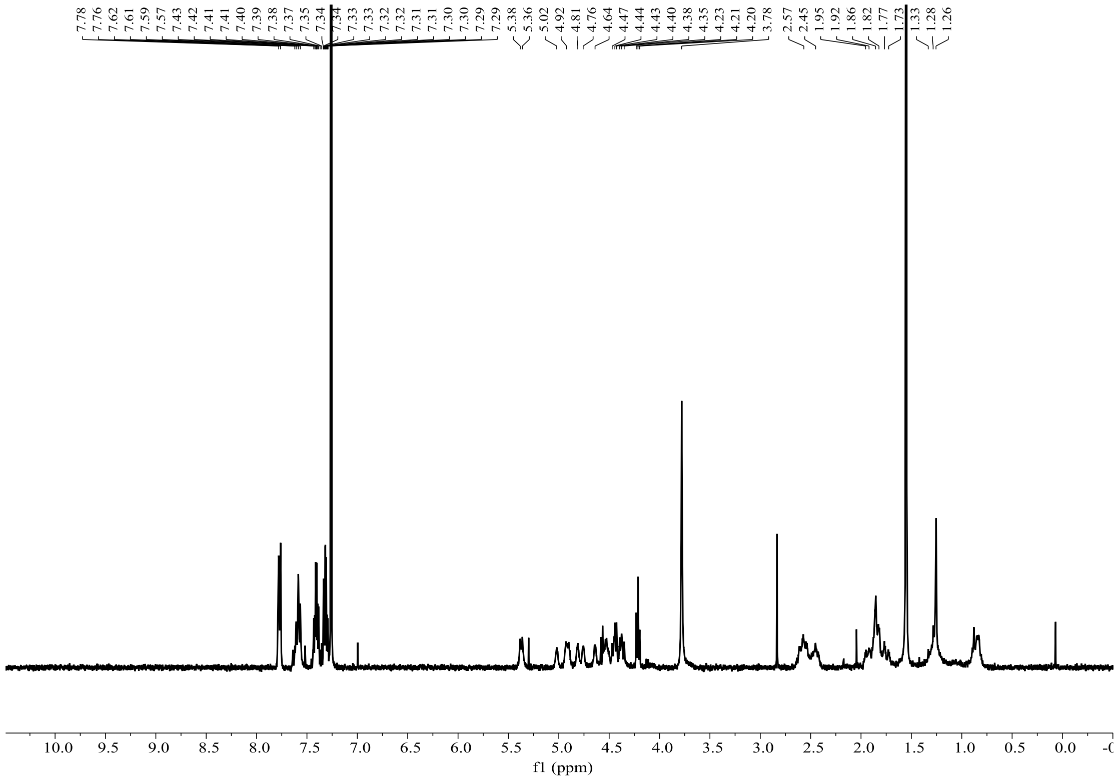

^{13}C NMR (126 MHz, Chloroform-*d*)

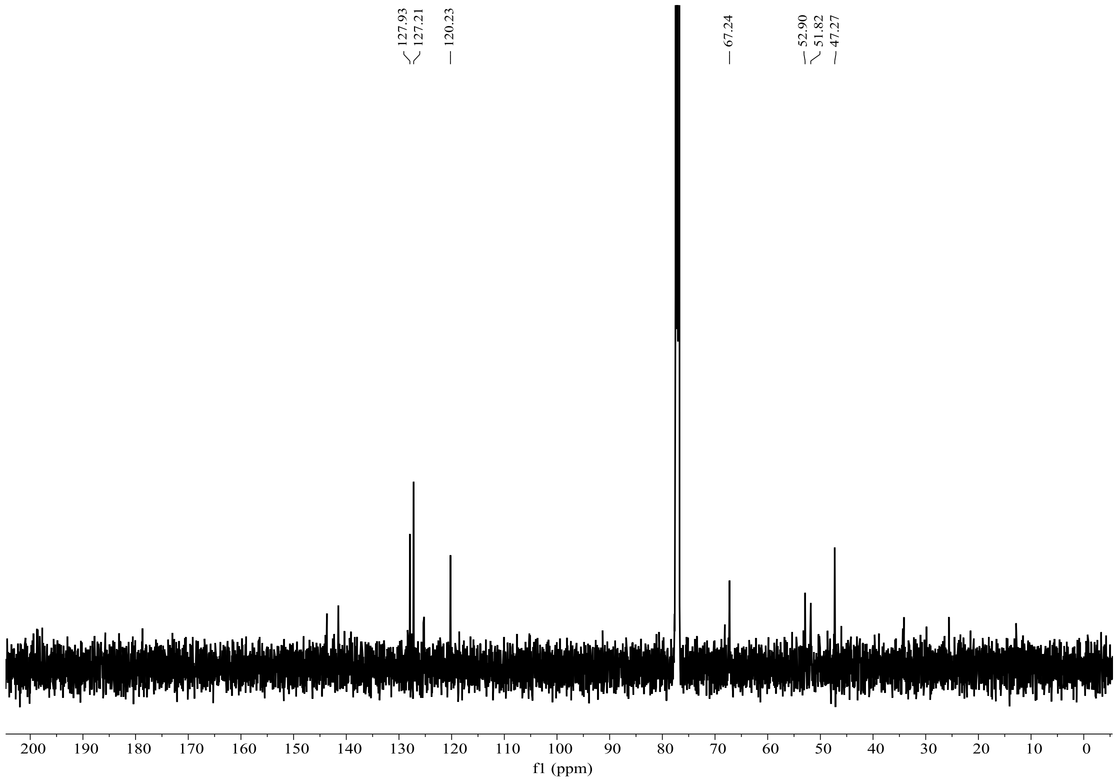

^{19}F{^{1}H} NMR (377 MHz, Chloroform-*d*)

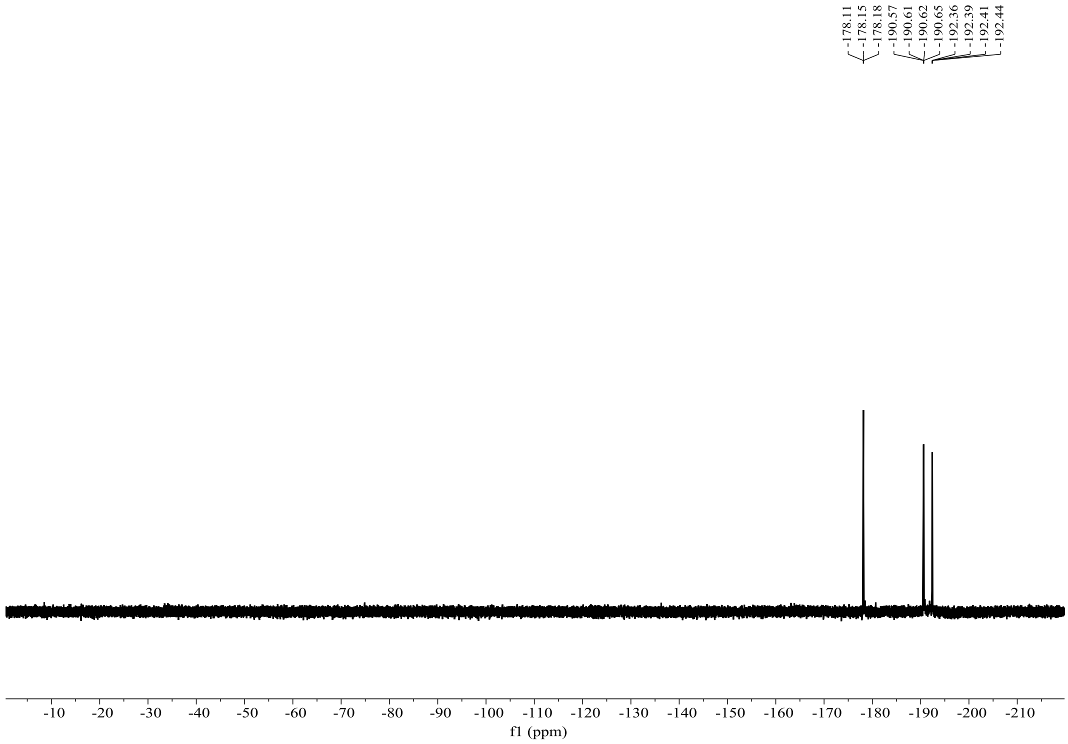

(21d)

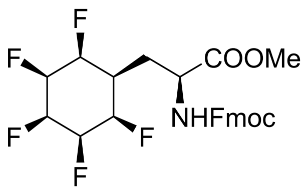

^{1}H NMR (400 MHz, Acetone-d_6)

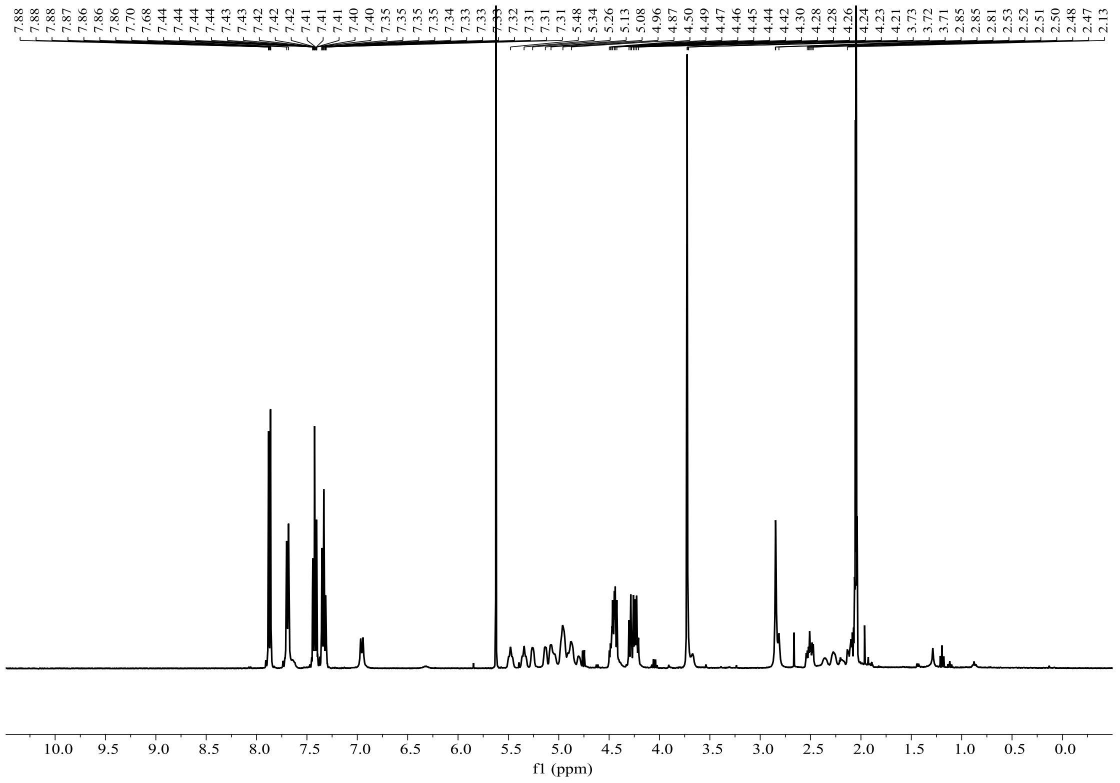

$^{19}F\{^{1}H\}$ NMR (376 MHz, Acetone-d_6)

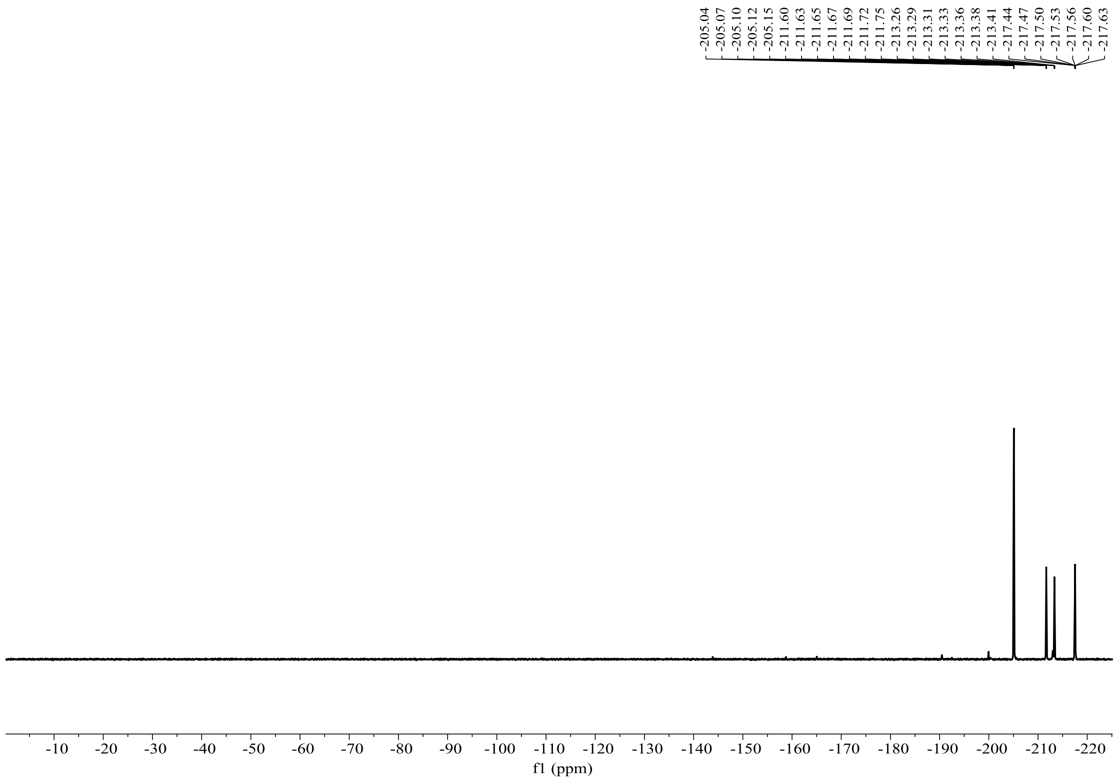

(22a)

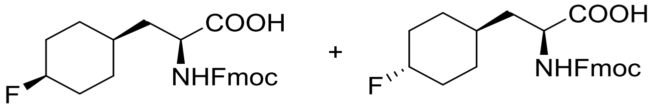

^{1}H NMR (500 MHz, Chloroform-*d*)

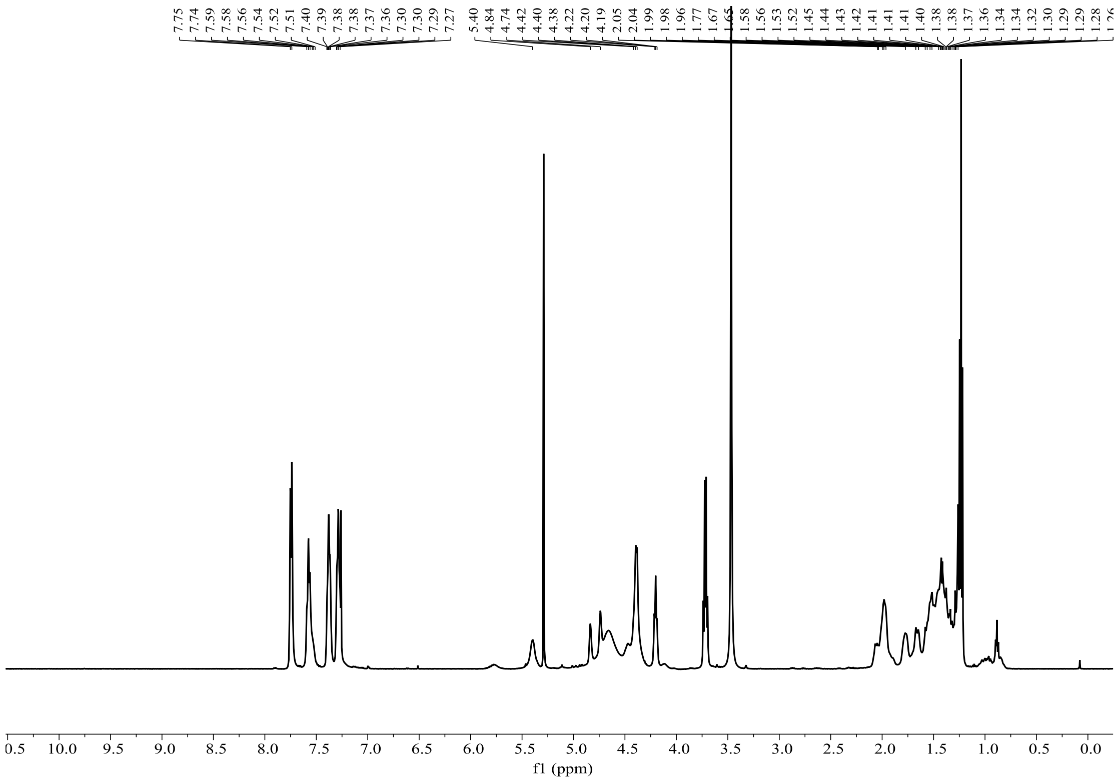

^{13}C NMR (126 MHz, Chloroform-*d*)

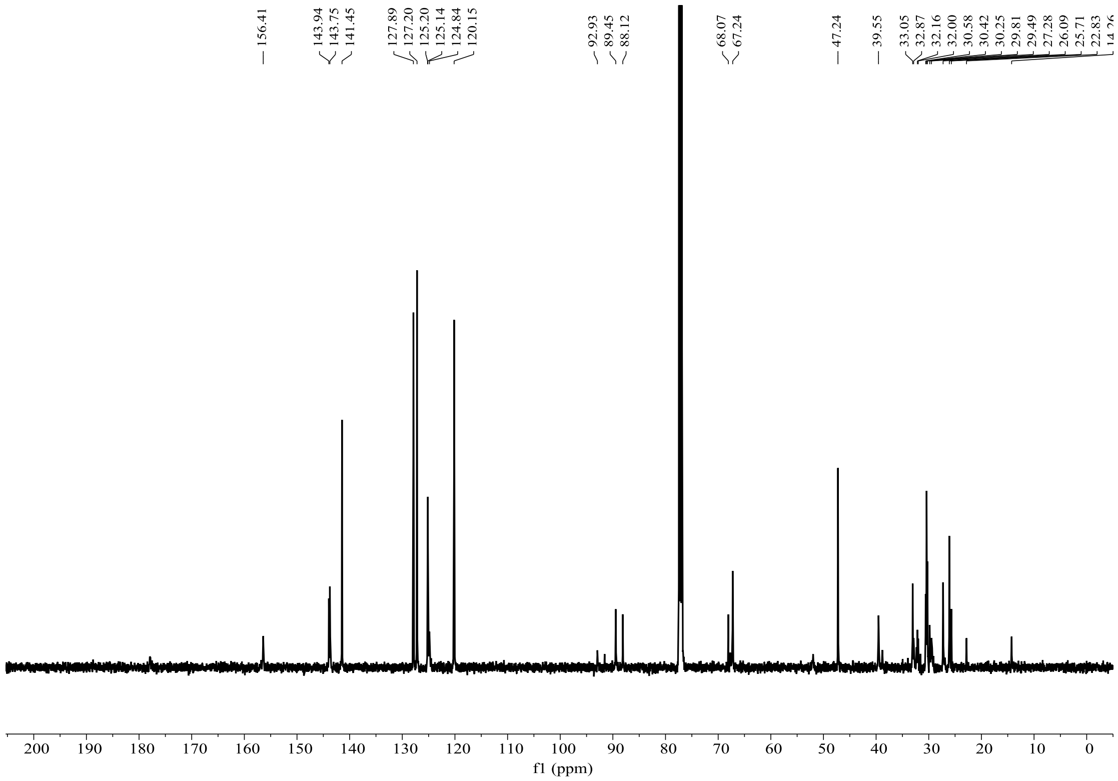

$^{19}F\{^{1}H\}$ NMR (470 MHz, Chloroform-*d*)

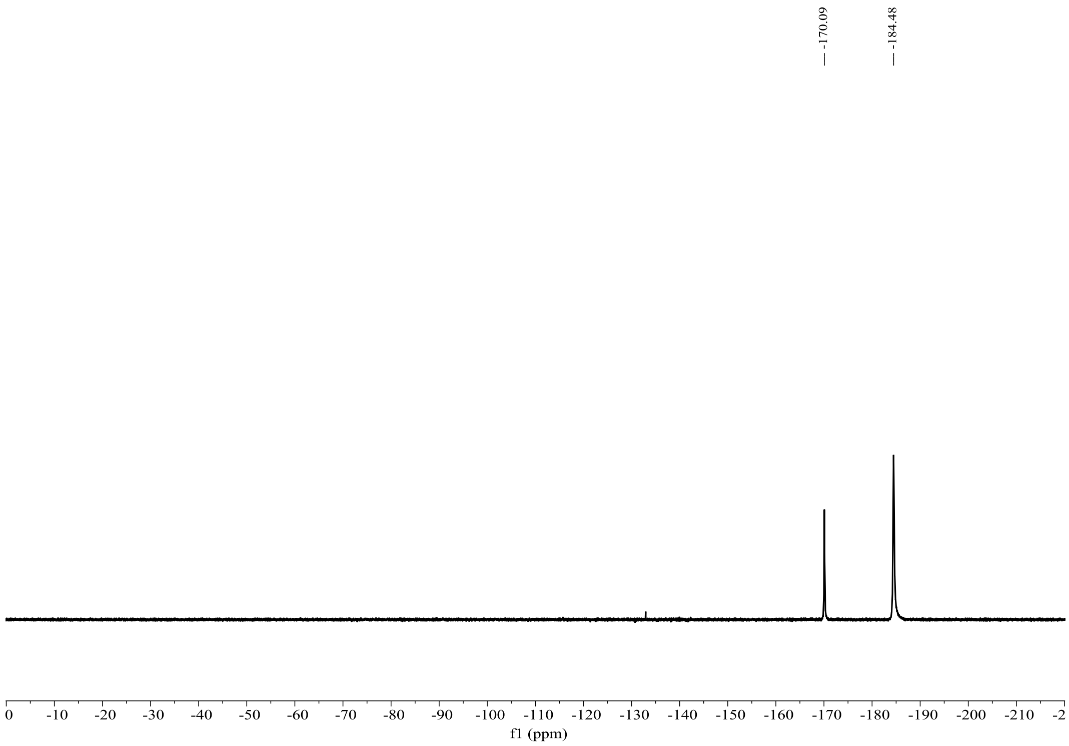

(22b)

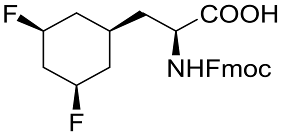

^{1}H NMR (400 MHz, Chloroform-*d*)

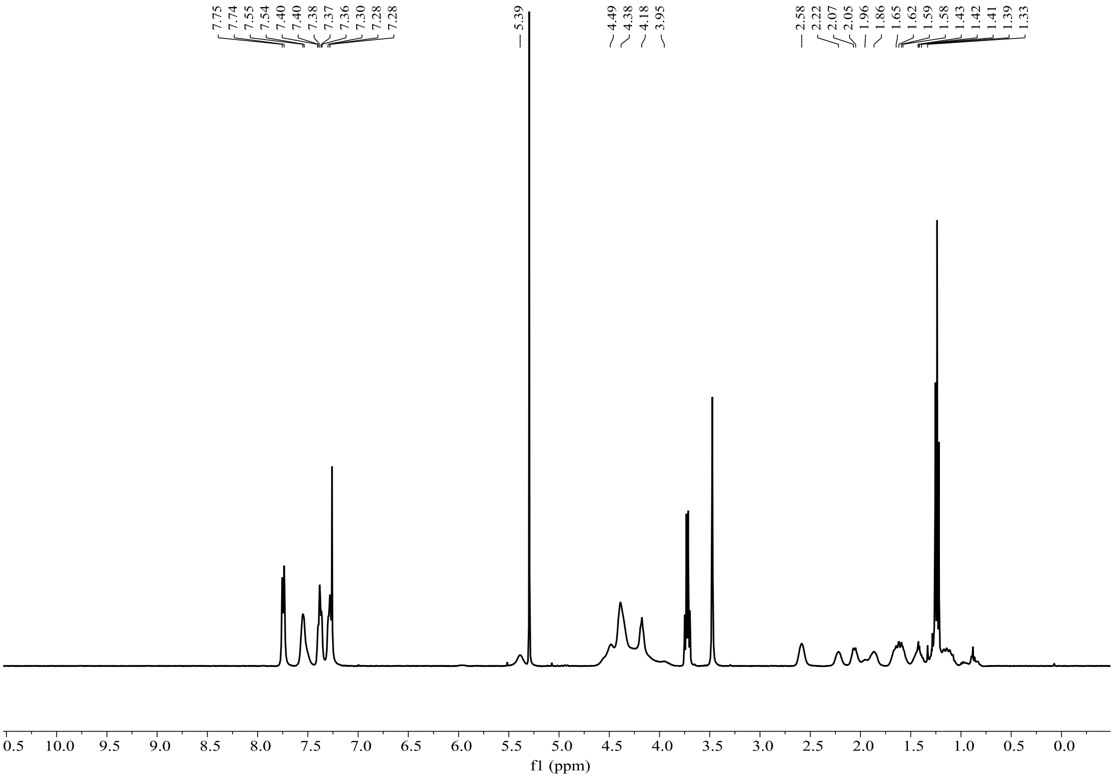

$^{19}F\{^{1}H\}$ NMR (377 MHz, Chloroform-*d*)

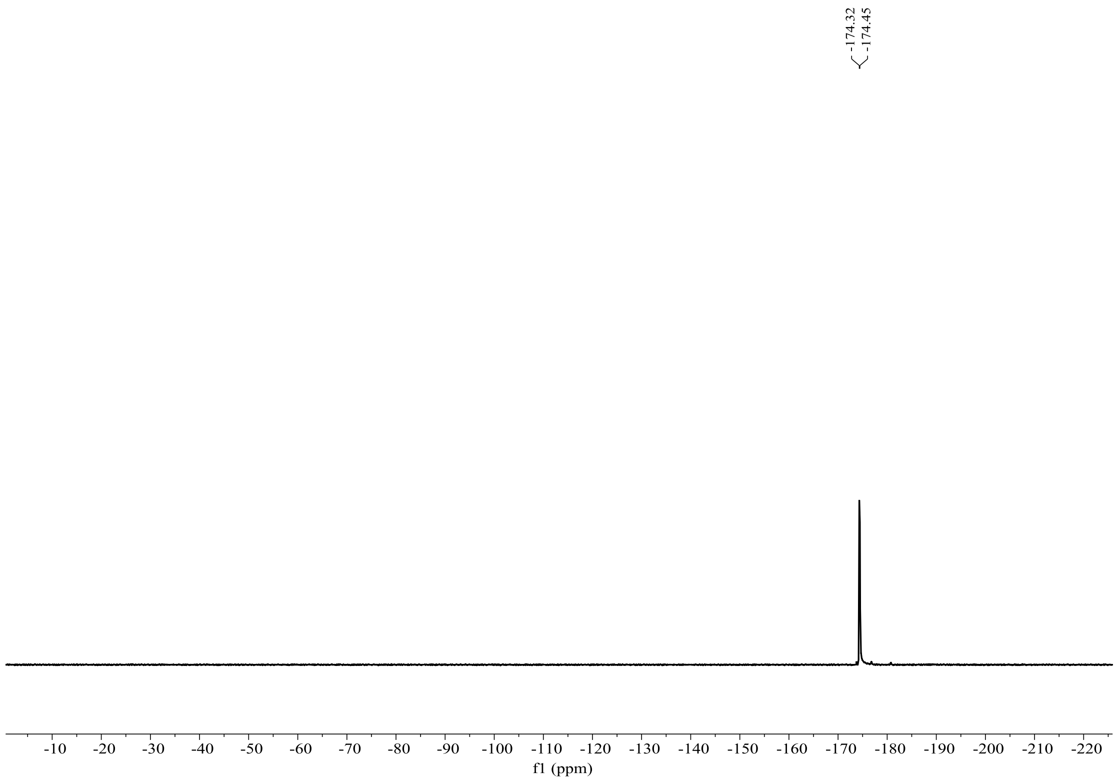

(22c)

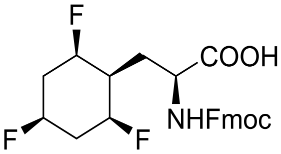

^{1}H NMR (400 MHz, Chloroform-*d*)

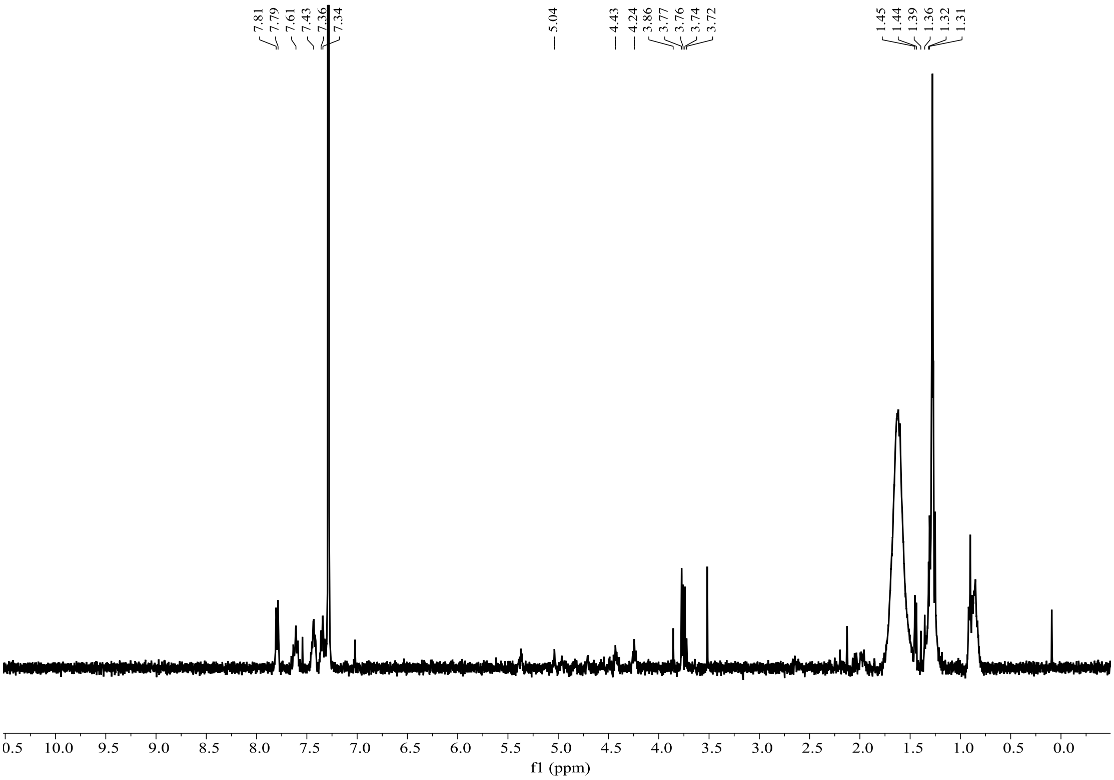

$^{19}F\{^1H\}$ NMR (376 MHz, Chloroform-*d*)

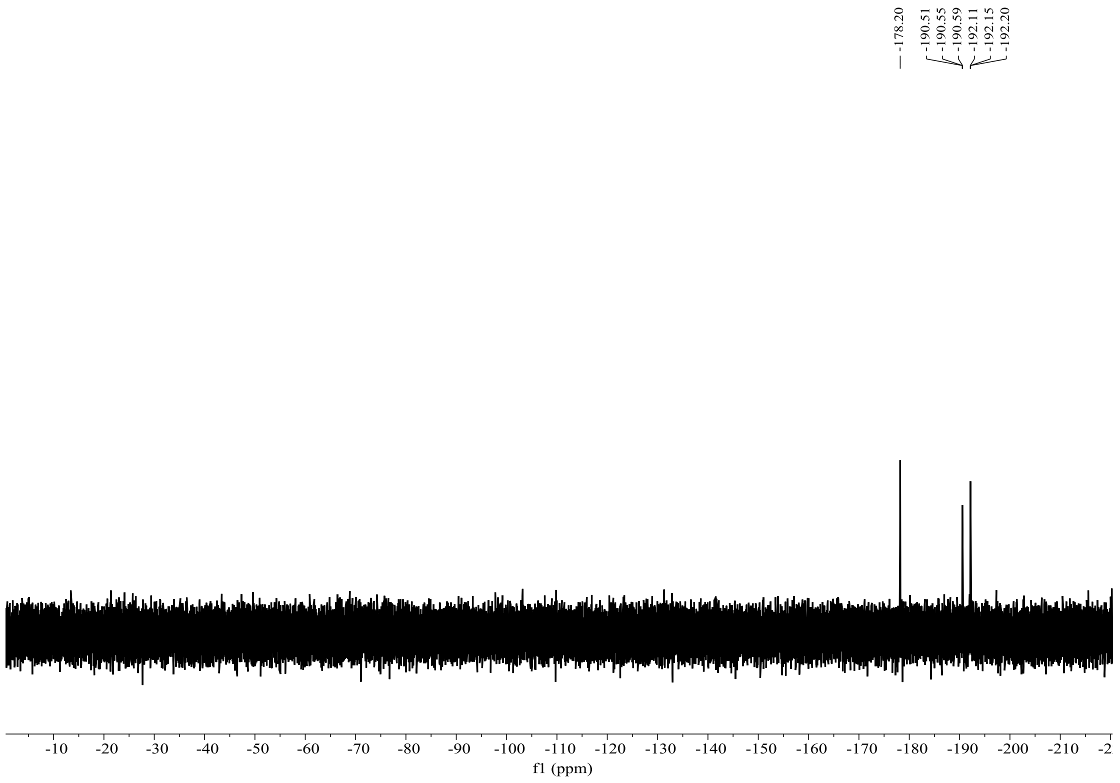

(22d)

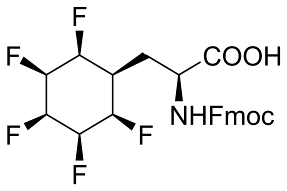

^{1}H NMR (400 MHz, Acetone-d_6)

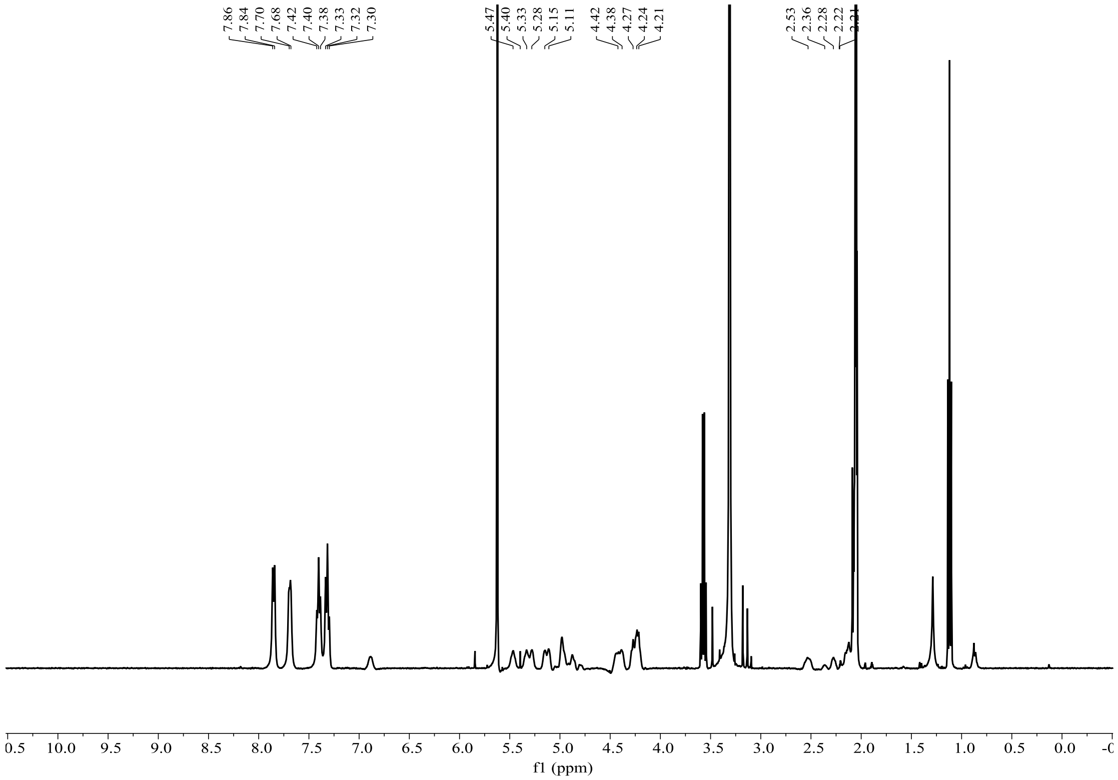

$^{19}F\{^1H\}$ NMR (376 MHz, Acetone-d_6)

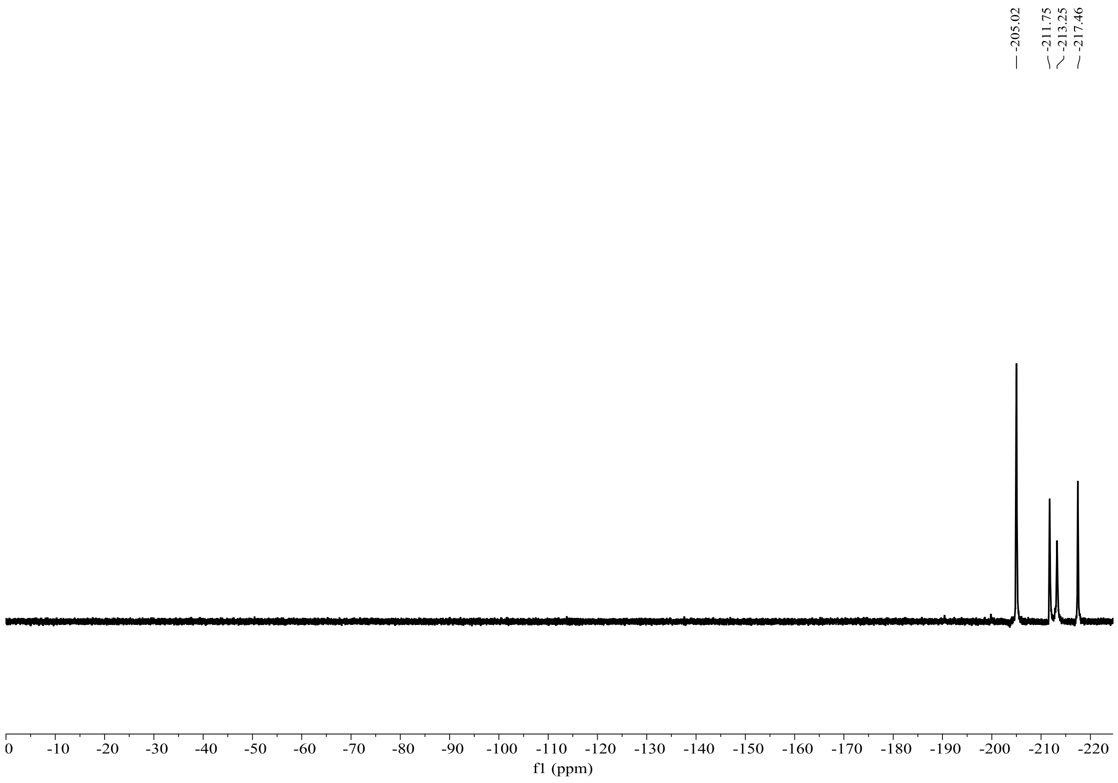

(26a)

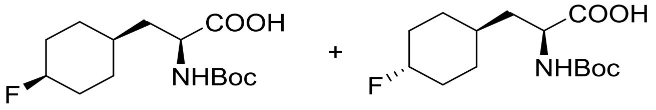

^{1}H NMR (500 MHz, Chloroform-*d*)

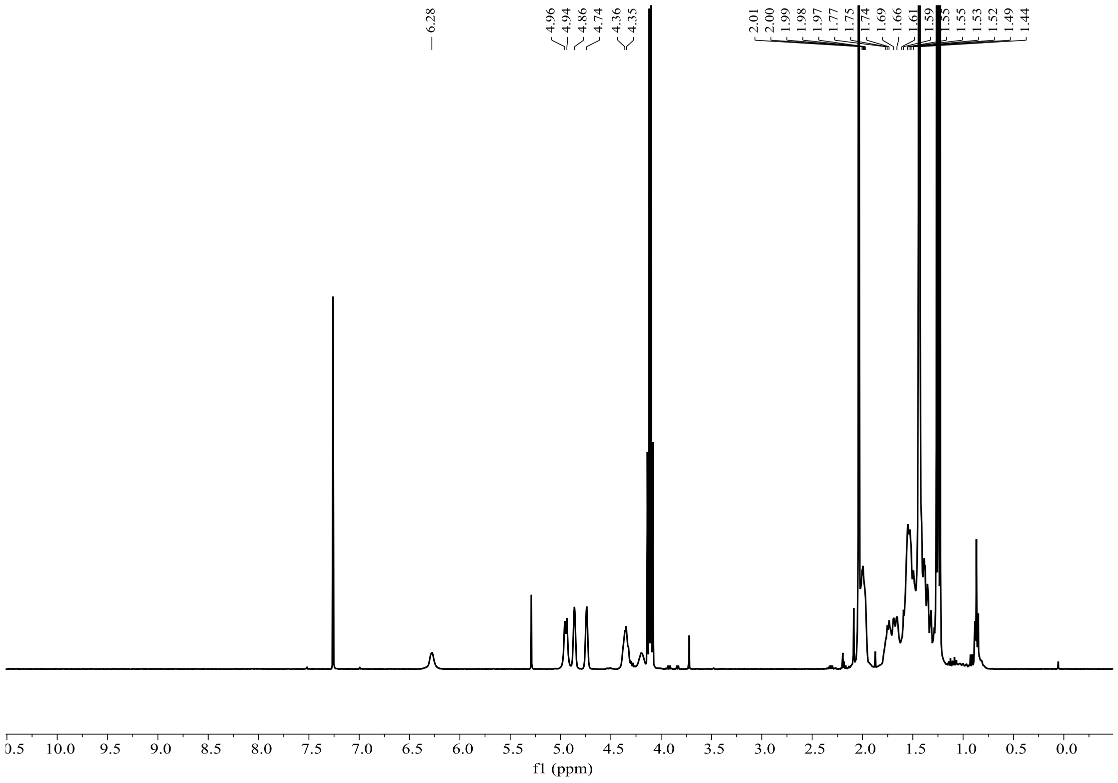

^{13}C NMR (126 MHz, Chloroform-*d*)

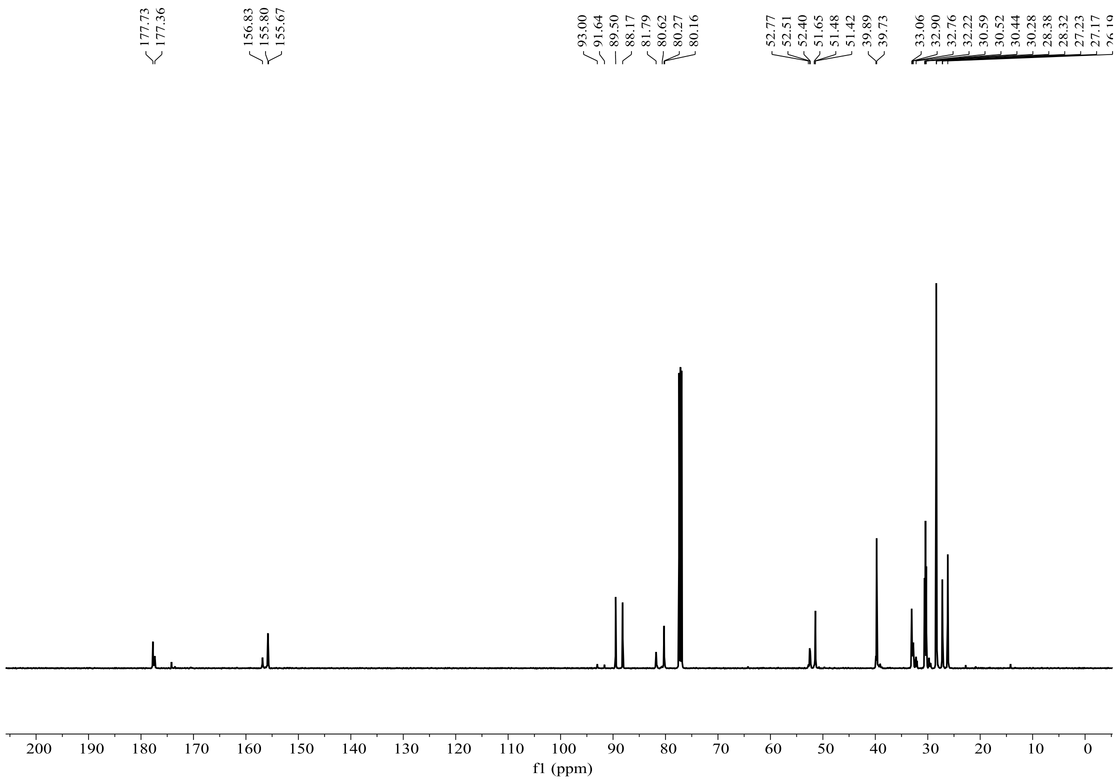

$^{19}F\{^{1}H\}$ NMR (376 MHz, Chloroform-*d*)

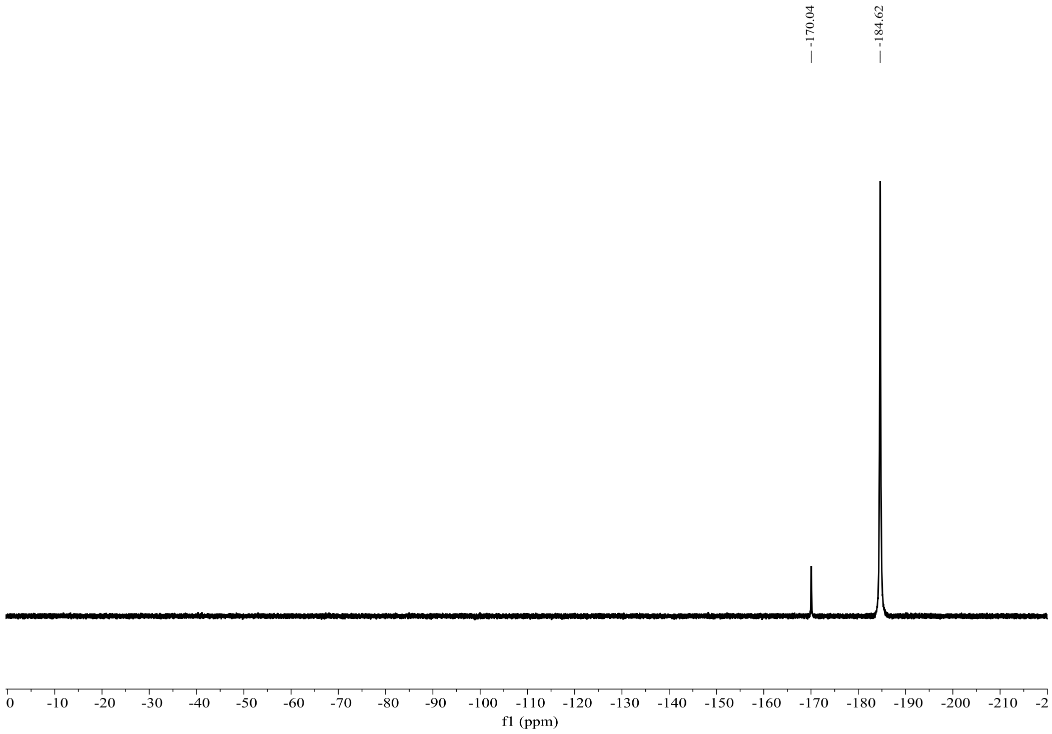

(26b)

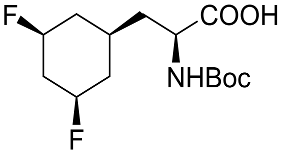

^{1}H NMR (500 MHz, Chloroform-*d*)

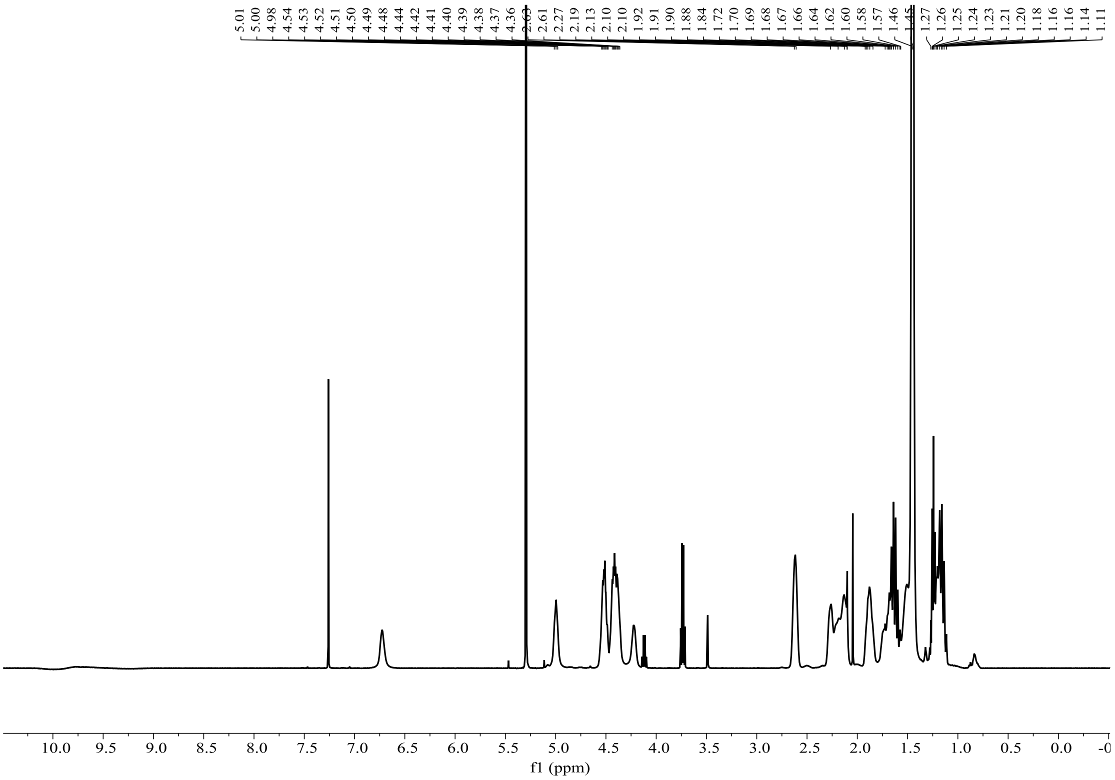

^{13}C NMR (126 MHz, Chloroform-*d*)

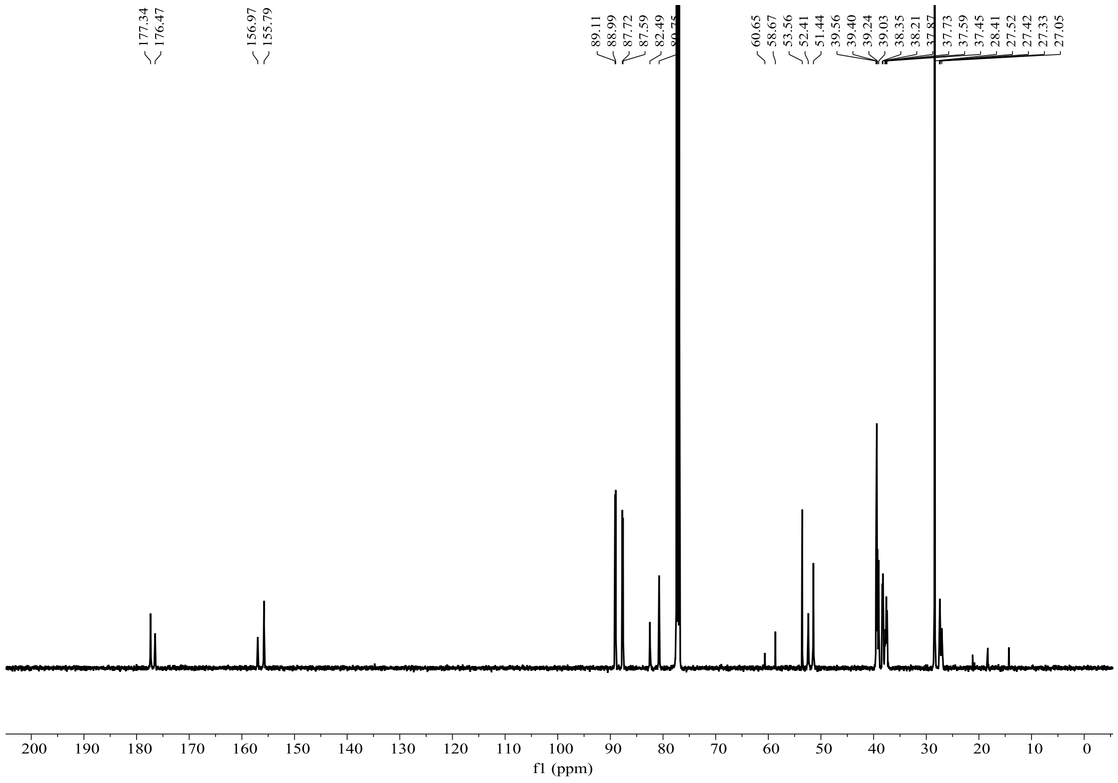

$^{19}F\{^{1}H\}$ NMR (377 MHz, Chloroform-*d*)

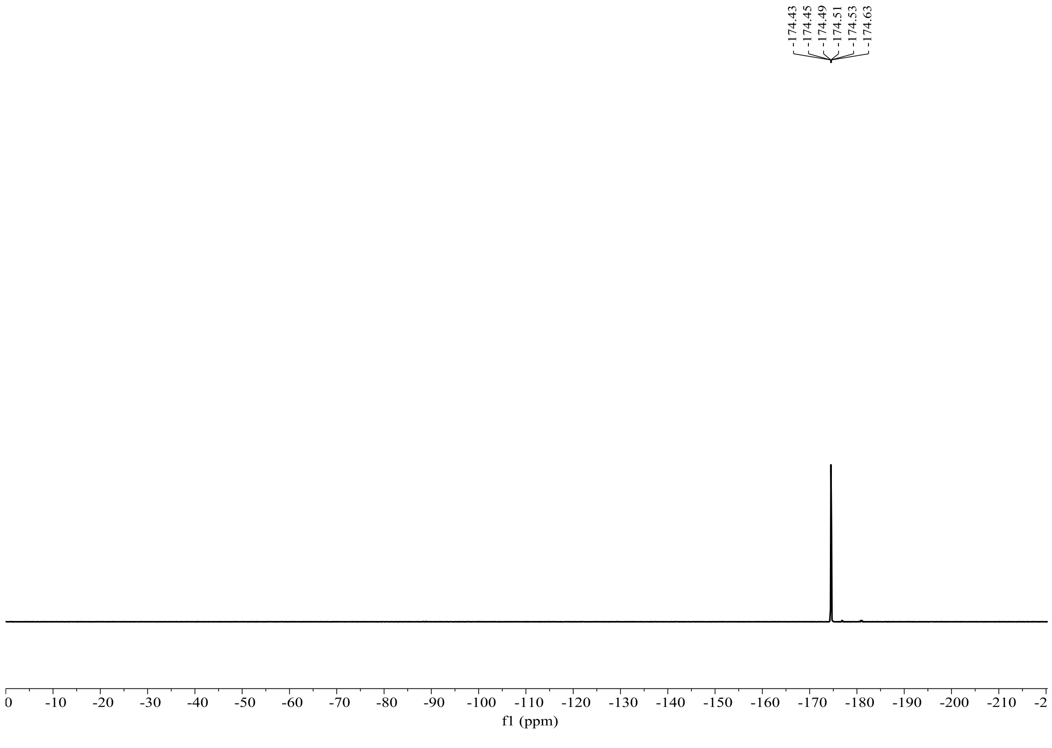

(26c)

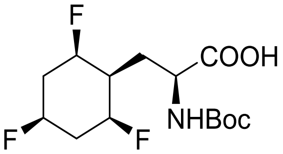

^{1}H NMR (500 MHz, Methanol-d_4)

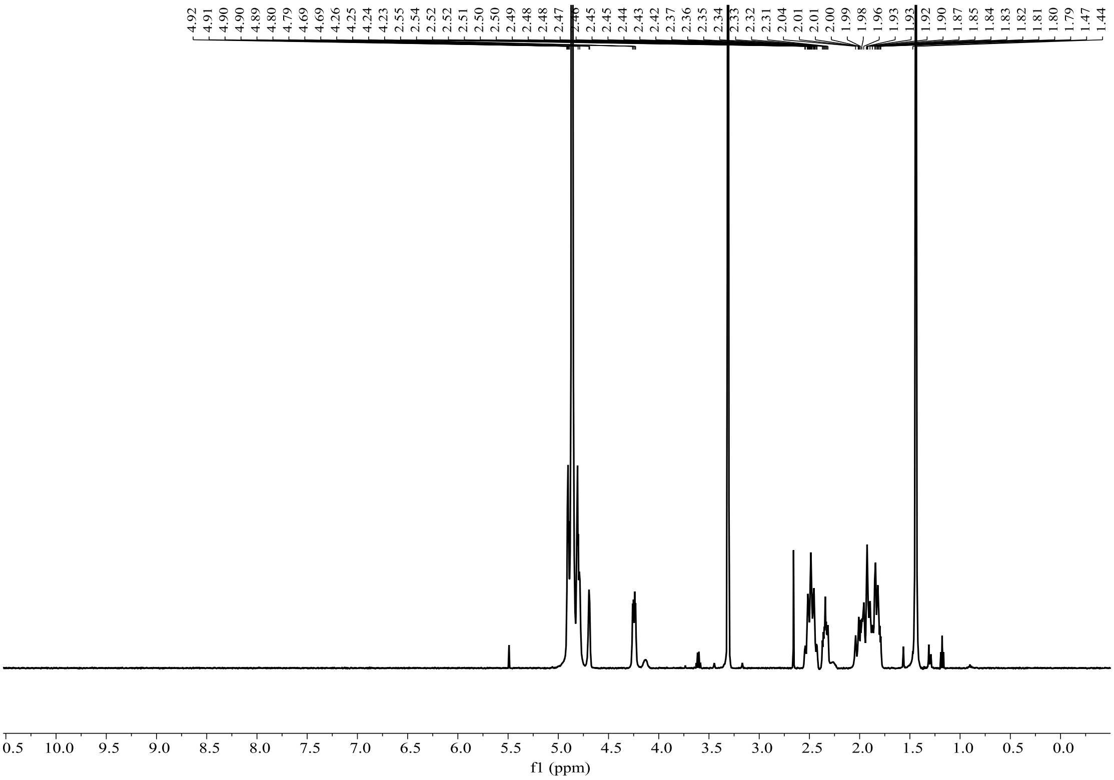

^{13}C NMR (126 MHz, Methanol-d_4)

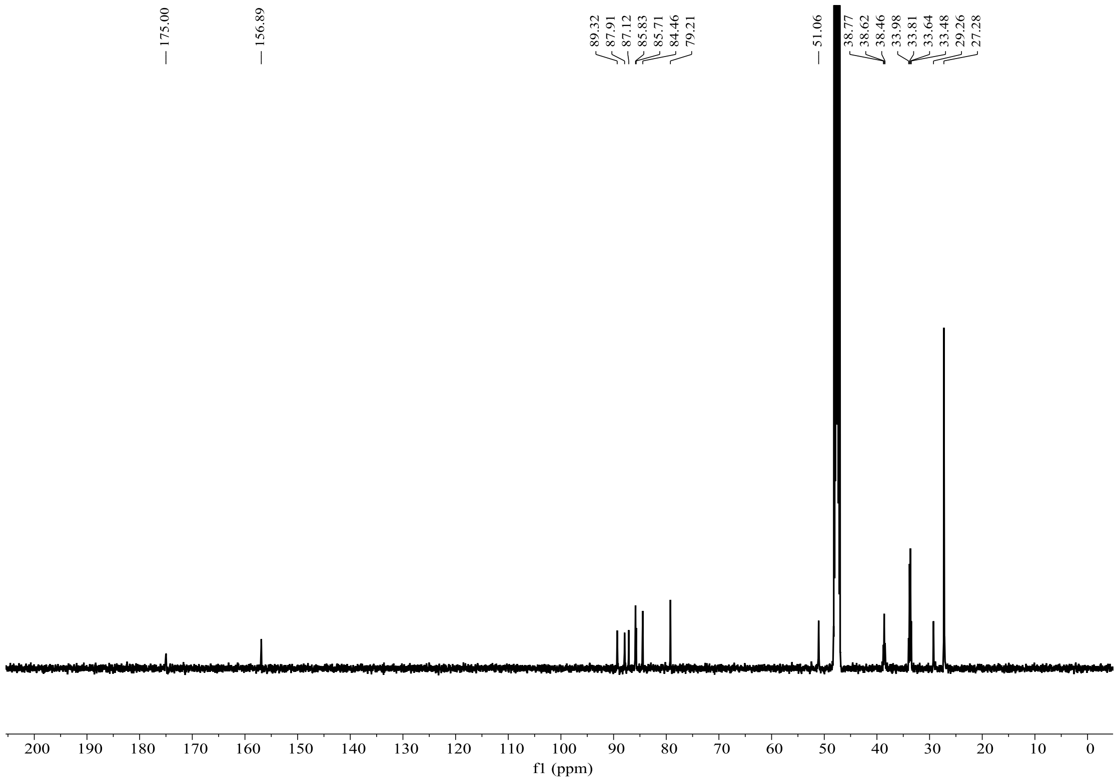

^{19}F{^{1}H} NMR (377 MHz, Methanol-d_4)

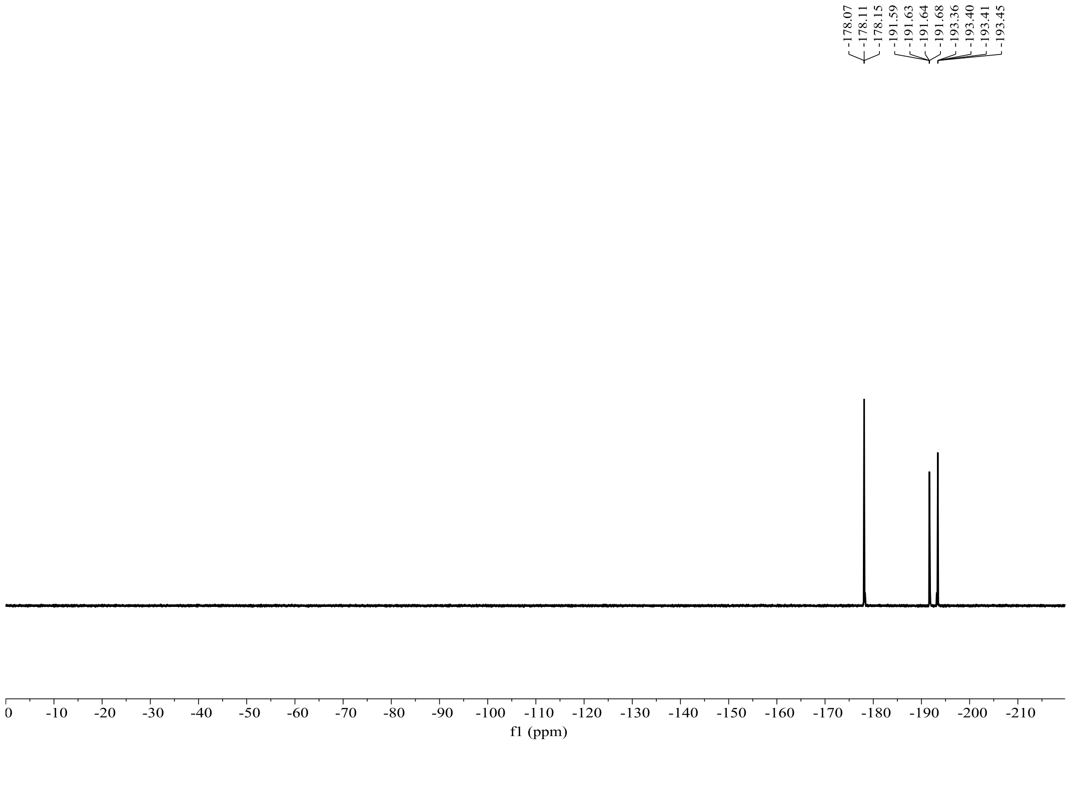

(26d)

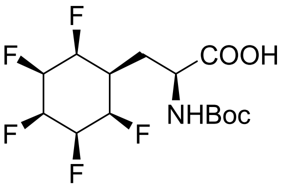

^{1}H NMR (500 MHz, Methanol-d_4)

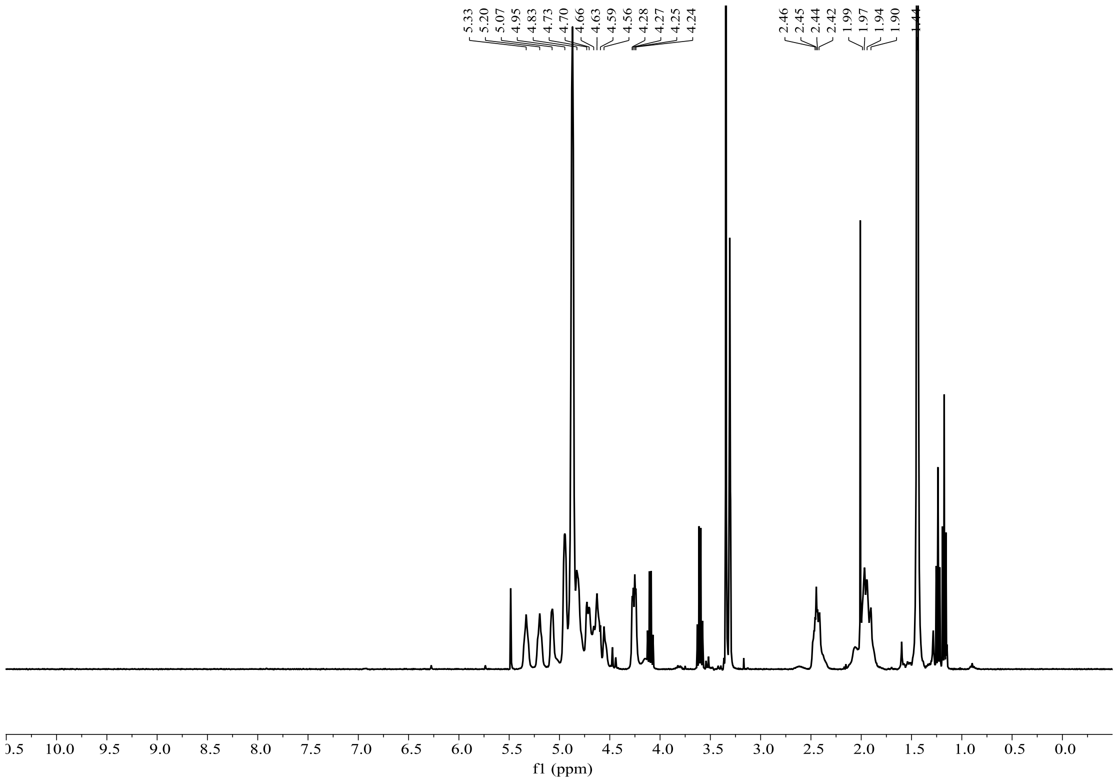

$^{19}F\{^{1}H\}$ NMR (377 MHz, Methanol-d_4)

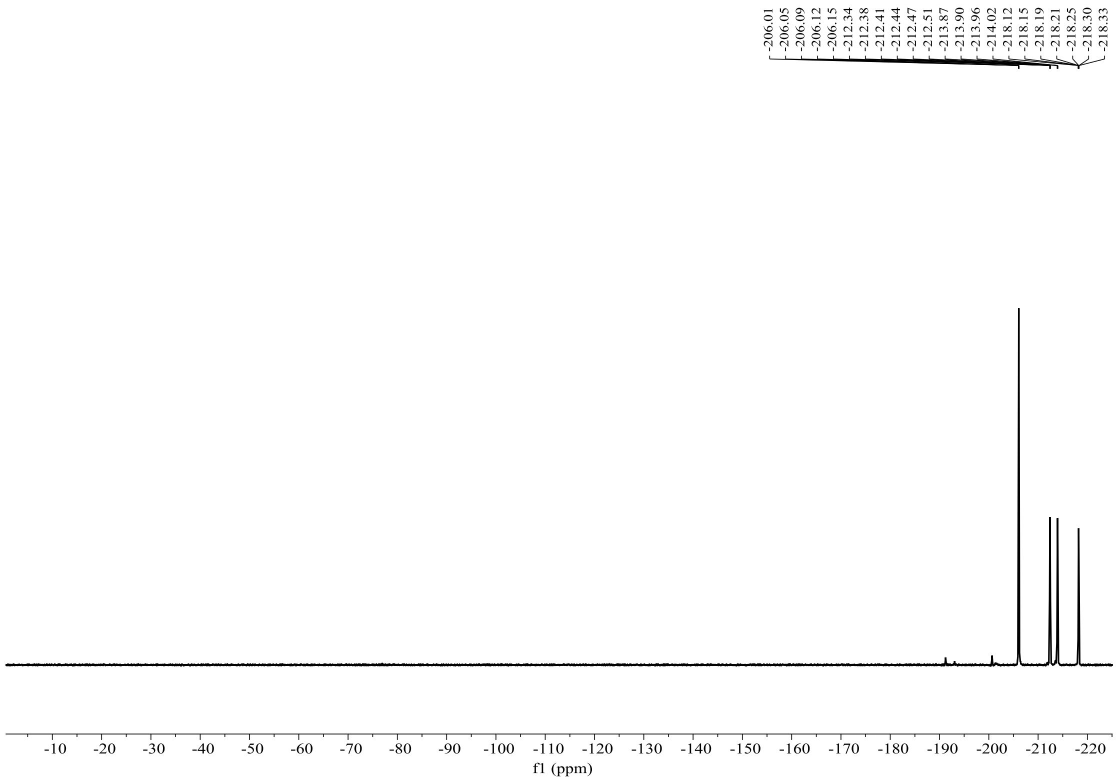

(27a)

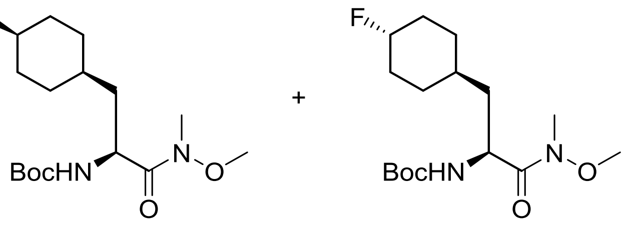

^{1}H NMR (400 MHz, Chloroform-*d*)

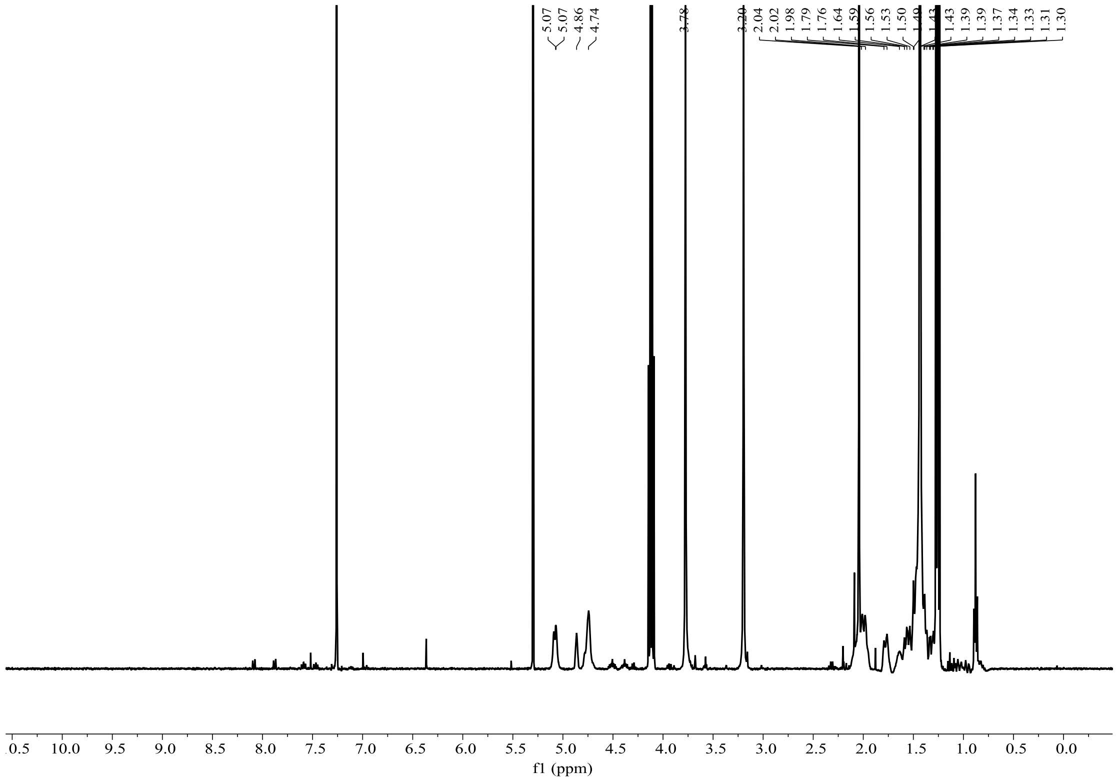

^{13}C NMR (126 MHz, Methanol-d_4)

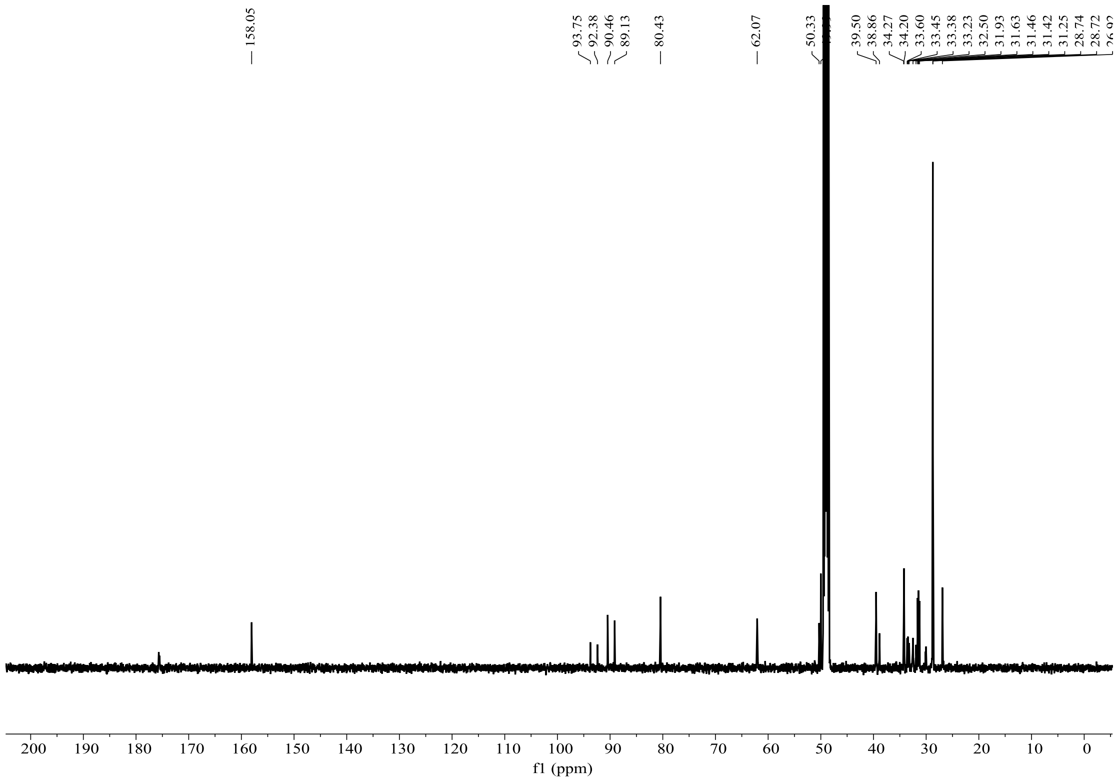

$^{19}F\{^1H\}$ NMR (376 MHz, Chloroform-d)

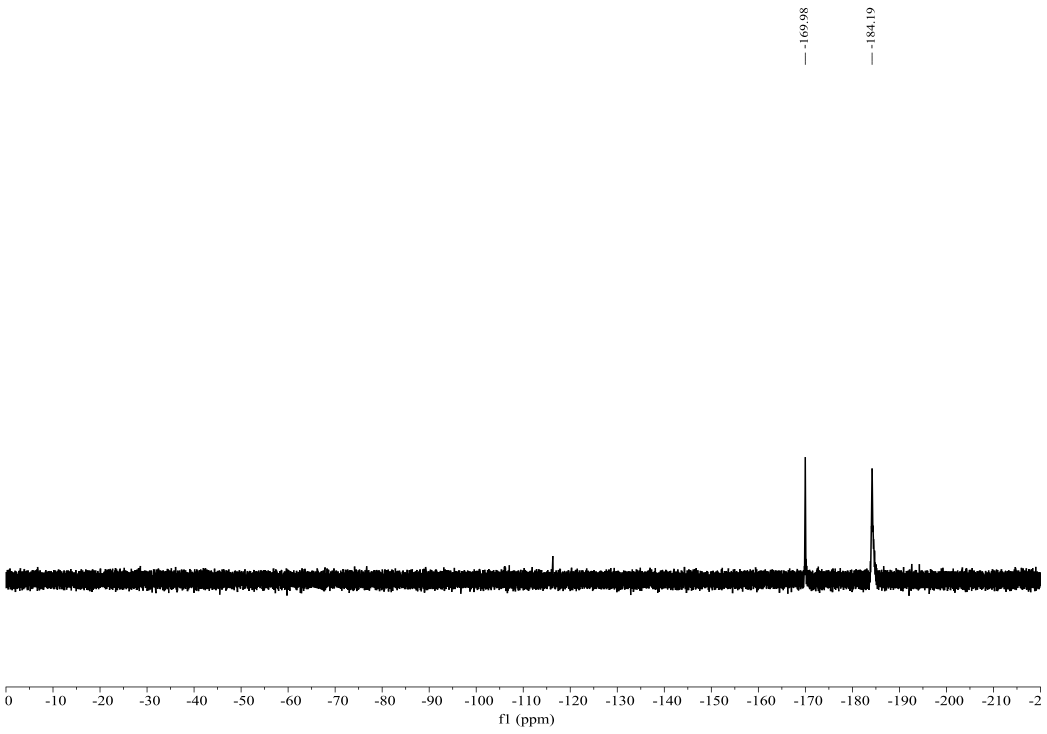

(27b)

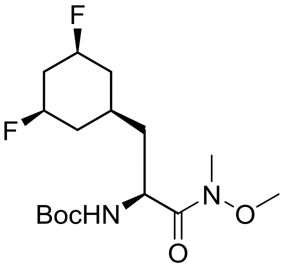

^{1}H NMR (400 MHz, Methanol-d_4)

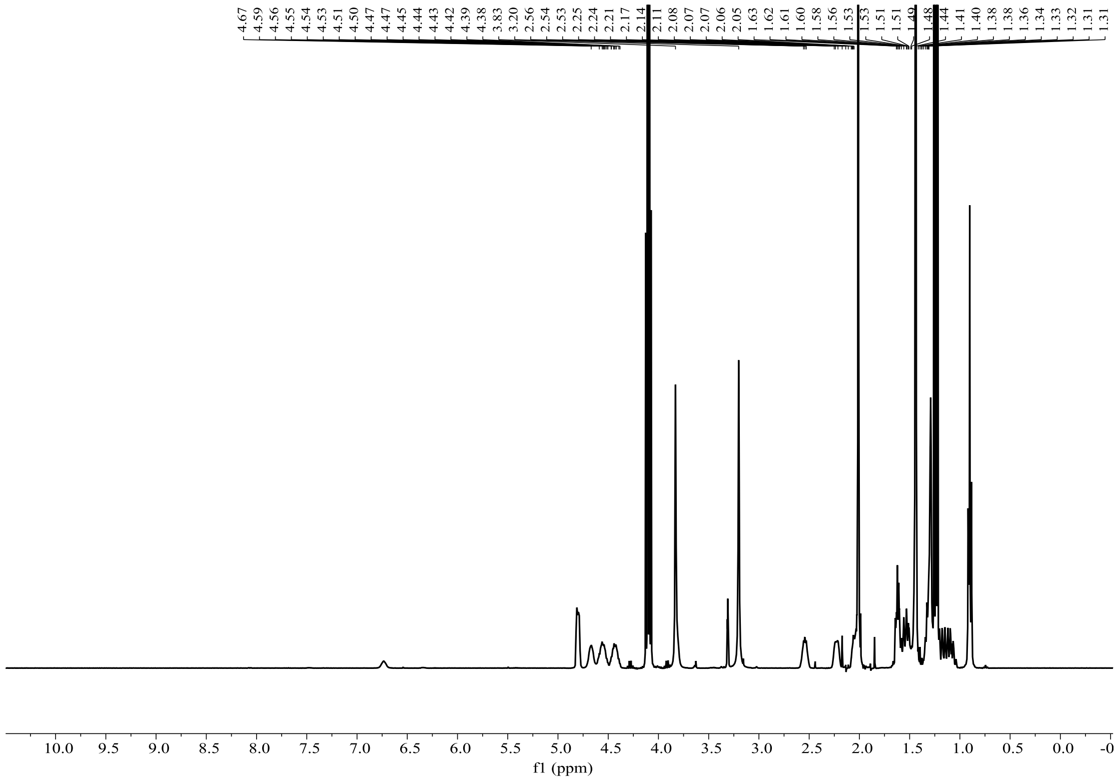

^{13}C NMR (126 MHz, Methanol-d_4)

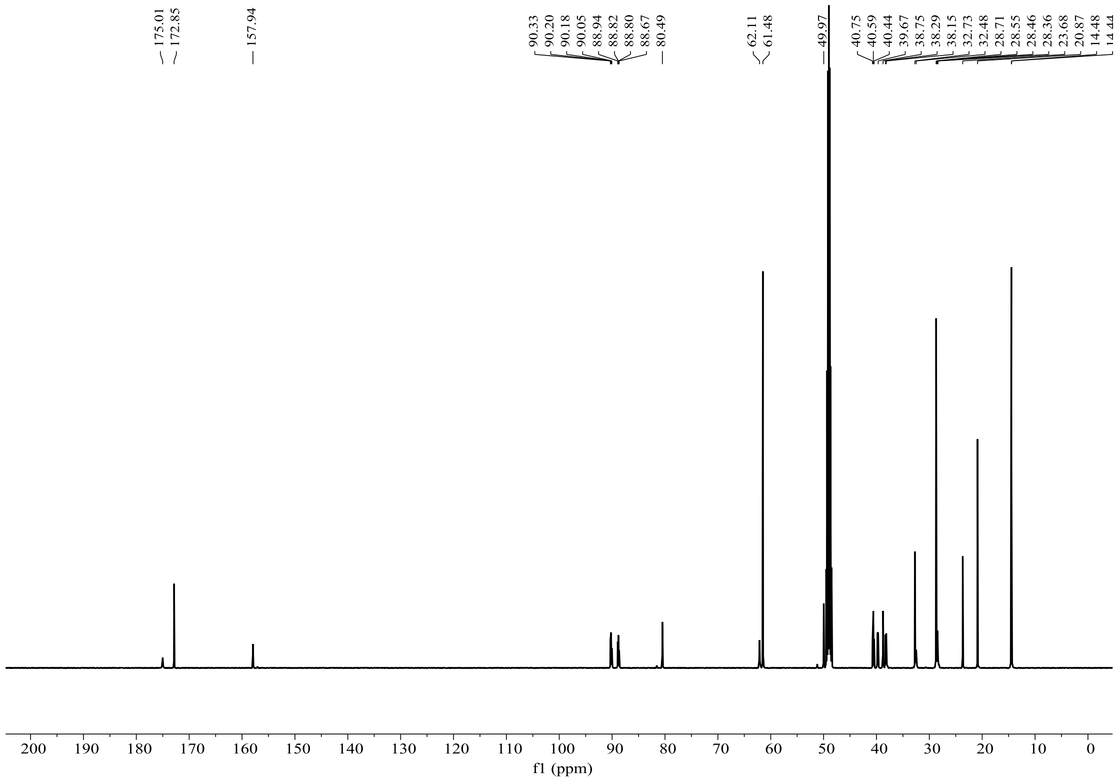

$^{19}F\{^1H\}$ NMR (377 MHz, Methanol-d_4)

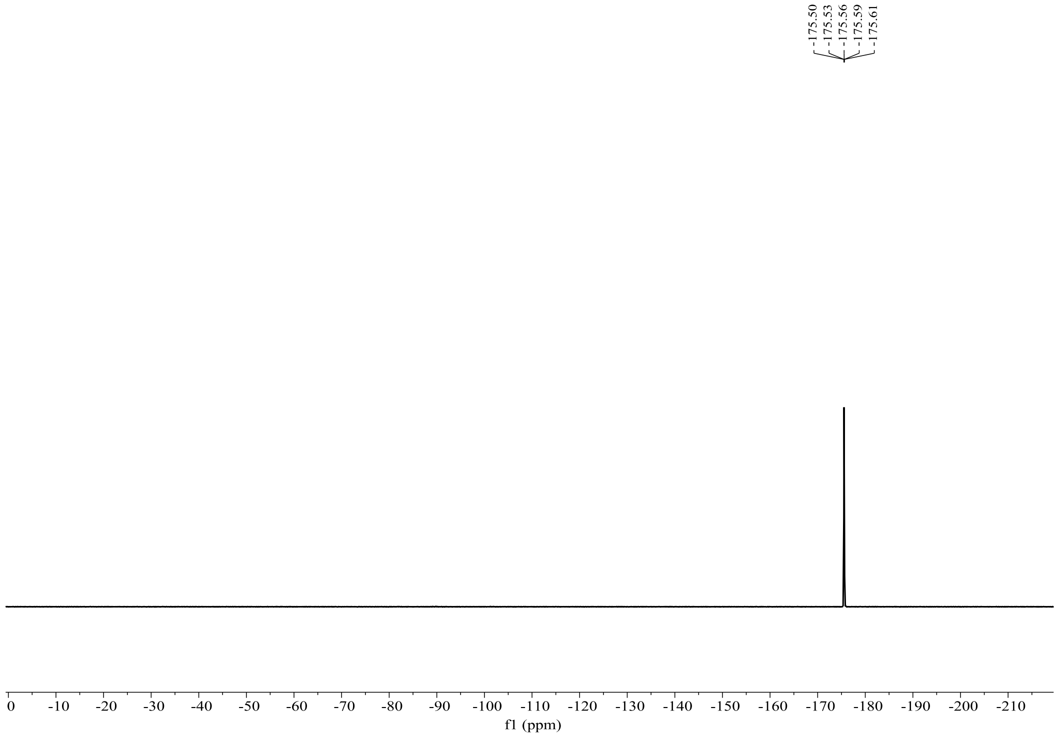

(27c)

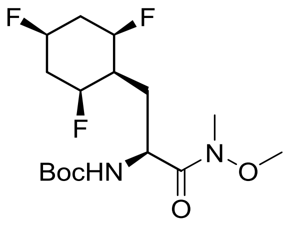

^{1}H NMR (400 MHz, Methanol-d_4)

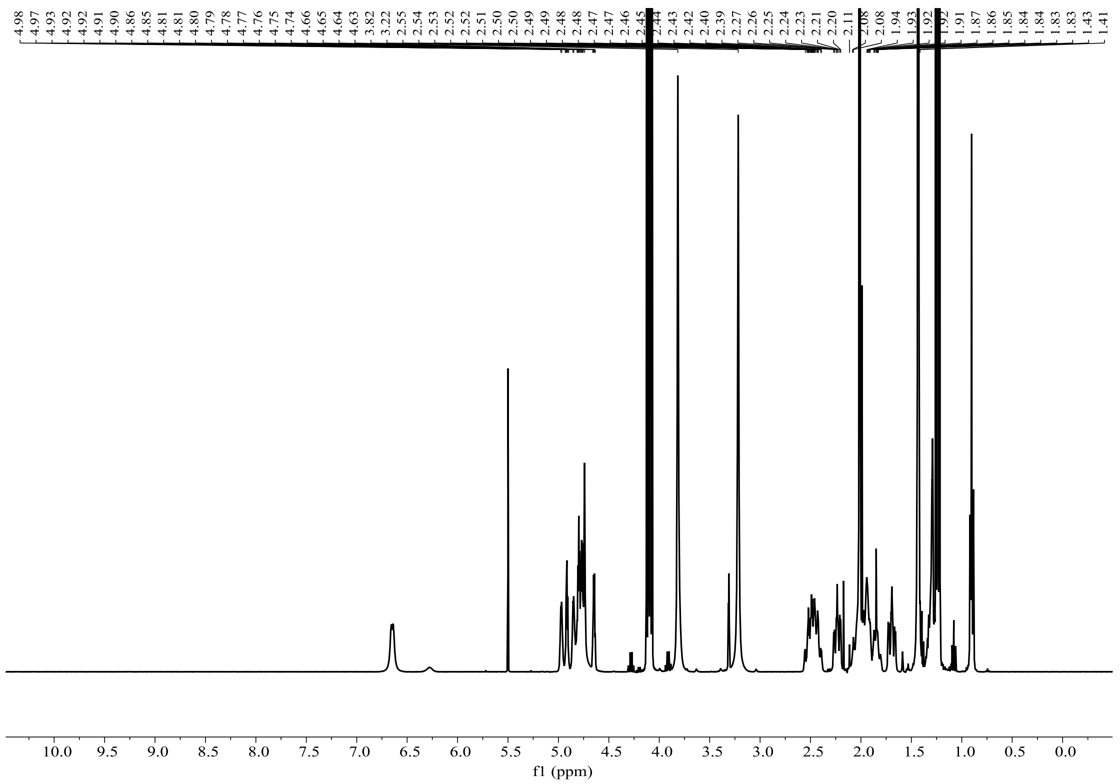

^{13}C NMR (126 MHz, Methanol-d_4)

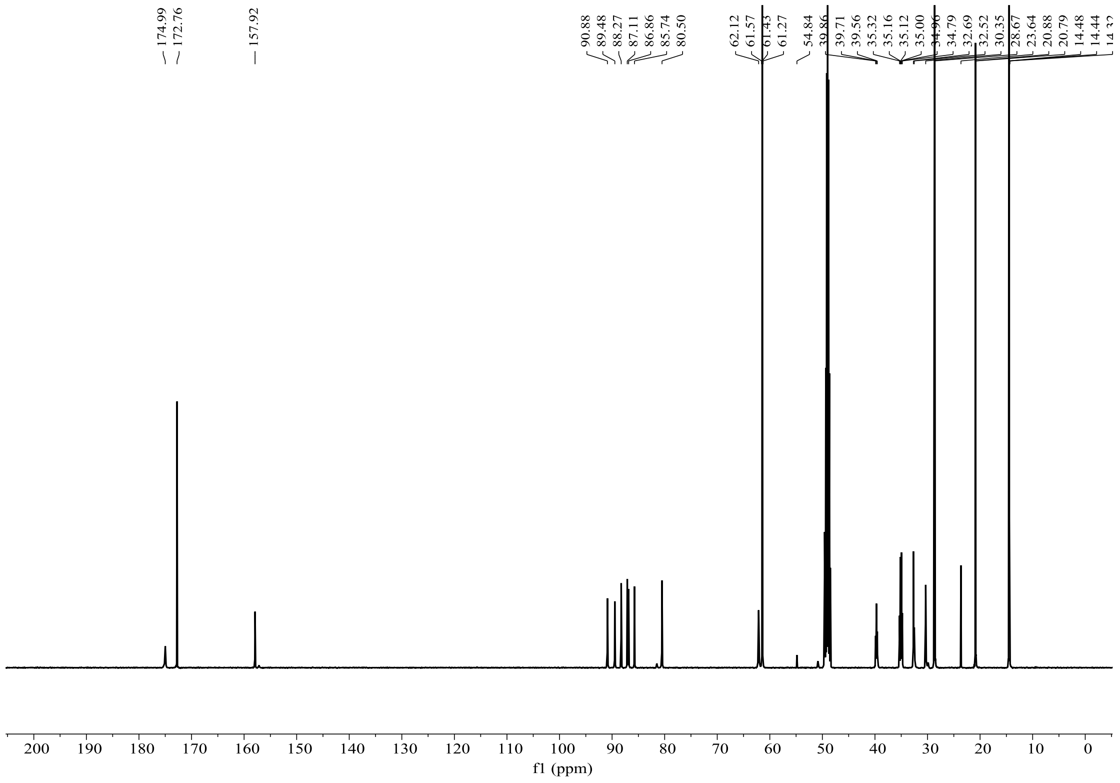

$^{19}F\{^1H\}$ NMR (377 MHz, Methanol-d_4)

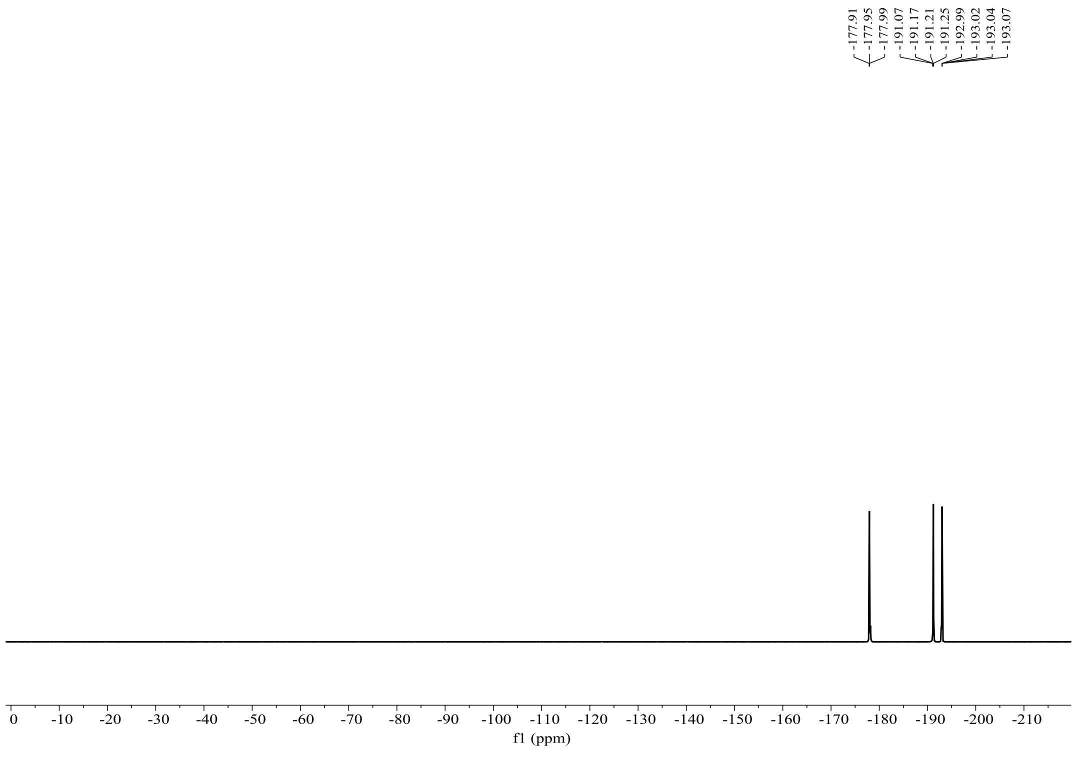

(27d)

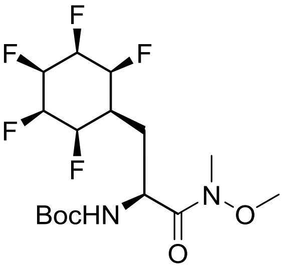

^{1}H NMR (400 MHz, Methanol-d_4)

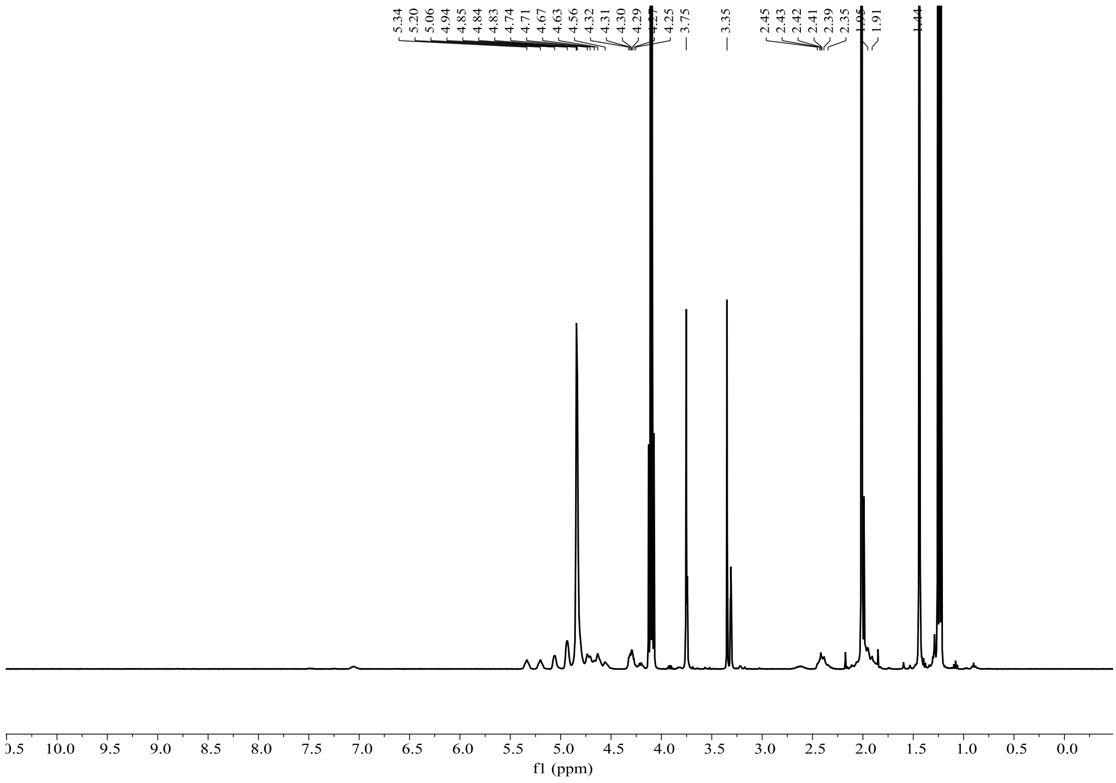

$^{19}F\{^{1}H\}$ NMR (377 MHz, Methanol-d_4)

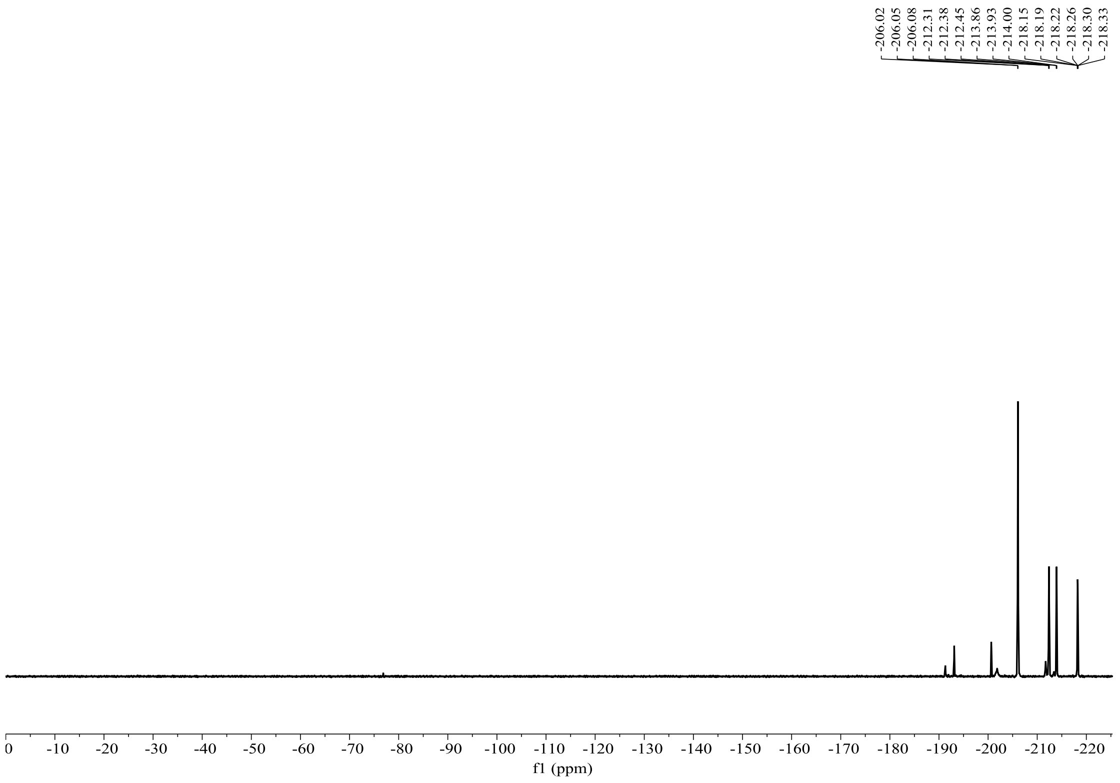

(28a)

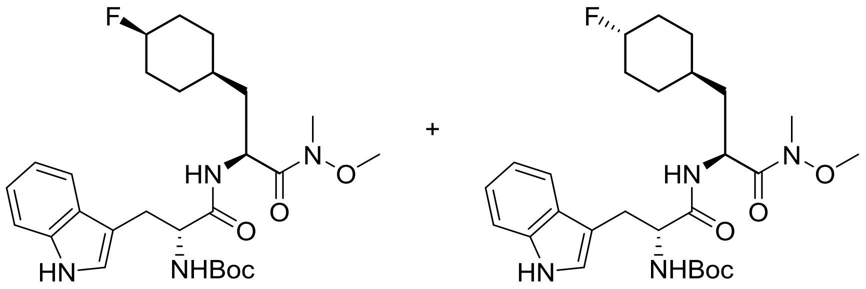

^{1}H NMR (400 MHz, Chloroform-*d*)

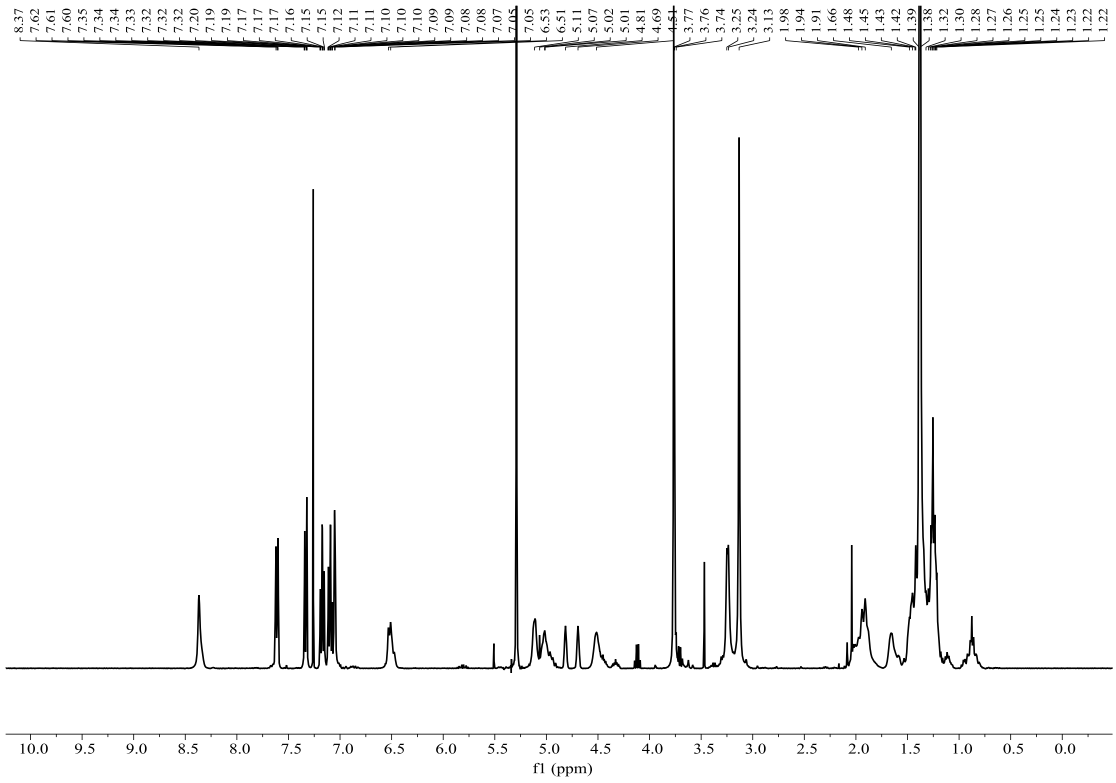

^{13}C NMR (126 MHz, Chloroform-*d*)

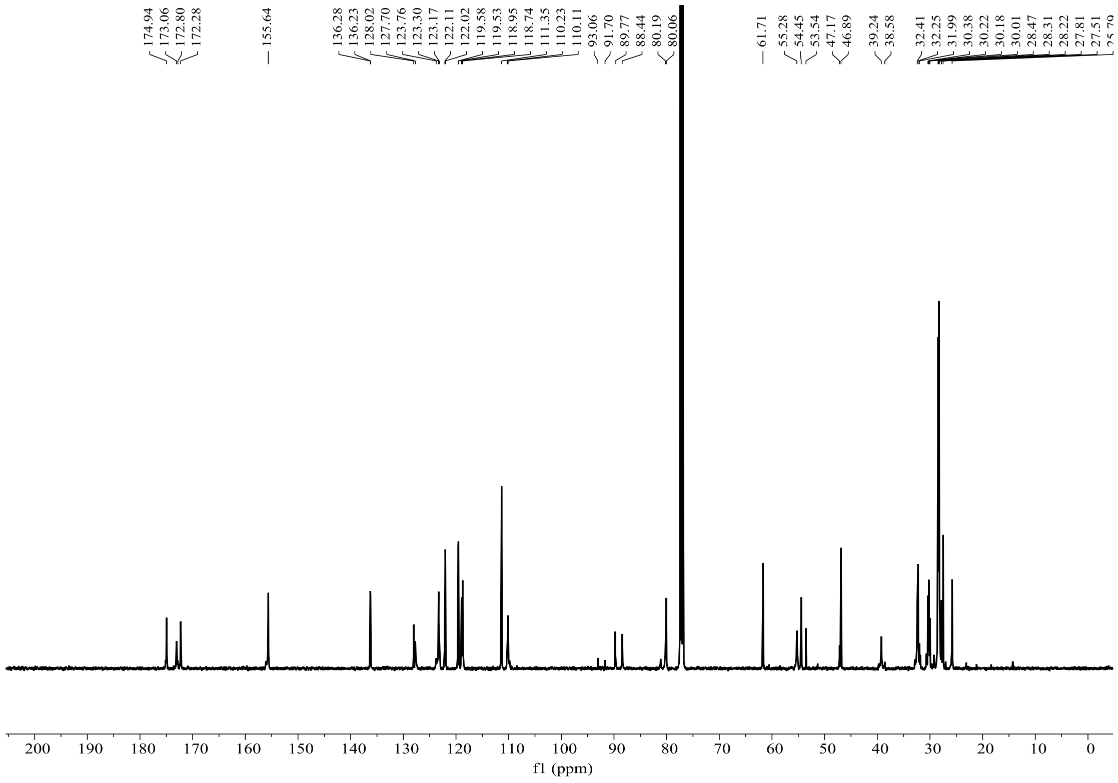

^{19}F{^{1}H} NMR (377 MHz, Chloroform-*d*)

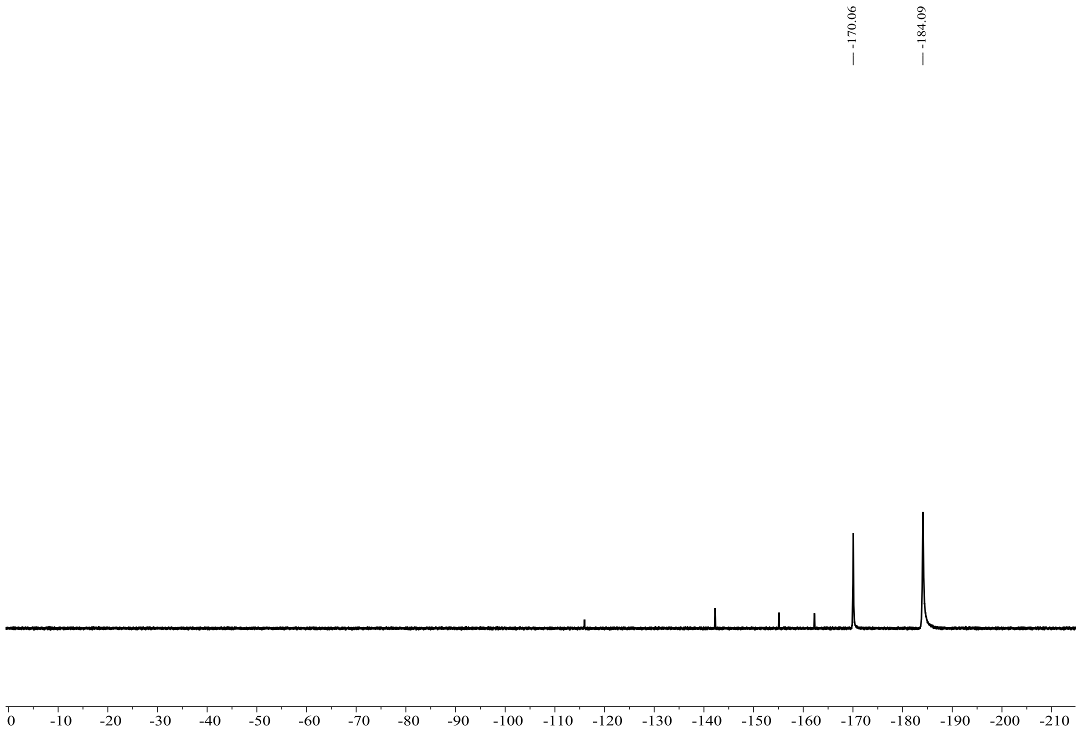

(28b)

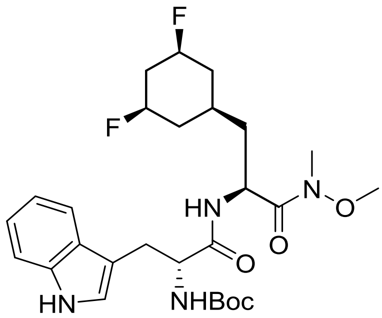

^{1}H NMR (400 MHz, Methanol-d_4)

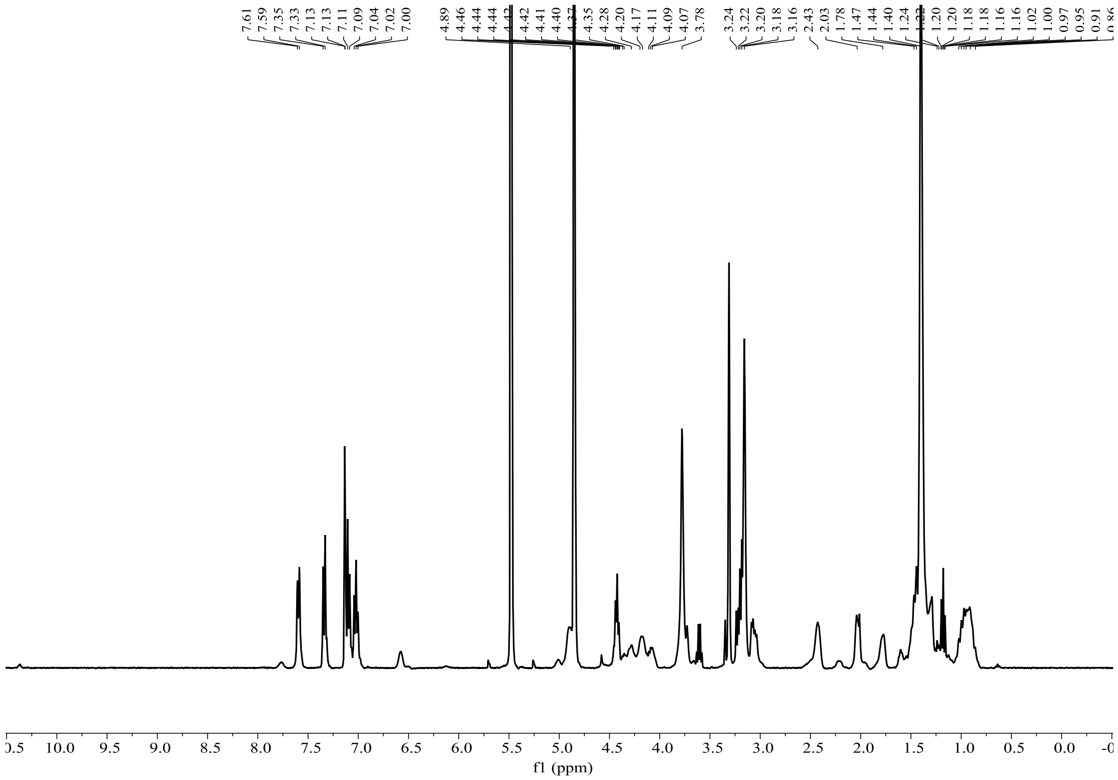

^{13}C NMR (126 MHz, Methanol-d_4)

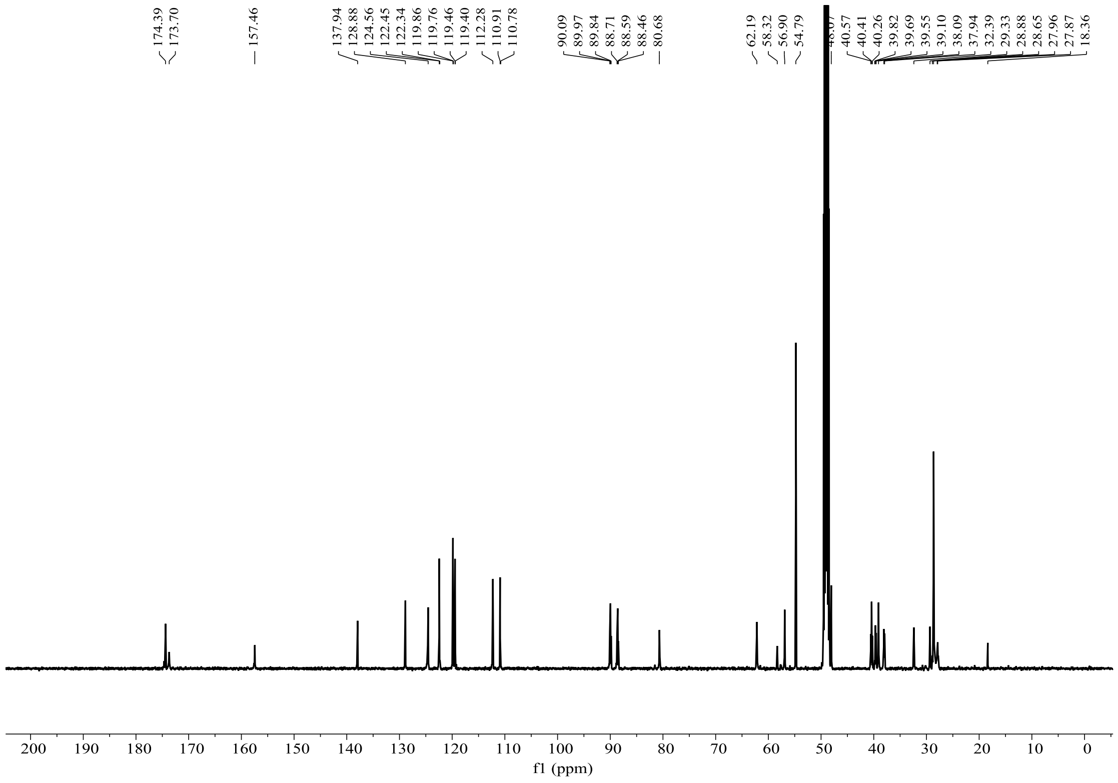

$^{19}F\{^1H\}$ NMR (377 MHz, Methanol-d_4)

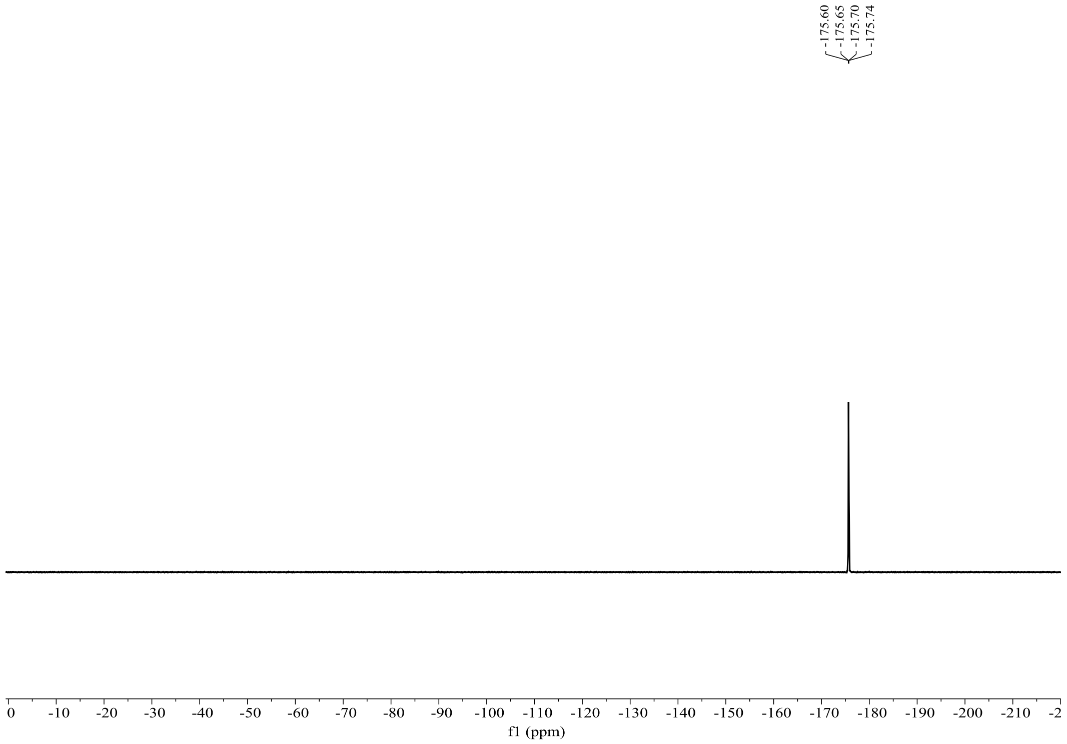

(28c)

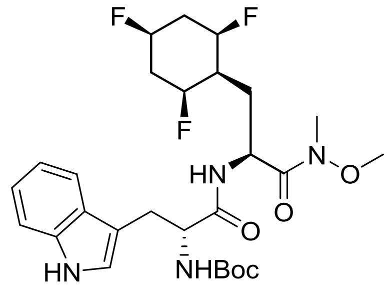

^{1}H NMR (400 MHz, Methanol-d_4)

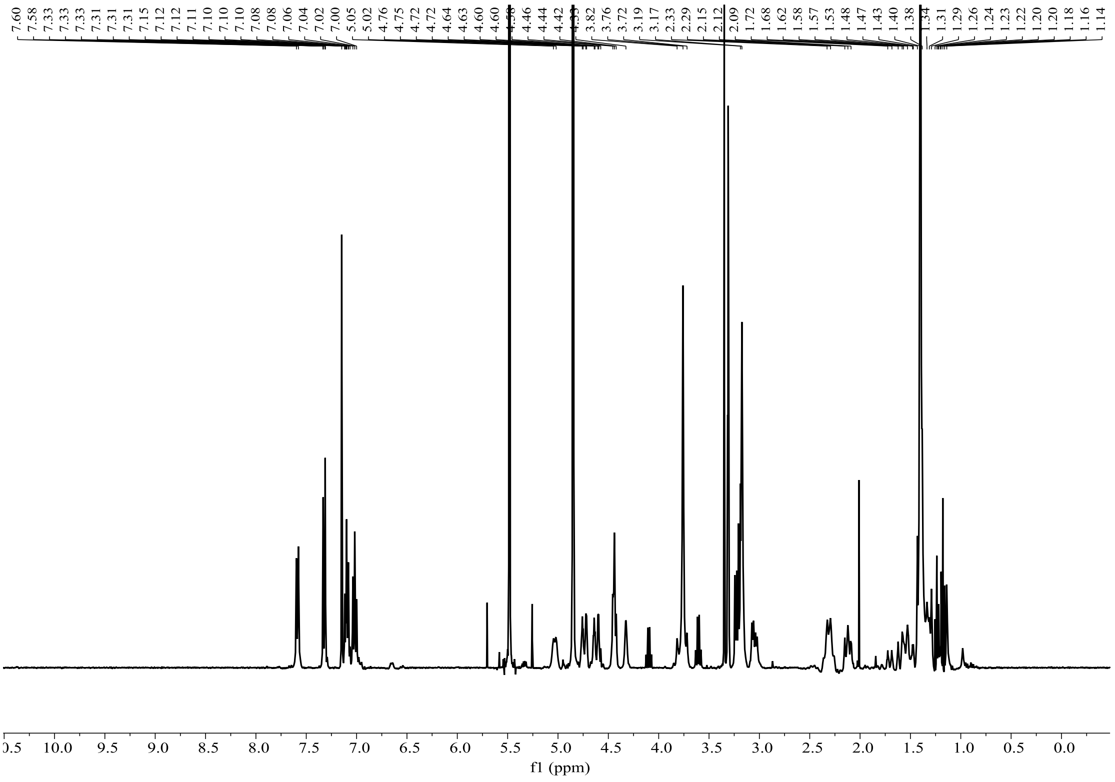

^{13}C NMR (126 MHz, Methanol-d_4)

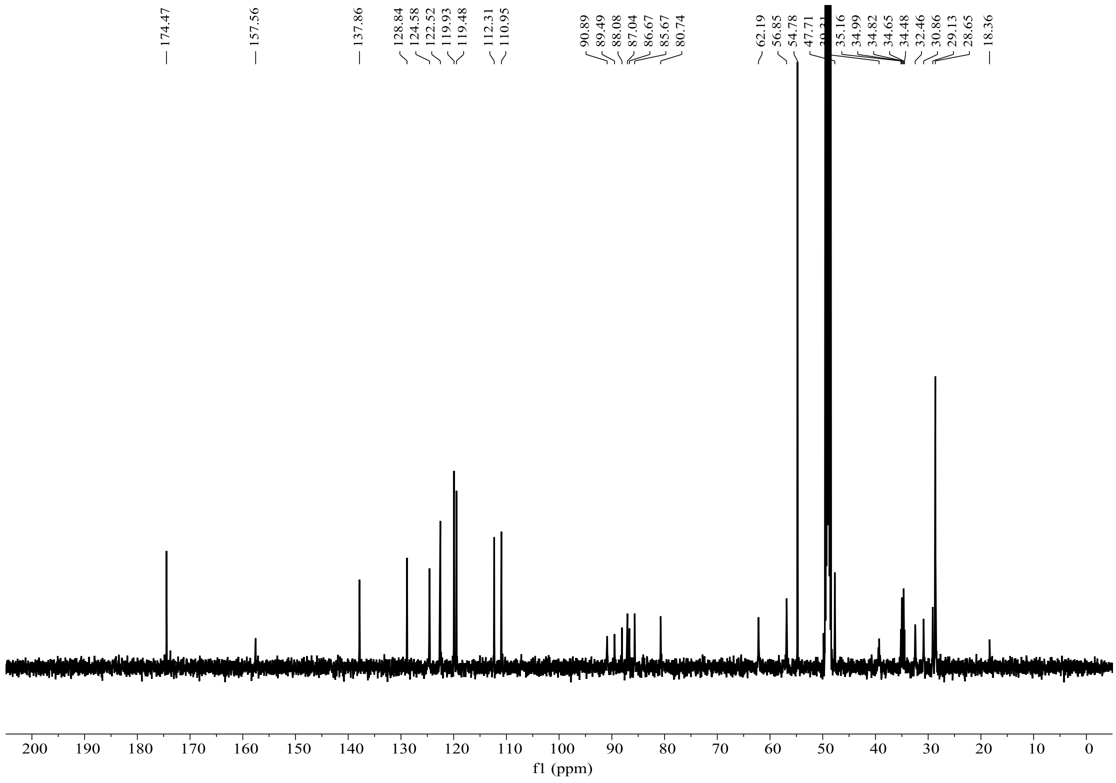

$^{19}F\{^1H\}$ NMR (377 MHz, Methanol-d_4)

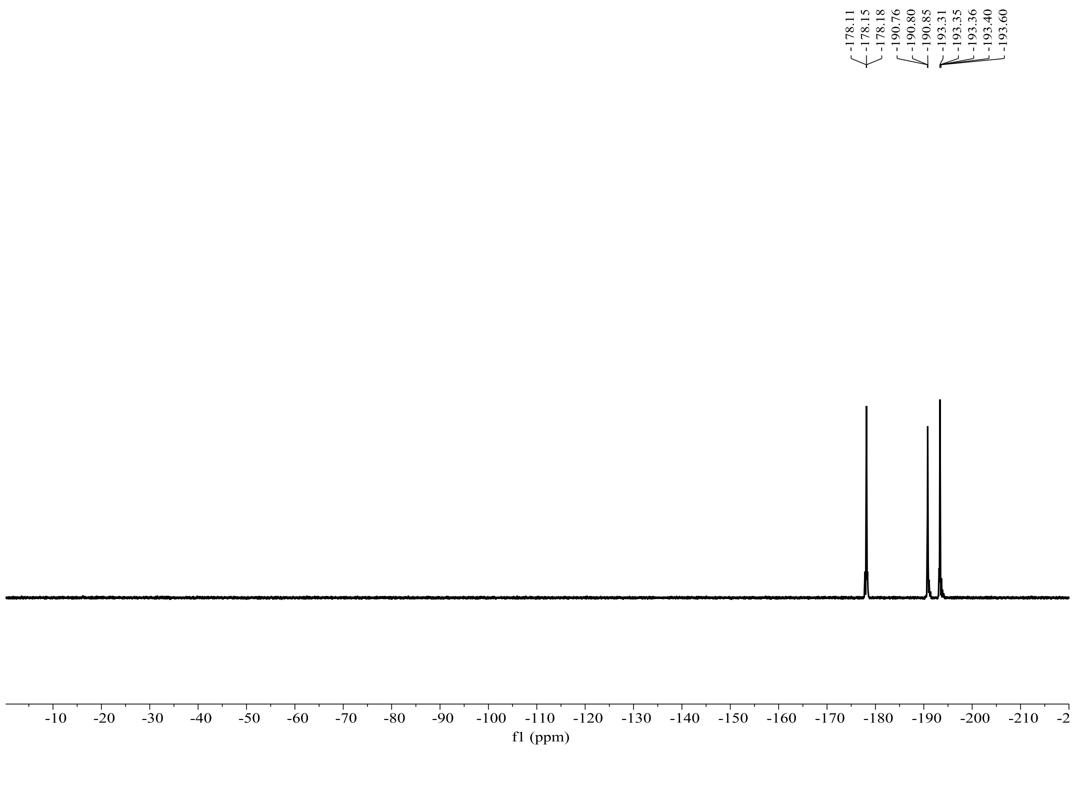

(28d)

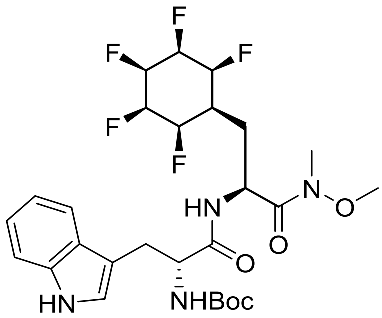

^{1}H NMR (400 MHz, Methanol-d_4)

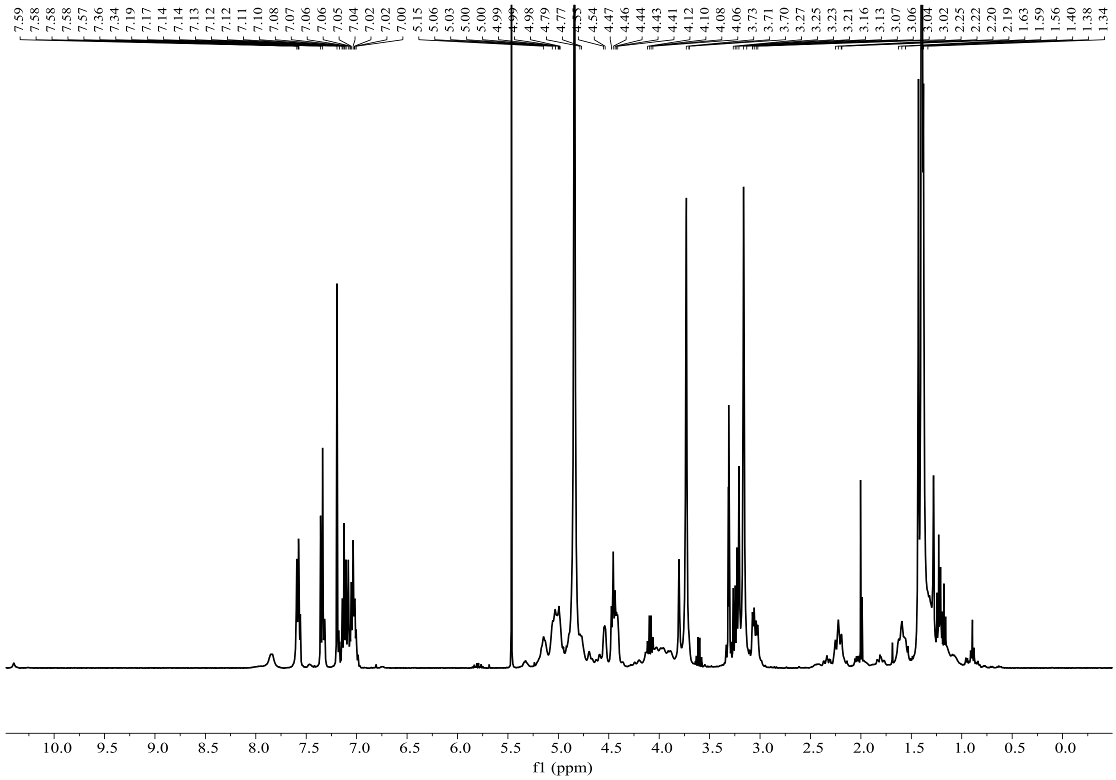

^{13}C NMR (126 MHz, Methanol-d_4)

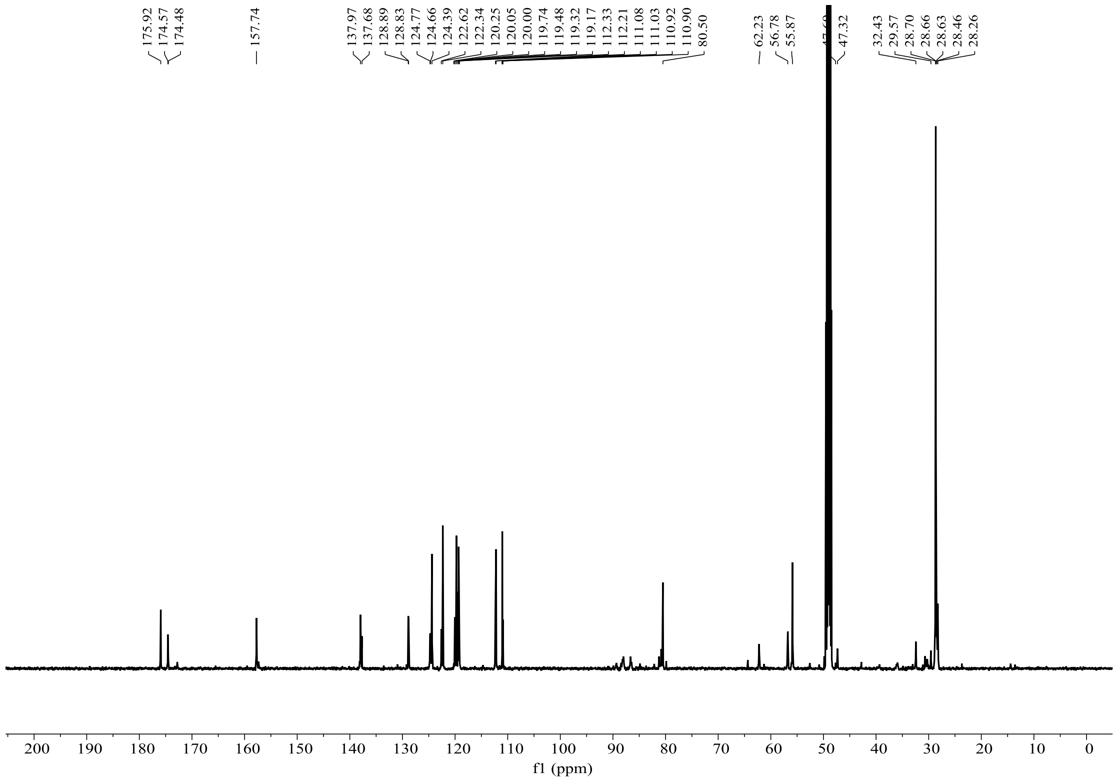

$^{19}F\{^{1}H\}$ NMR (377 MHz, Methanol-d_4)

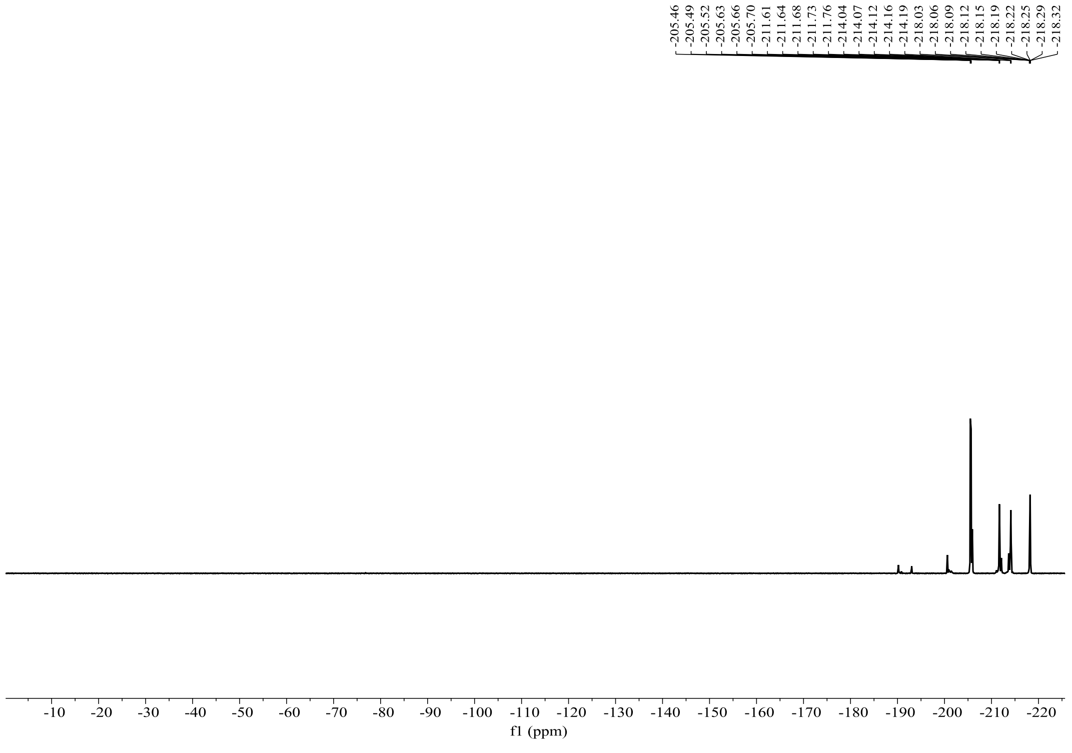

(29a)

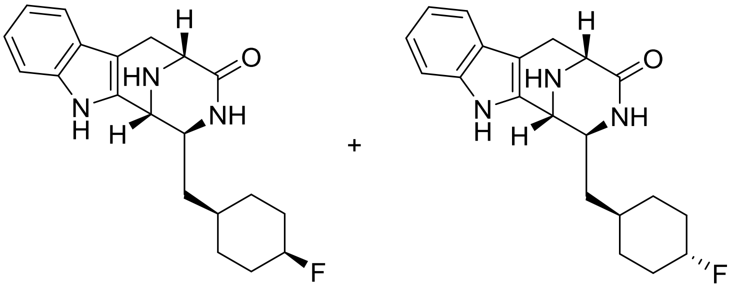

^{1}H NMR (400 MHz, Methanol-d_4)

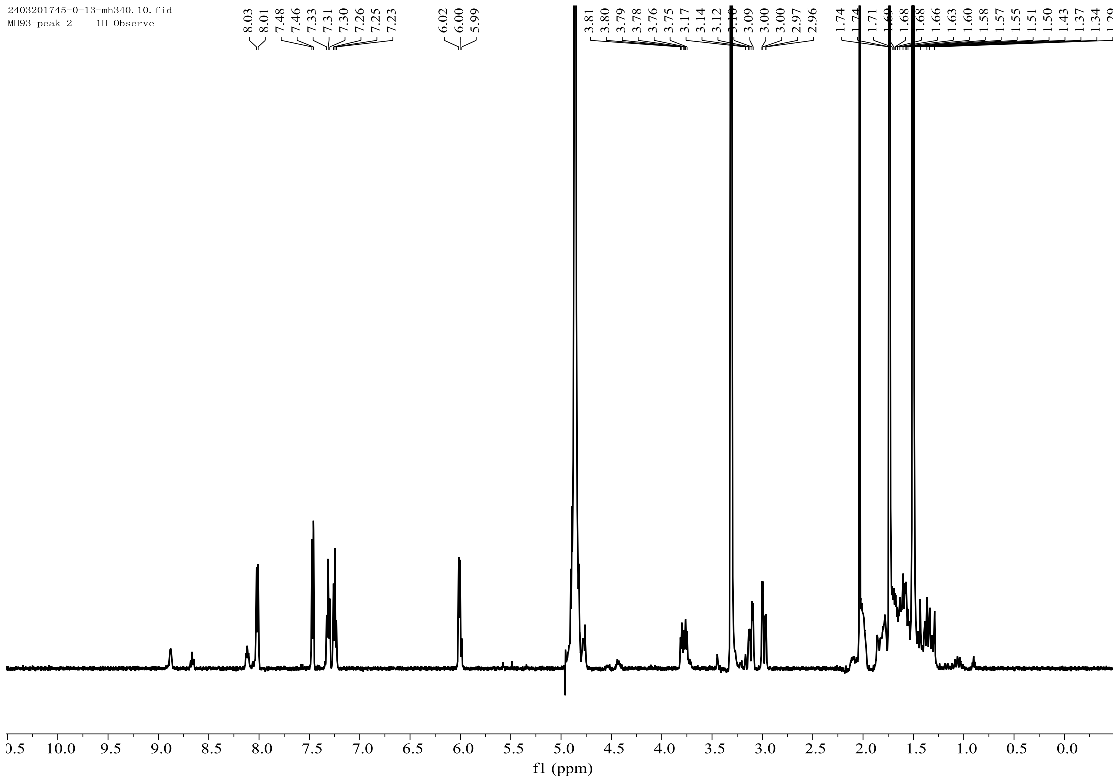

^{13}C NMR (126 MHz, Chloroform-*d*)

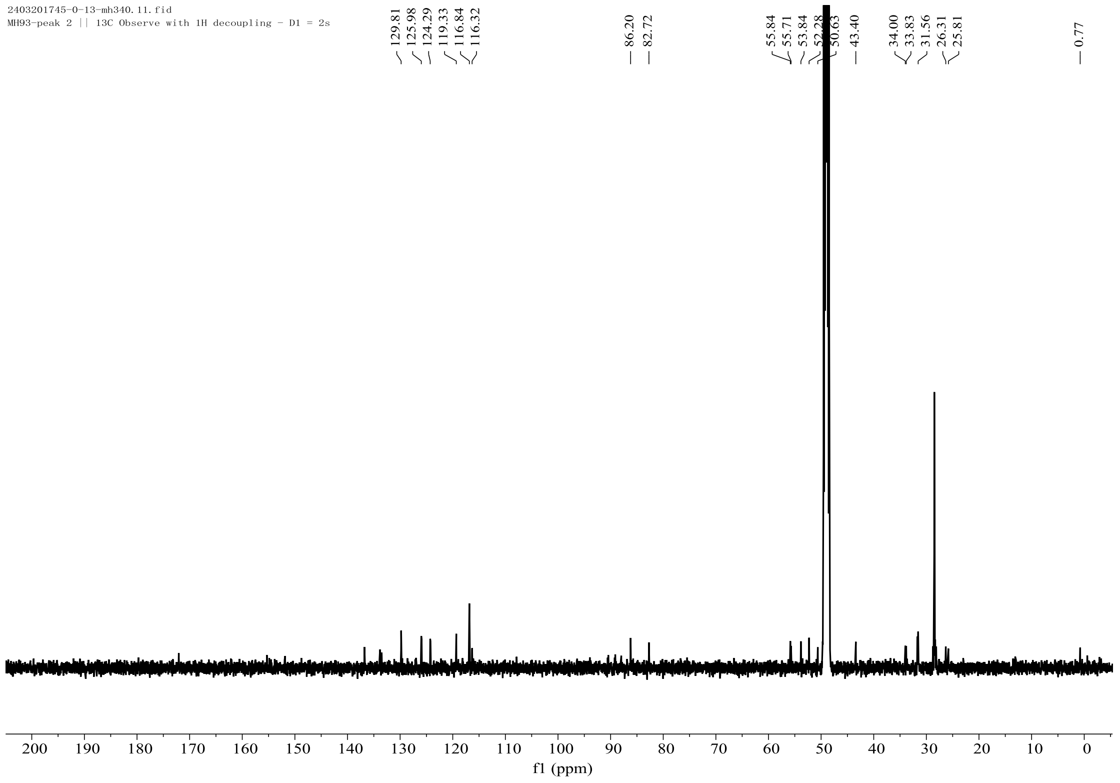

$^{19}F\{^1H\}$ NMR (377 MHz, Methanol-d_4)

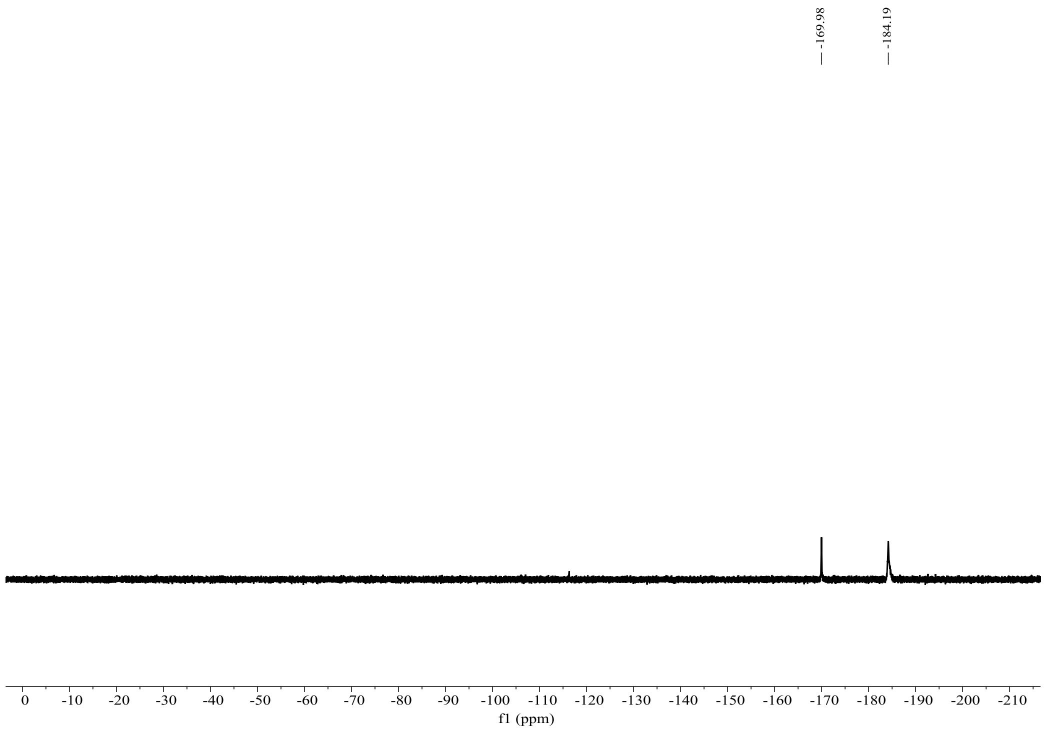

(29b)

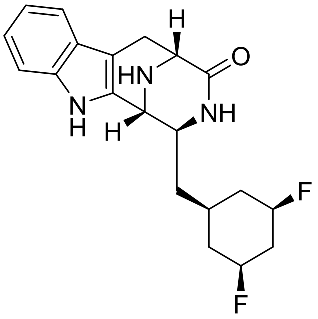

^{1}H NMR (400 MHz, Methanol-d_4)

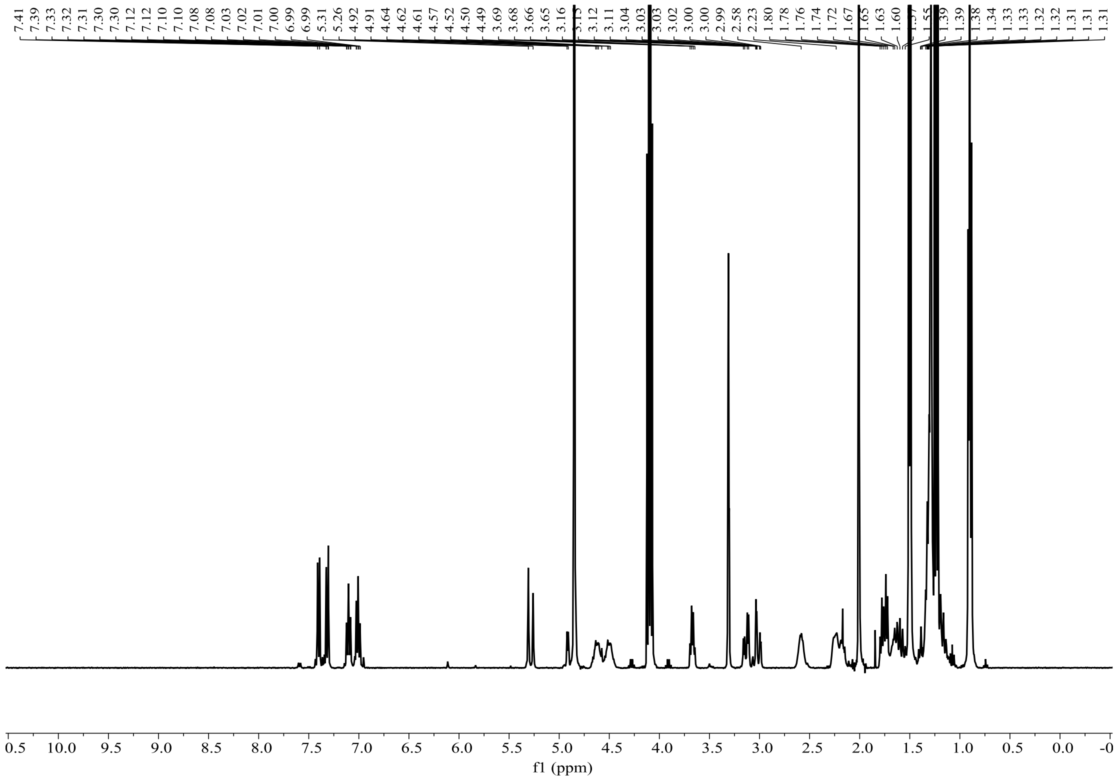

^{13}C NMR (126 MHz, Methanol-d_4)

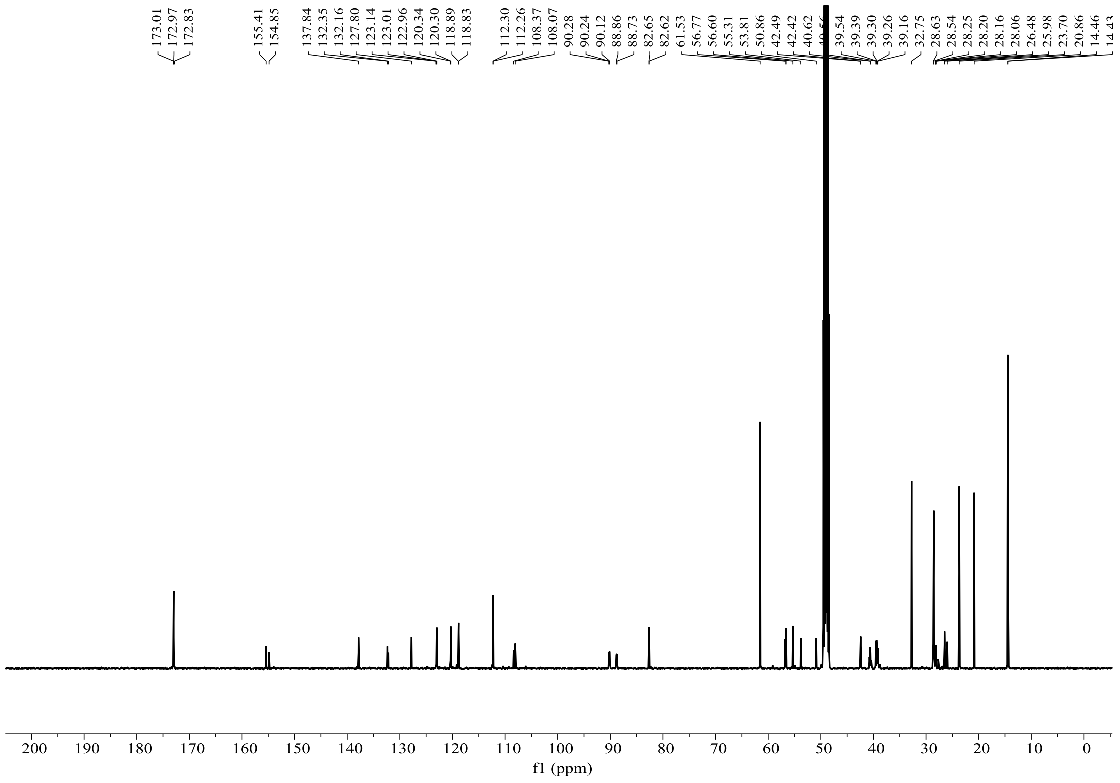

$^{19}F\{^{1}H\}$ NMR (377 MHz, Methanol-d_4)

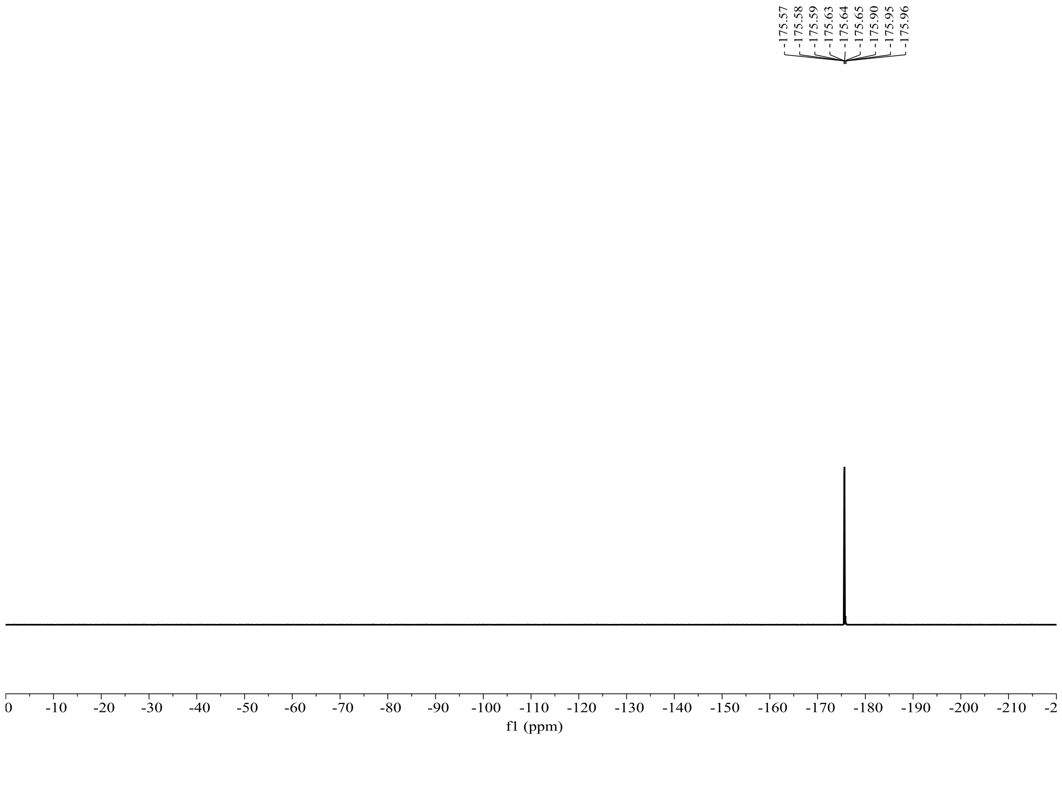

(29d)

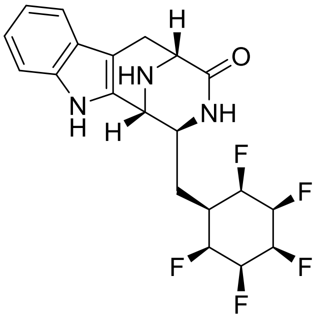

^{1}H NMR (700 MHz, Methanol-d_4)

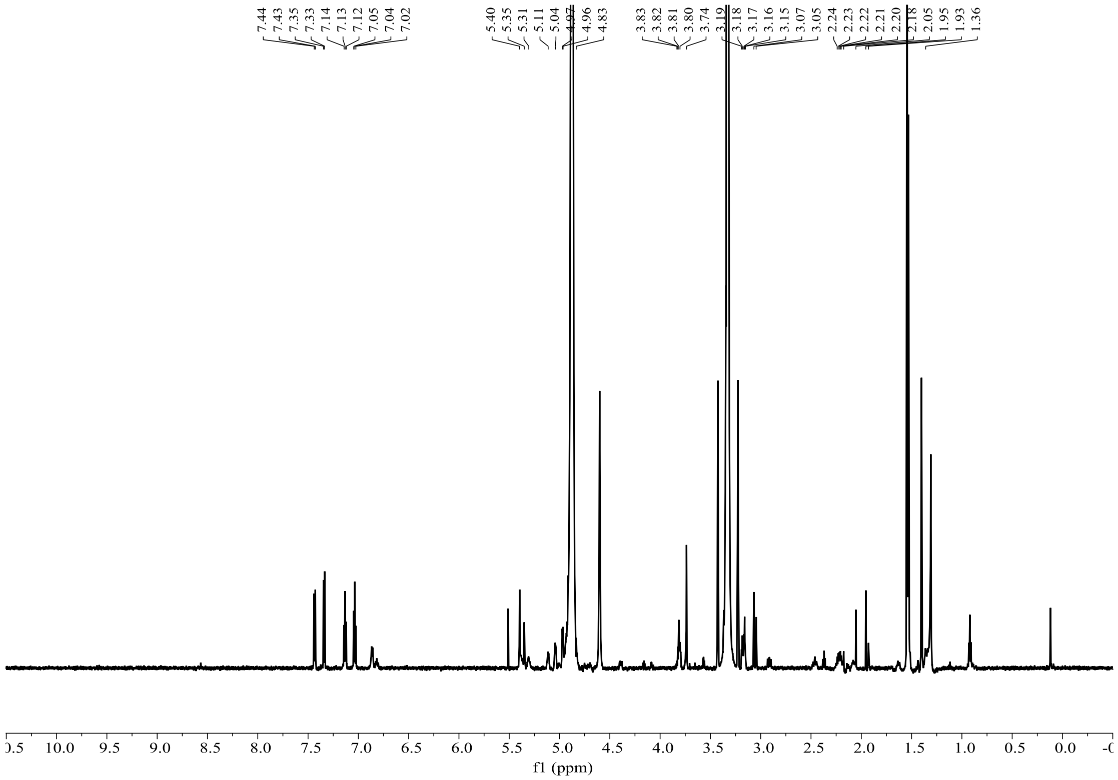

^{13}C NMR (126 MHz, Methanol-d_4)

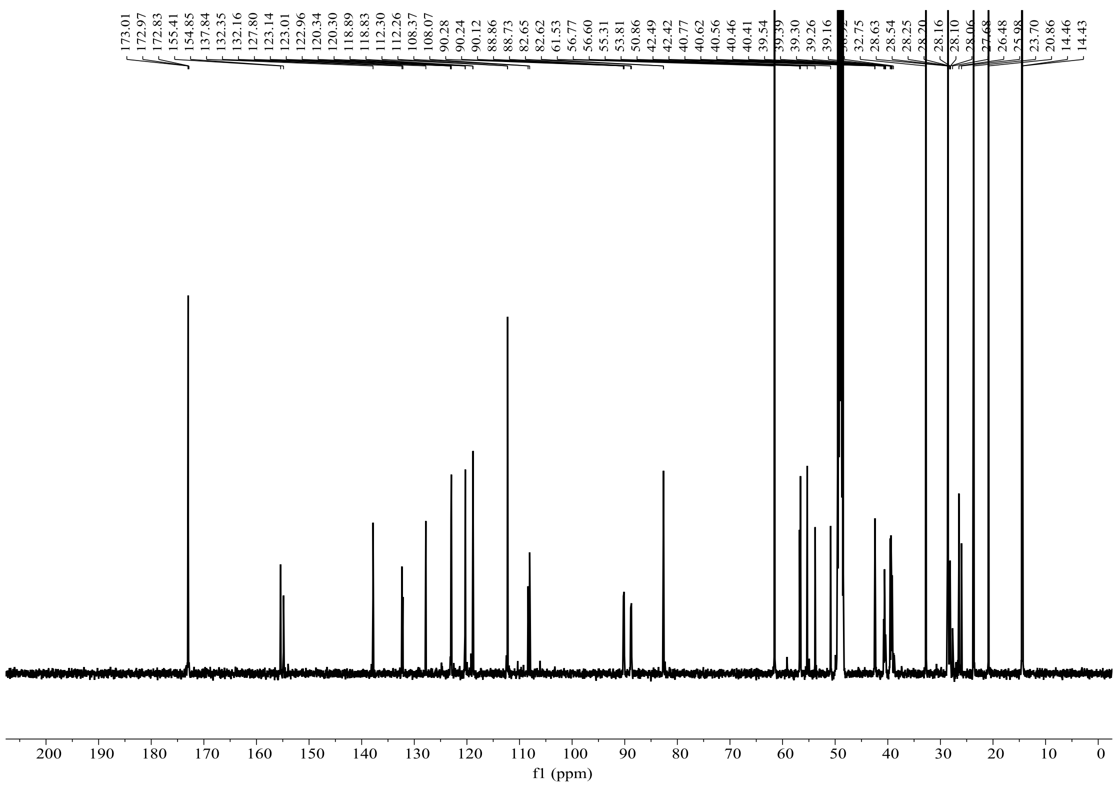

^{19}F{^{1}H} NMR (377 MHz, Methanol-d_4)

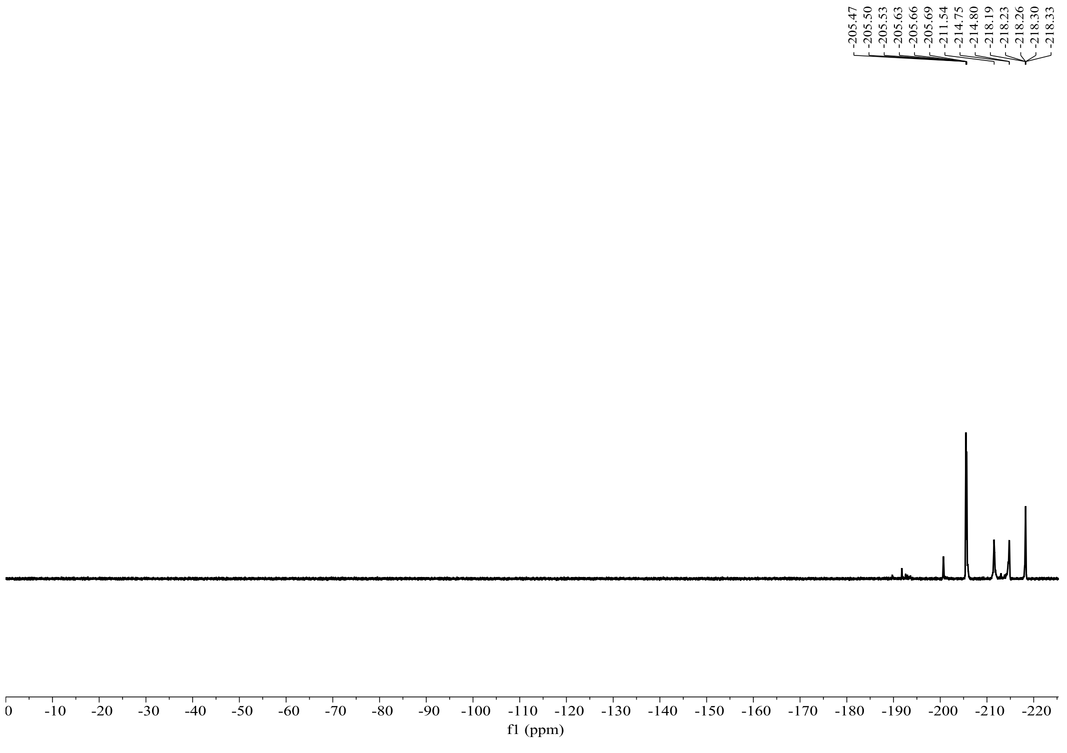

Additional experimental procedures

Synthesis of Janus face W-peptide

Materials and Reagents: All reagents used were of analytical, peptide synthesis or high-performance liquid chromatography grade. 9-Fluorenylmethoxycarbonyl (Fmoc) protected amino acid monomers; Oxyma were obtained from CEM Corporation. Trifluoroacetic Acid (TFA), *N,N'*-Diisopropylcarbodiimide (DIC) and Fmoc-fluoro-tyrosine were obtained from Fluorochem (UK).*N,N'*-Dimethylformamide (DMF); Dichloromethane (DCM); Diethyl Ether (Et_2O); HPLC grade acetonitrile and water were obtained from VWR Chemicals/Avantor (UK). 2,2′-(Ethylenedioxy)diethanethiol (DODT), piperidine and MBHA Rink Amide HL resin were obtained from Sigma Aldrich/Merck (UK).

Peptides synthesis: Peptides were synthesized exploiting Fmoc (*N*-(9-fluorenyl) methoxycarbonyl) solid phase microwave-assisted peptide chemistry using a Liberty Blue™ Automated Microwave Peptide Synthesizer (CEM). The instrument uses a 0.2 M DMF solution of the standard amino acids, 1 M solution of *N*,*N′*-diisopropylcarbodiimide (DIC) as the coupling agent dissolved in DMF and a 1 M solution of ethyl cyano(hydroxyimino)acetate (Oxyma pure) as an additive dissolved in DMF. Fmoc deprotection was achieved by using a solution of 20% piperidine in DMF. For the synthesis of W-peptide and peptide A (StAndMH003, fluorothyrosine) the coupling steps were performed using the CEM CarboMAX strategy that foresees a molar ratio of 1:5:10:5 of Resin/Fmoc-AA-OH/DIC/Oxyma pure.

For the synthesis of peptides **23a-d**, **24**, **25** containing the non-proteinogenic amino acids **22a-d** and mono- or penta- fluoroaryl Fmoc-phenylalanines. The software was edited to include the non-standard amino acids and the synthesis was performed using a modified procedure. The software was set to use a 0.1 mmol scale and reagents were prepared in accordance, but only half of the required amount of resin

was used bringing the real scale of the synthesis to 0.05 mmol and the molar ratio to 1:2:20:10.

Cleavage of the synthesised peptide from the resin and removal of side protecting groups was performed by treating the resin with a cleavage solution (92.5% trifluoroacetic acid (TFA), 2.5% of triisopropylsilane (TIS), 2.5% 2,2′-(Ethylenedioxy)diethanethiol (DODT) and 2.5% of water) for 3 hours at room temperature. TFA was removed by a stream of nitrogen and the peptide precipitated by the addition of cold diethyl ether.

Crude peptides were purified by preparative RP-HPLC using an Agilent 1260 system and a Phenomenex Luna C18(2) preparative column (5 µm, 100 Å, 21mm x250 mm ID x L).

Purity of the peptides was evaluated by HPLC-MS analysis using an Agilent 1200 HPLC equipped with a Diode Array Detector (DAD) and coupled with a single quadrupole mass detector using a Phenomenex Luna C18 analytical column (5 µm, 100 Å, 4.6mm x250 mm ID x L)

Bioactivities assays of Janus face W-peptide

Drugs and Reagents: Coelenterazine H was purchased from Cayman Chemicals. Dubeccos Modified Eagles Medium (DMEM) and heat inactivated foetal calf serum (FCS) were purchased from Fisher Scientific. Polyethyleneimine, MW 40,000 (PEI) was purchased from Polysciences Inc. All other reagents from standard suppliers.

cDNA Constructs: N-terminal signal sequence FLAG-tagged human Formyl Peptide Receptor 2 (FPR2) was generated previously (Thompson et al 2014). C-terminal tagged FPR2 was generated using hiFi DNA assembly (NEB) based on RLuc8 (a gift from Nevin Lambert, Augusta University). Bioluminescence Resonance Energy Transfer (BRET) constructs were kind gifts from Kevin Pfleger (The University of Western Australia).

Cell Culture and Transfection: Constructs were either transiently or stably expressed in native Human Embryonic Kidney 293 (HEK293) cells and maintained in DMEM containing 10% FCS. Transfections were performed using PEI at a ratio of DNA:PEI of 1:4 for all studies.

BRET analysis of β-Arrestin and mini-Gsi recruitment: HEK293 cells were grown on 6 well plates until 90-95% confluency was reached. For measurement of direct interaction, 400ng of FPR2-RLuc8 (donor) was co-transfected with venus-tagged (acceptor) mGsi or β-Arrestin 2 at a ratio of 4:1 (Acceptor:Donor). The following day, cells were replated and seeded into white opaque 96-well plates. Cells were assayed 221ah post-transfection as described previously [1,2]. Briefly, cells were incubated at 37ºC in HBSS for 30-40mins followed by 5µM Coelenterazine H for 10 mins. Cells were then stimulated with increasing concentrations of WKYMVm and analysed using ClarioStar Plus plate reader (BMG Labtech) at 10 minutes post agonist addition. Luminescence and fluorescence values were measured at 460/30 nm (donor) and 480/40 nm (acceptor), and the ratio of acceptor:donor determined and normalised by subtraction of the ratio observed in untreated cells.

Competition Binding: Competition binding was performed using a fluorescently labelling ligand using an assay analogous to the classical radiolabelled method.[1] Briefly, human embryonic kidney 293 (HEK293) cells stably expressing human Formyl peptide receptor 2 (FPR2) were added to a mixture of 10nM FITC-WKYMVm and varying concentrations (0-10µM) of competing ligands (WKYMVm **13**, **23a-c**, **24**, **25**) and incubated for 90 min on ice in the dark to allow equilibrium binding. Subsequently, mean fluorescence was measured by flow cytometry using the Attune NxT. Data was expressed as a percentage of maximum binding (i.e. no competitor) and analysed via nonlinear regression one site model of equilibrium. IC50 values were determined using GraphPad PRISM software.

Bioactivities assays of Janus face Tryptophan keto-piperazines

Parasites were grown in different medium (*e.g*. T. brucei was grown in HMI-11 medium) with 10% heat-inactivated foetal bovine serum. parasites were counted using CASY TT Cell Counter. Fluorescence was recorded using a FLx 800 plate reader (BioTek) with an excitation wavelength 530–535 nm and emission wavelength of 590–610 nm using Gen5 Reader Control 2.0 Software (BioTek). EC_{50} values were determined using a 4-parameter non-linear logistic regression equation using GraFit 5.0 (Erithacus Software). SD values were calculated based on curve fitting to n biological replicates performed in parallel.

References

[1] D. Thompson, S. McArthur, J. N. Hislop, R. J. Flower, M. Perretti, M. '*Identification of a Novel Recycling Sequence in the C-tail of FPR2/ALX Receptor.*' *J Biol Chem*., **2014**, *289*, 36166–36178.

[2] D. Thompson, L. Martini, J. L. Whistler, JL. '*Altered Ratio of D1 and D2 Dopamine Receptors in Mouse Striatum Is Associated with Behavioral Sensitization to Cocaine*.' *PLoS One.,* **2010,** *5*, e11038.
